# Characterization of a Novel Glutamine Synthetase From *Trichinella spiralis* and Its Participation in Larval Acid Resistance, Molting, and Development

**DOI:** 10.3389/fcell.2021.729402

**Published:** 2021-09-20

**Authors:** Tong Xu Zhuo, Zhen Wang, Yan Yan Song, Shu Wei Yan, Ruo Dan Liu, Xi Zhang, Zhong Quan Wang, Jing Cui

**Affiliations:** Department of Parasitology, Medical College, Zhengzhou University, Zhengzhou, China

**Keywords:** *Trichinella spiralis*, glutamine synthetase, molting, acid resistance, RNAi

## Abstract

*Trichinella spiralis* is a major foodborne parasite worldwide. After the encapsulated muscle larvae (ML) in meat are ingested, the ML are liberated in the stomach of the host and activated into intestinal infectious larvae (IIL), which develop into adult worm after molting four times. A novel glutamine synthetase (TsGS) was identified from *T. spiralis* IIL at 10 h post-infection, but its biological role in *T. spiralis* life cycle is not clear. The aim of this study was to investigate the biological characteristics of TsGS and its functions in larval acid resistance, molting, and development. TsGS has a glutamine synthetase (GS) catalytic domain. Complete TsGS sequence was cloned and expressed in *Escherichia coli* BL21. rTsGS has good immunogenicity. qPCR and Western blotting showed that TsGS was highly expressed at IIL stage, and immunofluorescence revealed that TsGS was principally localized at the cuticle and intrauterine embryos of this nematode. rTsGS has enzymatic activity of natural GS to hydrolyze the substrate (Glu, ATP, and NH_4_^+^). Silencing of TsGS gene significantly reduced the IIL survival at pH 2.5, decreased the IIL burden, and impeded larval molting and development. The results demonstrated that TsGS participates in *T. spiralis* larval acid resistance, molting and development, and it might be a candidate vaccine target against *Trichinella* molting and development.

## Introduction

*Trichinella spiralis* is a serious foodborne zoonotic parasitic nematode and the principal causative agent of human trichinellosis. *T. spiralis* infection in humans is mainly resulted from eating poorly cooked or raw infected pork in most countries (Cui et al., 2013a; Jiang et al., 2016; Rostami et al., 2017). Various *T. spiralis* developmental stage worms are parasitized within the same host; adult worms (AW) and muscle larvae (ML) dwell in the duodenum and upper jejunum and skeletal muscles, respectively. After ingestion, the ML encapsulated in muscle tissues are released from their collagen capsules under the digestion of gastric pepsin and activated into intestinal infectious larvae (IIL) after being contacted with enteral contents or bile (Ren et al., 2011; Liu et al., 2013). The IIL intrude intestinal epithelium cells (IECs) and develop to adulthood in gut epithelial intramulticellular niche after going four molts. Molting is a prerequisite step for larval growth and development of the parasitic nematode. If their molting could not be completed, the larvae cannot continue to develop and grow (Gagliardo et al., 2002; Ren et al., 2013). Therefore, larval molting-related proteins are likely the potential targets to develop new drugs or vaccines against nematode infection.

Glutamine synthetase (GS) is widely distributed in microorganisms, animals, and higher plants. It participates in many biological processes and is regarded as an ideal evolutionary molecular clock (Kumada et al., 1993). GS produces glutamine using glutamate and ammonia in the presence of ATP and Mg^2 +^. Glutamine is the carbon and nitrogen donor for the production of biomolecules and participates in the dynamic balance of redox. GS is the key enzyme of nitrogen metabolism in organisms. The physiological process of nitrogen assimilation is very important in the process of organism growth and development. GS is a major enzyme involved in ammonia assimilation. In the presence of Mg^2+^ and energy supplied by ATP, GS catalyzes the assimilation of NH_4_^+^ to glutamine. Glutamine produces glutamate under the catalysis of GS. In nitrogen metabolism, glutamine serves not only as a temporary storage and transport form of toxic glutamic acid and ammonia but also as a nitrogen donor of amino acids, nucleic acids, and hexosamine. Although there are many metabolic pathways of glutamine, GS is the only enzyme in the *de novo* synthesis of glutamine. GS is also an indispensable detoxifying enzyme in stress and immune response (Lai et al., 2011; Wang et al., 2014). In *Trypanosoma cruzi*, GS is necessary to resist ammonium accumulation toxicity and immune escape during host cell infection (Crispim et al., 2018). However, there are no reports on the biological properties and functions of the GS in the life cycle of *T. spiralis* in the literatures.

*Escherichia coli* is a kind of bacteria which is closely related to the daily life of people. As a foodborne pathogen, *E. coli* must pass through the extremely sour stomach of mammals to cause a disease. *E. coli* is resistant to very low pH levels and can survive at pH 2.5 for more than 2 h, mainly due to complex acid-resistant systems (Jin et al., 2009). At present, there are four main anti-acid systems known: glucose-inhibited acid-fast system, glutamate-dependent acid-fast system, arginine-dependent acid-fast system, and glutamine-dependent acid-fast system (AR4). When bacteria are in acidic conditions, they will induce the synergistic action of multiple acid-resistant systems (ARs) in hosts so as to survive in acidic conditions. Organisms survive within the appropriate pH range. In physiological and biological buffering systems, ammonia and carbon dioxide mainly regulate the pH (Krulwich et al., 2011). The pathogenicity of foodborne pathogens determines the inevitability of their acid tolerance. Stimulation in an acidic environment leads to the expression of acid-resistant genes in pathogens (Djoko et al., 2017). *T. spiralis* is a foodborne parasite, and the ML might have acid-resistant genes to protect the larvae from gastric acid damage (Zhang et al., 2010). AR4 is one of the most important acid-resistant systems in *E. coli*. Recent studies showed that glutaminase is an important gene in glutamine-dependent acid resistance of *T. spiralis* ML (Gao et al., 2021). However, there are no reports on the GS role in *T. spiralis* ML acid resistance.

In our previous study, a novel *T. spiralis* GS (TsGS; GenBank: XM_003374954.1) was identified in somatic crude proteins of 10-h IIL in the process of first larval ecdysis (Ren et al., 2019). The aim of this study was to investigate the biological properties and roles of TsGS in larval acid resistance, molting, and development of *T. spiralis*.

## Materials and Methods

### Parasites, Animals, and Antigens

The parasite *T. spiralis* (ISS534) was acquired from an infected domestic pig in central China (Wang et al., 2012) and maintained in our laboratory by serial passage in BALB/c mice. Female BALB/c mice of 4–6 weeks old were purchased from Henan Provincial Experimental Animal Center (Zhengzhou, China). The ML was recovered by artificial digestion of infected mouse carcasses at 42 days post-infection (dpi) with 0.33% pepsin (1:31,000; Sigma-Aldrich, United States) and 1% HCl (Jiang et al., 2012). The IIL and AW were collected from infected murine small intestine (Sun et al., 2015a,b). The adult females at 6 dpi were cultured in RPMI-1640 with 10% fetal bovine serum (Gibco, Auckland, New Zealand) at 37°C in 5% CO_2_ for 24 h, and the newborn larvae (NBL) were harvested as previously described (Li et al., 2015; Wu et al., 2016). The somatic crude proteins of diverse *T. spiralis* phases (ML, IIL, AW, and NBL) and IIL ES proteins were prepared as reported before (Yang et al., 2015).

### Bioinformatics Analysis of TsGS

The complete cDNA sequence of the TsGS gene was obtained from GenBank (GenBank: XM_003374954.1), and its physicochemical characteristics and structures were analyzed and predicted by NCBI online website. Multi-sequence alignment was carried out between TsGS and GS from *Trichinella* spp. by using Clustal X (Li et al., 2015). The GenBank accession numbers of GS from *Trichinella* spp. and other organisms were as follows: *Trichinella nativa* (KRZ52922.1), *Trichinella britovi* (KRY49640.1), *Trichinella* T6 (KRX74386.1), *Trichinella* T8 (KRZ85490.1), *Trichinella* T9 (KRX59460.1), *Trichinella murrelli* (KRX49151.1), *T. patagoniensis* (KRY23667.1), *Trichinella zimbabwensis* (KRZ13164.1), *Trichinella papuae* (KRZ68830.1), *Trichinella pseudospiralis* (KRZ22405.1), *Mus musculus* (NP_032157.2), and *Homo sapiens* (NP_001028228.1). The phylogenetic tree was constructed using MEGA 7.0 based on the neighbor-joining method as reported previously (Qi et al., 2018b; Hu et al., 2020).

### Cloning, Expression, and Identification of rTsGS

Total RNA was extracted from the 6-h IIL using Trizol (Invitrogen, United States), and *Eco*RI and *Sal*I (italicized) were used as restriction sites to design TsGS-specific primers (5′-CCGG *AATTC*ATGGCATTCACGTTGACATTGAGCT-3′ and 5′-CCGACG*TCGAC*TTACTCTCTT AAACAAA TGGTACGCACC-3′). The complete TsGS cDNA sequence was amplified by PCR. The PCR product was cloned into the pET-28a carrying a His-tag at N-terminus (Novagen, United States), and recombinant pET-28a/TsGS was transformed into *E. coli* BL21 (Novagen). The expression of rTsGS was induced with 0.5 mM isopropyl β-D-1-thiogalactopyranoside at 30°C for 8 h (Xu et al., 2018). A Ni-NTA-Sefinose resin containing His tag (Sangon Biotech, Shanghai, China) was used to purify rTsGS (Sun et al., 2018). The expression of rTsGS protein was analyzed by SDS-PAGE as described above (Xu et al., 2020).

### Preparation and Measurement of Anti-rTsGS Antibodies

Twenty mice were immunized subcutaneously with 20 μg rTsGS mixed with complete Freund’s adjuvant. Boost immunization was performed three times with 20 μg rTsGS mixed with incomplete Freund’s adjuvant at a 2-week interval (Zhang et al., 2020). At 2 weeks after the last immunization, the tail blood of immunized mice was collected to isolate anti-rTsGS immune sera (Cui et al., 2013b).

The anti-rTsGS IgG level of all immunized mice was assessed by ELISA (Cui et al., 2015b). Briefly, micro-plates were coated with 2 μg/ml rTsGS at 37°C for 2 h. After washing with phosphate-buffered saline (PBS; pH 7.4) containing 0.05% Tween-20, the plate was blocked with 5% skimmed milk at 37°C for 2 h. Following a wash, the diluted anti-rTsGS sera were added and incubated at 37°C for 2 h and then by incubation of HRP-conjugated anti-mouse IgG (1:10,000; Southern Biotech, United States) at 37°C for 1 h. Coloration was performed using OPD (Sigma-Aldrich) plus H_2_O_2_; the reaction was finished by the addition of 2 M H_2_SO_4_. The optical density values at 492 nm were measured by a microplate reader (Tecan Schweiz AG, Switzerland) (Liu L. N. et al., 2015).

### Western Blotting Analysis

Soluble crude proteins from diverse *T. spiralis* phase worms, 6-h IIL ES proteins, and rTsGS were separated by 12% SDS-PAGE (Wang et al., 2013; Ren et al., 2018). The proteins were transferred onto a nitrocellulose membrane (Millipore, United States). The membrane was blocked with 5% skimmed milk diluted in Tris-buffered saline (pH 7.4) containing 0.05% Tween-20 (TBST) at 37°C for 2 h and cut into strips. The strips were probed with 1:100 dilutions of various sera (anti-rTsGS serum, infection serum, and normal serum) at 37°C for 2 h. After washing with TBST, the strips were incubated with HRP-conjugated anti-mouse IgG (1:10,000; Southern Biotech, United States) at 37°C for 1 h. After washing again, the strips were developed with 3,3′-diaminobenzidine tetrahydrochloride (Sigma-Aldrich) or using an enhanced chemiluminescent kit (CWBIO, Beijing, China) and terminated by washing the membrane with deionized water (Long et al., 2014; Liu et al., 2018).

### qPCR

Total RNAs from diverse *T. spiralis* phases (ML, 6- and 10-h IIL, 3-day AW, and NBL) were extracted with Trizol (Invitrogen). The TsGS transcription levels at various stage worms were ascertained by qPCR as described before (Long et al., 2015; Hu et al., 2021). The TsGS-specific primers for qPCR amplification were as follows: 5′-AGTGACTGGAATGTTTGGAGA-3′ and 5′-AATCAGTTCCTGTGATGCC-3′. The relative TsGS transcription level was normalized by subtracting the transcription of a *T. spiralis* housekeeping gene tubulin (GenBank: XM_003369432.1; 5′-TGCATTGGTACACTGGAGAAG-3′ and 5′-GCTTCCTGGTACTGCTG ATATT-3′) (Yang et al., 2010) and then calculated according to comparative Ct (^2–ΔΔCt^) method (Xu et al., 2021). Each experiment had three repeats.

### Indirect Immunofluorescence Assay

Whole worms of different *T. spiralis* stages (ML, IIL, AW, and NBL) were fixed with 4% formaldehyde for 30 min and re-fixed with cold acetone for 20 min. The fixed worms were embedded in paraffin and cut into a 2-μm-thick cross-section with a microtome. The expression and tissue location of native TsGS in various worm stages were investigated by indirect immunofluorescent assay (IIFA) (Liu et al., 2017; Cui et al., 2019). Whole parasites and their cross-sections were blocked with 5% normal goat serum for 2 h and then probed at 37°C for 2 h with 1:10 diluted diverse sera (anti-rTsGS serum, infection serum, and normal serum). After washes with PBS, the worms were stained using goat anti-mouse IgG-FITC conjugate (1:100; Santa Cruz, United States). Following washes again, whole worms and cross-sections were examined under a fluorescence microscope (Olympus, Tokyo, Japan) (Lei et al., 2020; Yue et al., 2020).

### Measurement of rTsGS Enzyme Activity

Since rTsGS was expressed in the form of inclusion body, rTsGS was first re-naturated by dilution refolding method (Li et al., 2009; Liu L. N. et al., 2015). To assess the enzyme activity of rTsGS, the diluted rTsGS (0.05–1.00 μg/μl) was incubated at 35°C for 15 min in various pH buffers (pH 3.0–10.5). Subsequently, the GS substrates (Glu, ATP, and NH_4_^+^) were added to the reaction mixture and incubated at different temperatures (20–70°C) for 30 min, and the absorbance at 660 nm was measured with a spectrophotometer. In order to evaluate the effect of different metal ions on the rTsGS enzyme activity, the metal ions (Mg^2+^, Zn^2+^, Mn^2+^, Ca^2+^, Fe^2+^, Co^2+^, Cu^2+^, and Ni^2+^) were added to the reaction system. Different enzyme inhibitors (1 mM AEBSF, 5 μM E-64, 10 μM EDTA, and 10% glufosinate) were used to ascertain the effects of the inhibitors on rTsGS enzyme activity. According to the change of absorbance at OD_660_ of KH_2_PO_4_ standard solution with a concentration of 0.1–1.0 mM, the standard curve of phosphorus was calculated. The concentration range of monosodium glutamate (Glu), ATP, and ammonium (NH_4_^+^) was set to 0.5–5 mM. The Km value was obtained from Michaelis–Menten curve and Lineweaver–Burk curve (Cui et al., 2015a; Guo et al., 2020).

The enzymatic activity of natural TsGS in somatic proteins of various worm stages was assessed by using the Micro GS Assay Kit (Solarbio, Beijing, China) (Wang et al., 2018).

### RNA Interference

TsGS-specific siRNA-356 (5′-UCAAUGUCAACGUGAAUGC CA-3′ and 5′-GCAUUCACGU UGACAUUGAGC-3′) was designed according to the full-length TsGS cDNA sequence and prepared by Sangon Biotech (Shanghai, China). Additionally, a control siRNA with a scrambled sequence (5′-AUCGGCUACCAAGUCAUACTT-3′ and 5′-GUAUGACUUGGUAGCCGAUTT-3′) was also synthesized. The control siRNA was fluorescently labeled with 5-carboxyfluorescein (FAM; Sangon Biotech) and used to detect the transfection efficiency. The siRNA-356 is transfected into the ML by electroporation; all the silenced and non-silenced ML were cultured for 1–3 days in RPMI-1640 at pH 7.4, 37°C, and 5% CO_2_ (Wang et al., 2015). The TsGS transcription and expression levels in the ML after RNAi were determined by qPCR and Western blotting as described before (Yang et al., 2019; Yi et al., 2020). A *T. spiralis* housekeeping gene tubulin was used as an internal control. The protein expression level was measured based on densitometry, and the data showed the relative protein expression assessed in three repeated experiments (Xu et al., 2021). The TsGS activity in the crude extract of the siRNA-treated ML was determined using a Micro GS Assay Kit (Solarbio) (Wang et al., 2018), and the results were compared with the untreated ML.

### The Muscle Larvae Acid Resistance Assay

To investigate the effect of acidic conditions on the survival of *T. spiralis* ML, 100 ML were cultured at pH 2.5, 4.5, and 7.4, 37°C, and 5% CO_2_ for 2.5, 12, and 18 h. The survival of the ML cultured in PBS with various pH values was examined under a microscope on the basis of larval viability. Inactive, straight, or C-shaped larvae were assessed as dead larvae. The mobile and wriggly larvae were counted as live ones (Yang et al., 2020; Gao et al., 2021). Somatic crude extracts from the ML cultured at various pH values for 18 h were prepared, and TsGS enzymatic activity was assessed as described before (Wang et al., 2018). In order to evaluate the effects of glutamine on larval survival under acidic condition, the ML were cultivated in PBS supplemented with various doses of glutamine under pH 2.5 condition. Besides this, the acid resistance of the ML transfected with siRNA-356 was also ascertained; the siRNA-356-treated ML was cultured under different pH for various times, and the larval survival was examined under a microscope (Zhang et al., 2016).

### Challenge Infection Experiment With siRNA-356-Treated Larvae

To investigate the larval infectivity, molting, and development, survival, and fecundity in the small intestine of infected mice after RNAi treatment, 80 mice were divided into four groups (20 animals per group). Each mouse was infected orally with 500 ML treated with 3 μM siRNA-356, control siRNA, only inhibitor, or PBS alone. Ten infected mice from each group were euthanized at 24 h post-infection (hpi) and 6 dpi, respectively. Intestinal IIL and AW were recovered and numbered, and the intestinal worm burdens of IIL and AW were assessed. Their morphology and size were examined on a microscope (Liu P. et al., 2015; Qi et al., 2018a). Female adults from each group of infected mice were cultivated, and female reproduction capacity (fecundity) was assessed in accordance with NBL production by each female in 72 h (Sun et al., 2019; Guo et al., 2020).

### Statistical Analysis

All the data were processed by SPSS 21.0 software. The data were presented as mean ± standard deviation (SD). TsGS expression level, worm burdens and length, and female fecundity among various groups was analyzed with one-way ANOVA. Chi-square test was used to analyze larval survival at different pH values. *P* < 0.05 is regarded as statistically significant.

## Results

### Bioinformatics Analysis of TsGS

**FIGURE 1:**
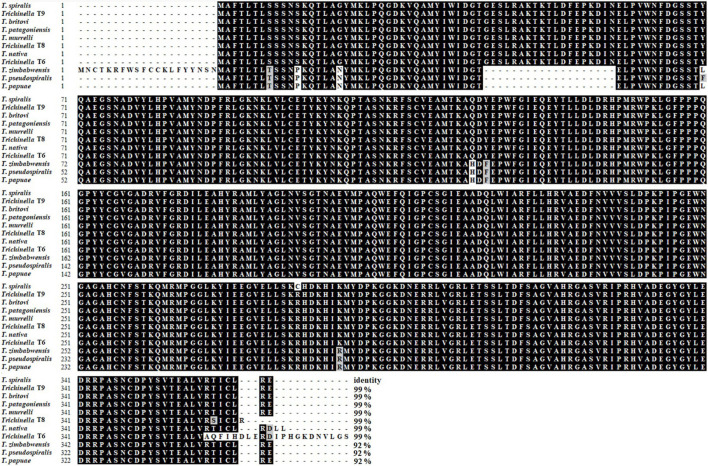
Sequence alignment of TsGS with other species or genotypes of the genus *Trichinella*. Multiple sequence alignment was carried out by using BioEdit, the black shadow represents the same residue as TsGS, and the gray shadow is a conservative substituent. The number at the end of each sequence represents the percentage of identity with TsGS.

**FIGURE 2:**
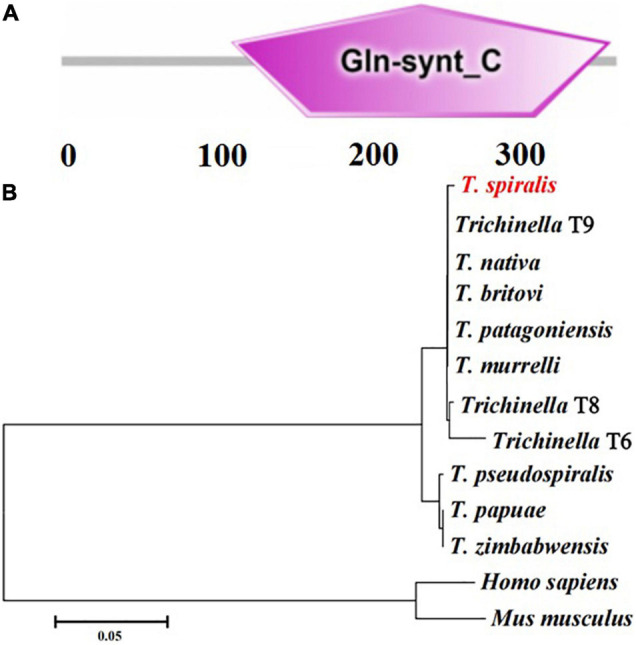
The predicted TsGS catalytic domain (Gln-synt_C) **(A)** and phylogenetic trees of glutamine synthetase of 13 organisms with neighbor-joining method **(B)**.

The complete TsGS sequence was 1,098 bp encoding 365 amino acids and with a molecular weight (MW) of 41.1 kDa and pI 6.71. The amino acid sequence of TsGS had an identity of 99% from seven encapsulated *Trichinella* species (T9, *T. britovi*, *T. patagoniensis*, *T. murrelli*, T8, *T. nativa*, and T6) and 92% of three non-encapsulated species (*T. zimbabwensis*, *T. pseudospiralis*, and *T. papuae*) (Figure 1). TsGS had no obvious hydrophobicity and no signal peptide sequence and transmembrane domain. TsGS had a GS catalytic domain (Gln-synt_C) (Figure 2A). The phylogenetic tree of TsGS with GS of other *Trichinella* species/genotypes is shown in Figure 2B. The phylogenetic tree showed that a monophyletic group of the genus *Trichinella* was well supported. Within the genus *Trichinella*, two clear clades were present: one was the clade of eight encapsulated species/gene types (*T. spiralis*, T9, *T. nativa*, *T. britovi*, *T. patagoniensis*, *T. murrelli*, T8, and T6), and the other was the clade of three non-encapsulated species (*T. pseudospiralis*, *T. papuae*, and *T. zimbabwensis*).

### Cloning, Expression, and the Antigenicity of rTsGS

**FIGURE 3:**
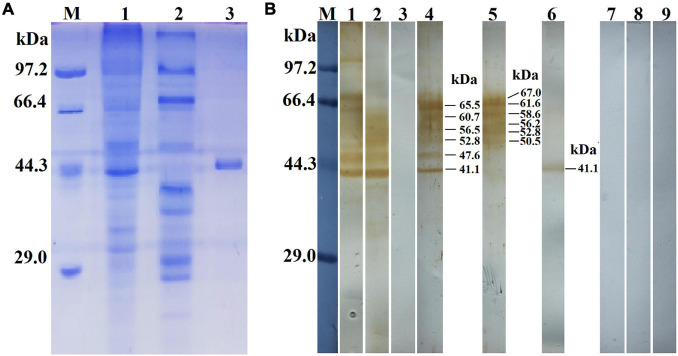
Identification of rTsGS antigenicity. **(A)** SDS-PAGE analysis of rTsGS. Lane M, protein marker; lane 1, intestinal infectious larvae (IIL) crude proteins; lane 2, IIL ES proteins; lane 3, rTsGS. **(B)** Western blotting of rTsGS antigenicity. IIL crude proteins (lane 1) and IIL ES proteins (lane 2) were recognized by infection serum, but rTsGS (lane 3) was not recognized by infection serum. Native TsGS in IIL crude proteins (lane 4) and IIL ES proteins (lane 5) and rTsGS (lane 6) were identified by anti-rTsGS serum. IIL crude proteins (lane 7), IIL ES proteins (lane 8), and rTsGS (lane 9) were not recognized by normal mouse serum.

The results of the SDS-PAGE revealed that the MW of the rTsGS expressed by BL21 bacteria carrying pET-28a/TsGS was 41.1 kDa, which was consistent with the predicted TsGS size (Figure 3A). On Western blot analysis, rTsGS was recognized by anti-rTsGS serum, but not by infection serum and normal serum (Figure 3B). Moreover, natural TsGS with 41.1–67 kDa in 6-h IIL soluble and ES proteins was identified by anti-rTsGS serum, suggesting that TsGS was a worm somatic and excretory/secretory protein.

### Transcription and Expression of TsGS in Diverse Worm Phases

**FIGURE 4:**
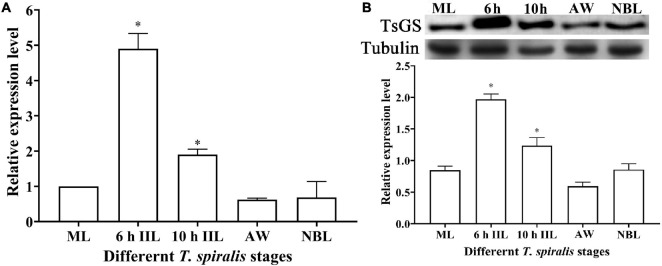
Transcription and expression of TsGS in various *Trichinella spiralis* stages. **(A)** qPCR analysis of TsGS transcription in various *T. spiralis* stages. **(B)** Western blotting of TsGS expression in somatic proteins of muscle larvae (ML), 6-h intestinal infectious larvae (IIL), 10-h IIL, 3-day adult worms (AW), and newborn larvae (NBL). The TsGS expression level in IIL stages was significantly higher than in other stages. ^∗^*P* < 0.05 compared with the ML group.

The qPCR results showed that the TsGS transcription level in 6- and 10-h IIL stages was statistically higher than in other worm stages (ML, AW, and NBL) (*F* = 116.073, *P* < 0.05) (Figure 4A). On Western blotting with anti-rTsGS serum, the natural TsGS expression level in 6- and 10-h IIL was also significantly higher than in the other three worm stages (ML, AW, and NBL) (*F* = 108.254, *P* < 0.01) (Figure 4B), suggesting that TsGS might be a molting-related protease of the IIL stages.

### Expression and Worm Tissue Location of Native TsGS in Diverse *Trichinella spiralis* Stage

**FIGURE 5:**
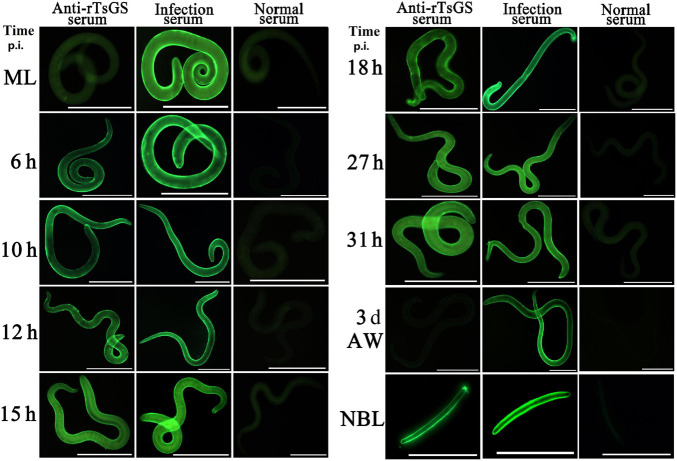
Expression of TsGS on the surface of various *Trichinella spiralis* stages by IIFA with anti-rTsGS serum. The IIFA was performed with whole intact worms of different *T. spiralis* stages [muscle larvae (ML), 6–31-h intestinal infectious larvae (IIL), adult worms (AW), and newborn larvae (NBL)]. The worms were blocked with 5% normal goat serum at 37°C for 2 h and then probed at 37°C for 2 h with 1:10 diluted diverse sera (anti-rTsGS serum, infection serum, and normal serum). After washes with phosphate-buffered saline, the worms were stained using 1:100 dilutions of goat anti-mouse IgG-FITC conjugate and examined under fluorescence microscopy. Intense fluorescence staining was observed on the epidermis of IIL, AW, and NBL by using anti-rTsGS serum, but not at ML and AW stages. The worms recognized by infection serum served as a positive control and normal serum as the negative control. Scale bars = 100 μm.

**FIGURE 6:**
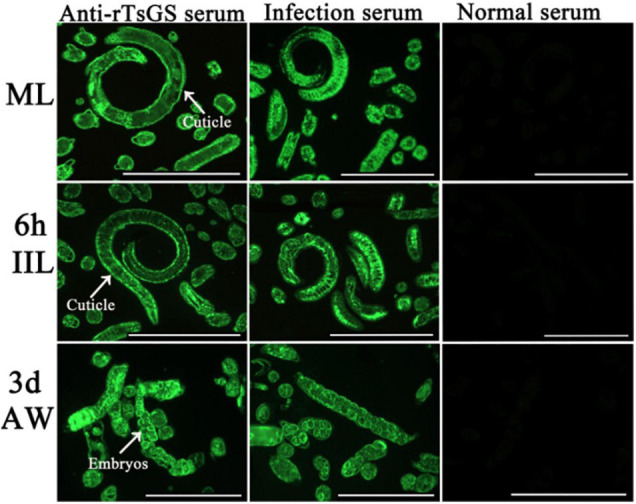
Immunolocalization of TsGS in worm cross-sections of diverse *Trichinella spiralis* stages by IIFA with anti-rTsGS serum. Fluorescence staining was observed at the cuticle and around embryos of the nematode. The worms recognized by infection serum served as a positive control and normal serum as the negative control. Scale bars = 100 μm.

The results of IIFA with intact parasites revealed that intense immunostaining on the epicuticle of 6–of-h IIL, 31-h pre-adults, and NBL was observed by using anti-rTsGS serum (Figure 5), but not on the epicuticle of ML and adult stages. When the worm cross-sections were probed by anti-rTsGS serum, the immunostaining was localized at the cuticle of ML and IIL and around embryos in the female uterus of this nematode (Figure 6).

### Enzyme Activity of rTsGS

**FIGURE 7:**
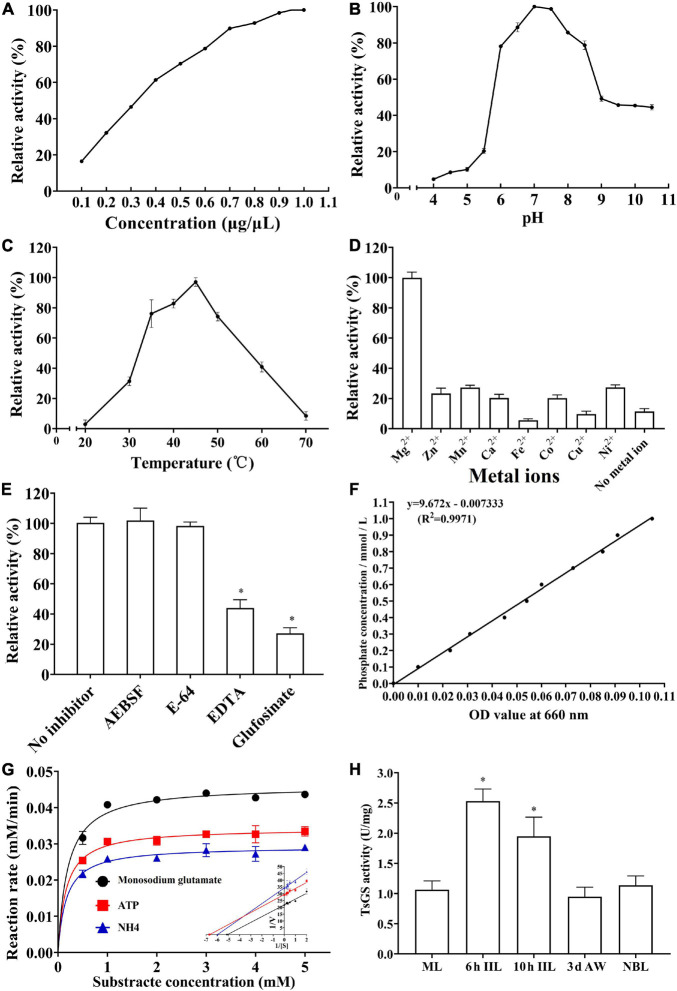
Enzymatic activities of rTsGS. rTsGS was first re-naturated by dilution refolding method. The diluted rTsGS (0.05–1.00 μg/μl) was incubated at 35°C for 15 min in various pH buffer (pH 3.0–10.5); subsequently, the glutamine synthetase substrates (Glu, ATP, and NH_4_^+^) were added to the reaction mixture and incubated at various temperatures (20–70°C) for 30 min, and the absorbance at 660 nm was measured with a spectrophotometer. **(A)** rTsGS enzyme activity at different rTsGS concentrations; the optimal concentration was 0.95 μg/μl. **(B)** rTsGS activity at different pH values; the optimal pH was 7.0. **(C)** rTsGS activity at different temperatures; the optimal temperature was 45°C. **(D)** Effects of different metal ions on rTsGS activity; the rTsGS activity is dependent on Mg^2+^. **(E)** Suppression of different inhibitors on rTsGS activity. *P < 0.01 compared to the no inhibitor, AEBSF and E-64 groups. **(F)** Standard curve of phosphorus. **(G)** Michaelis–Menten curve and Lineweaver–Burk at pH 7.0, 45°C, and 100 mM Mg^2+^. **(H)** TsGS enzyme activity of various *Trichinella spiralis* stages. *P < 0.01 compared to other three worm stages.

As shown in Figure 7A, the rTsGS enzyme activity increased with an elevation of rTsGS concentration and tended to be stable when the rTsGS concentration was 0.95 μg/μl. The optimal pH value for rTsGS activity is 7.0, and the optimal temperature is 45°C (Figures 7B,C). Mg^2+^ is necessary for the rTsGS enzymatic activity (Figure 7D). Both EDTA and glufosinate could inhibit the rTsGS enzyme activity (Figure 7E). The hydrolytic roles of rTsGS on three substrates (Glu, ATP, and NH_4_^+^) obeyed simple Michaelise–Menten kinetics, and the kinetic parameters of different zymolytes were *V*_max_ 0.05 μM min^–1^ and Km 0.20 mM of Glu, *V*_max_ 0.03 μM min^–1^ and Km 0.16 mM of ATP, and *V*_max_ 0.03 μM min^–1^ and Km 0.17 mM of NH_4_^+^, respectively (Figures 7F,G).

The enzymatic activity of natural TsGS in crude proteins of various worm stages was also determined. The results showed that the TsGS activity in 6- and 10-h IIL was significantly higher than in other worm stages (ML, AW, and NBL) (*F* = 33.665, *P* < 0.01) (Figure 7H).

### Delivery of siRNA Into *Trichinella spiralis* Muscle Larvae

**FIGURE 8:**
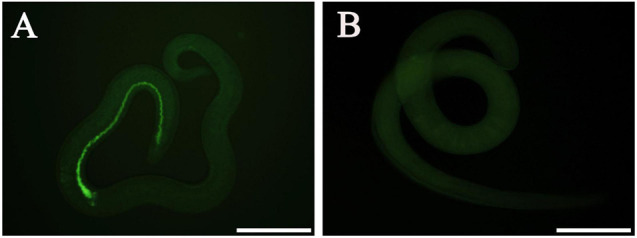
Introduction of control siRNA by electroporation. **(A)** Introduction of FAM-labeled siRNA into the muscle larvae at 12 h after electroporation. **(B)** No green fluorescence was observed in the untreated larvae. Scale bars = 100 μm.

At 12 h after electroporation with control siRNA-labeled FAM, green fluorescence staining was observed in the ML gut under fluorescence microscopy, but no fluorescence staining was found in the untreated ML (Figure 8), indicating that siRNA was successfully introduced into the ML by the electroporation technique. After the transfected ML were cultivated for 3 days, the larval mortality of siRNA-356, control siRNA, the inhibitor glufosinate, and the PBS group was 3.74, 2.95, 3.28, and 3.14%, respectively, (*F* = 0.487, *P* > 0.05), suggesting that siRNA had no obvious effect on larval survival.

### Reduction of TsGS Expression and Activity After Silencing the TsGS Gene

**FIGURE 9:**
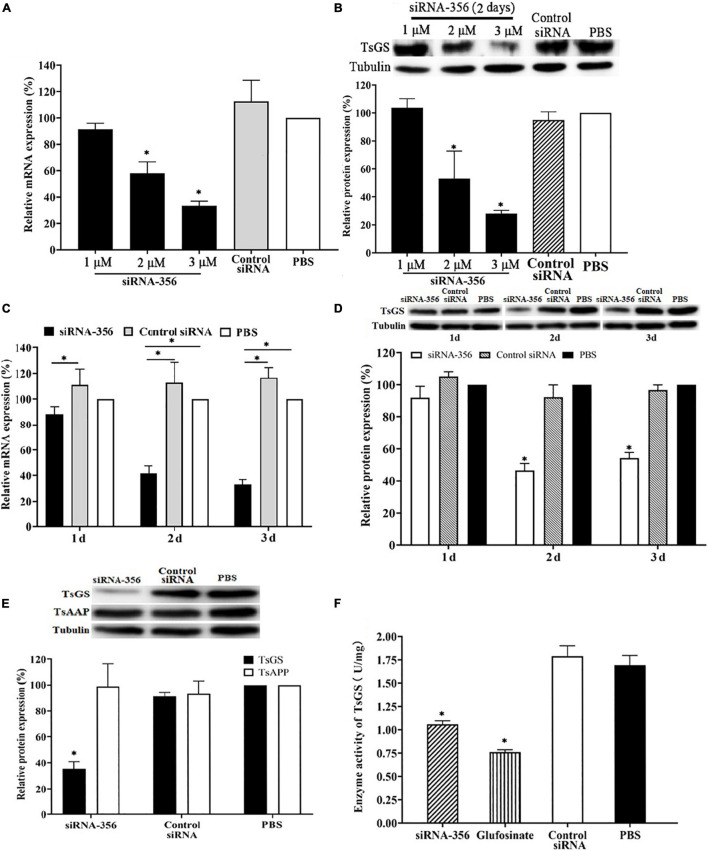
Silencing of TsGS gene reduced the TsGS expression and enzyme activity. **(A)** Effect of various doses of siRNA-356 on TsGS transcription level. **(B)** Effects of various doses of siRNA-356 on TsGS protein expression. **(C)** TsGS transcription level at 1–3 days after treatment with 3 μM siRNA-356. **(D)** TsGS expression level at 1–3 days after treatment with 3 μM siRNA-356. **(E)** Expression levels of TsGS and *Trichinella spiralis* aspartyl aminopeptidase in muscle larvae (ML) treated using TsGS-specific siRNA-356. **(F)** Native TsGS enzyme activity to hydrolyze its substrate was significantly declined in siRNA-356 and inhibitor- treated ML.^∗^*P* < 0.05 compared to the phosphate-buffered saline group.

Compared with control siRNA and the PBS group, TsGS mRNA and the protein expression level in ML treated with 3 μM siRNA-356 were reduced by 58.48 and 71.89%, respectively (*P* < 0.01) (Figures 9A,B). After being treated with 3 μM siRNA-356 for 3 days, TsGS mRNA and the protein expression level were decreased by 66.67 and 54.49% (*P* < 0.05) (Figures 9C,D). However, the TsGS expression levels were not reduced when the ML were treated using control siRNA. Additionally, when a *T. spiralis* aspartyl aminopeptidase gene (TsAAP, GenBank: KRY29491.1) was used as a control, the TsAAP expression was not suppressed in ML treated with TsGS-specific siRNA-356 (Figure 9E), suggesting that siRNA-356 is TsGS specific. The results of the enzymatic activity assay showed that the natural TsGS enzyme activity in the somatic soluble proteins of siRNA-transfected ML was decreased by 37.42% compared with the non-treated ML (*P* < 0.05) (Figure 9F).

### Effects of Acidic Conditions on Larval Survival and TsGS Activity

**FIGURE 10:**
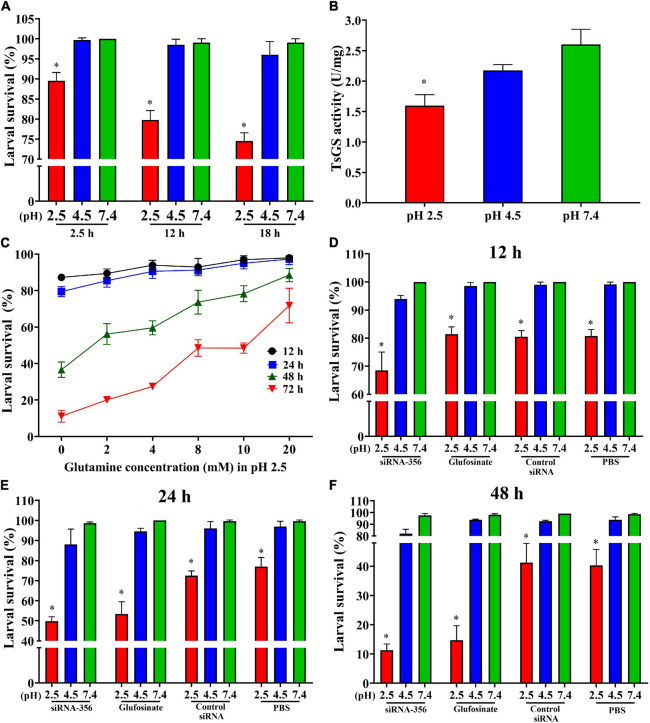
Acid-resistant tests of *Trichinella spiralis* muscle larvae. The effect of various acidic conditions on the survival of *T. spirali*s muscle larvae (ML) was assessed. One hundred ML were cultured at pH 2.5, 4.5, and 7.4 at 37°C and 5% CO_2_ for 2.5, 12, and 18 h. The survival of the ML cultured in phosphate-buffered saline with various pH values was examined under a microscope on the basis of larval viability. Inactive, straight, or “C”-shaped larvae were assessed as dead larvae. The mobile and wriggly larvae were counted as live larvae. **(A)** Effect of different pH on ML survival in various culture times. **(B)** TsGS enzyme activity of the ML cultured at pH 2.5 for 18 h. **(C)** Effect of different glutamine concentrations on the survival of ML at pH 2.5. **(D–F)** Silencing of TsGS gene was reduced on larval survival at pH 2.5 for 12 **(D)**, 24 **(E)**, and 48 h **(F)**. *P < 0.05 compared to the pH 7.4 group.

The results of the ML acid resistance assay showed that the larval survival of the ML cultured at pH 2.5 for 2.5, 12, and 18 h was evidently lower than the ML cultured at pH 7.4 (χ^2^_2_._5 *h*_ = 10.526, *P* < 0.05; χ^2^_12 *h*_ = 19.207, χ^2^_18 *h*_ = 25.464, *P* < 0.0001) (Figure 10A). After culturing for 18 h, the enzymatic activity of native TsGS in crude proteins of the ML at pH 2.5 was statistically lower than that at pH 4.5 and 7.4 (*F* = 22.222, *P* < 0.05) (Figure 10B). When the ML was cultured at pH 2.5 for 48 and 72 h, a supplement of glutamine in the medium obviously increased the larval survival; the larval survival rate was elevated with the increase of glutamine doses (*r*_48 *h*_ = 0.891, *r*_72 *h*_ = 0.969, *P* < 0.05) (Figure 10C), suggesting that the ML acid resistance was dependent on glutamine dose under *in vitro* culture condition.

To evaluate the effects of silencing the TsGS gene on the ML acid resistance, the survival of the siRNA-356-treated ML was also observed. The result revealed that, after being cultured at pH 2.5 for 24 and 48 h, the survival of siRNA-356-treated ML declined by 35.32 and 71.97% compared to the PBS group (χ^2^_24 *h*_ = 15.726, χ^2^_48 *h*_ = 22.134, *P* < 0.0001) (Figures 10D–F). When siRNA-356-treated ML were cultured at pH 2.5 for 12, 24, and 48 h, larval survival was declined by 31.48, 49.45, and 88.41% compared to the pH 7.4 group (χ^2^_12 *h*_ = 36.686, χ^2^_24 *h*_ = 63.139, χ^2^_48 *h*_ = 152.616, *P* < 0.0001), suggesting that TsGS is involved in ML acid resistance.

### siRNA-365 Impeded Larval Infectivity, Molting, Development, and Fecundity

**FIGURE 11:**
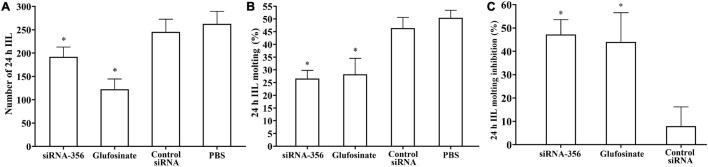
Suppression of siRNA-356 on larval infectivity and molting. **(A)** Intestinal worm burden of the intestinal infectious larvae (IIL) recovered at 24 h post-infection (*n* = 10). **(B)** Molting rate of 24-h IIL (*n* = 50). **(C)** siRNA-356 inhibited IIL molting. ^∗^*P* < 0.05 compared to the control siRNA and phosphate-buffered saline group.

**FIGURE 12:**
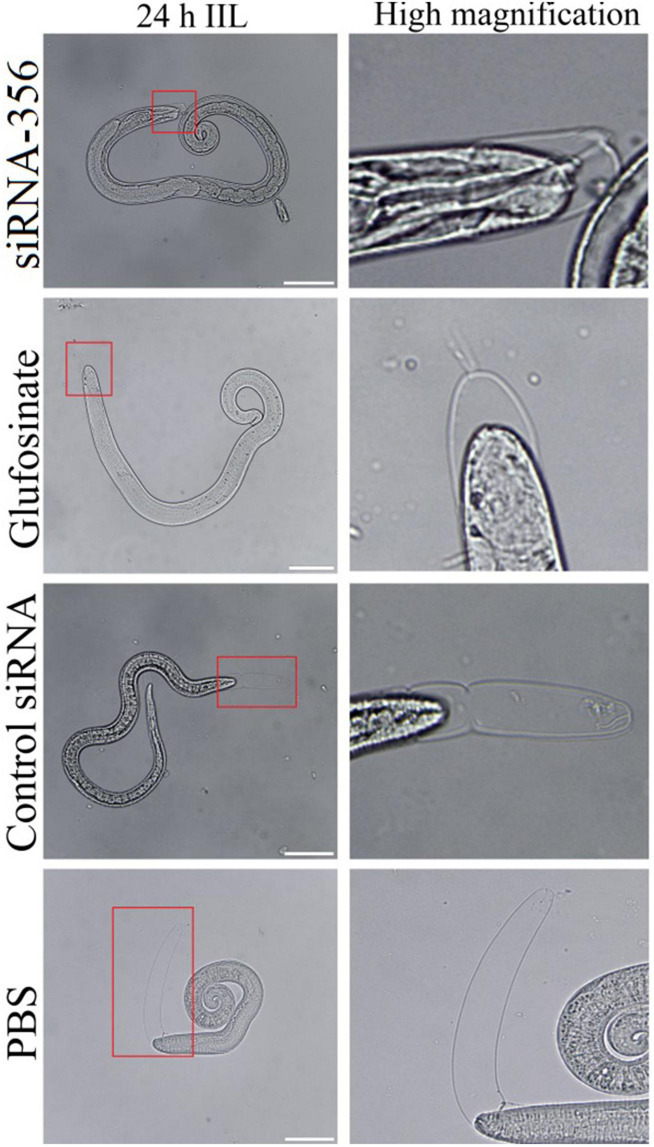
The larval molting was suppressed by siRNA-356. In the 24-h intestinal infectious larvae (IIL) stage, the obvious suppression of larval molting was also observed in siRNA-356 and inhibitor glufosinate group. The area in the red box was magnified for observation. Scale bars = 50 μm.

At 24 h after a challenge infection, the number of 24-h IIL recovered from infected mouse intestine in the siRNA-356 treatment group were reduced by 26.98% compared to the control siRNA and PBS group (*F* = 67.220, *P* < 0.0001) (Figure 11A). The molting percentage of siRNA-365, control siRNA, inhibitor, and PBS group were 26.61, 46.41, 28.22, and 50.43%, respectively (*F* = 39.452, *P* < 0.0001) (Figure 11B). Larval molting of the siRNA-365 group was suppressed by 47.24% compared to the PBS group (*F* = 27.017, *P* < 0.0001) (Figures 11C, 12).

**FIGURE 13:**
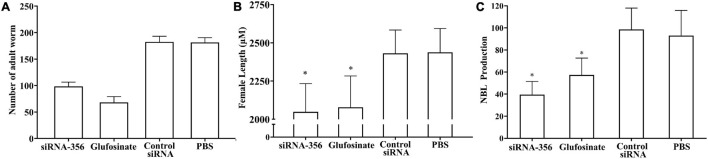
Intestinal adult burden, female length, and fecundity of mice infected with muscle larvae transfected with siRNA-356. **(A)** Intestinal adult burdens (*n* = 10). **(B)** Female adult length (*n* = 30). **(C)** Female fecundity (*n* = 10). ^∗^*P* < 0.0001 compared with the control siRNA and phosphate-buffered saline group.

The mice challenged with siRNA-356-transfected ML showed 45.70% reduction of enteral AW at 6 dpi compared with the PBS group (*F* = 358.483, *P* < 0.001) (Figure 13A). The length of female adults of the siRNA-356 group was reduced by 25.58% relative to the control siRNA and PBS groups (*F* = 27.061, *P* < 0.001) (Figure 13B), but the male length was not obviously changed among the four groups (*P* > 0.05). The female reproduction (NBL production by each adult female) from the siRNA-365 group was prominently inferior to those of the control siRNA and PBS group (*F* = 27.645, *P* < 0.0001) (Figure 13C). Additionally, glutamine synthetase-specific inhibitor glufosinate also significantly mitigated the enteral worm burdens of IIL and AW, impaired larval molting, and reduced female fecundity. The results demonstrated that silencing of the TsGS gene or inhibition of TsGS activity significantly impaired larval molting, development, and female reproduction.

## Discussion

Glutamine synthetase participates in many biological processes and plays an extremely important role in parasites, which show metabolic characteristics to help parasites adapt to different environments in their life cycle and make use of host resources. The multiple antigens containing *Leishmania chagasi* GS may be optimal for protective vaccines (Martins et al., 2006). In *Leishmania mexicana* parasites, inhibition of the mitochondrial tricarboxylic acid circulation or GS strongly inhibited the growth and development of amastigotes *in vitro* (Saunders et al., 2014). In *T. cruzi*, GS is obligatory to resist ammonium accumulation toxicity and immune escape during host cell invasion (Crispim et al., 2018).

In the present study, TsGS has a GS catalytic domain (Gln-synt_C). The complete TsGS cDNA sequence was cloned into a pET-28a expression vector, and rTsGS was expressed in an *E. coli* expression system. Sequence analysis showed that TsGS had 92–99% identity with the GS of other species/gene types from the genus *Trichinella*, suggested that the sequences of GS are highly conserved in the genus *Trichinella*. Following being induced and purified, rTsGS was used to subcutaneously immunize mice and to produce anti-rTsGS serum. The anti-rTsGS IgG titer was 10^4^ at 2 weeks after the final immunization, suggesting that rTsGS has good immunogenicity. On Western blot analysis, rTsGS was recognized by anti-rTsGS serum, but not by infection serum and normal serum. Moreover, as shown in Figure 3B, the natural TsGS in 6-h IIL soluble and ES proteins was identified by anti-rTsGS serum, suggesting that TsGS was a worm somatic and ES protein. Additionally, rTsGS was not recognized by infection serum on Western blotting; it is likely because the TsGS is a molt-related protein which is highly expressed only at the epicuticle of IIL during molting and not at epicuticle of ML and AW stages. The TsGS expressed by IIL in a short time could not stimulate the host to generate a high level of anti-TsGS antibodies during natural *T. spiralis* infection (Li et al., 2015).

The results of qPCR and Western blotting revealed that TsGS was transcribed and expressed in various *T. spiralis* stages (ML, 6- and 10-h IIL, 3-day-old AWs, and NBL), and the expression levels of TsGS mRNA and protein at 6- and 10-h IIL stages were evidently higher than those of other worm stages. After being ingested, the ML are activated into the intestinal first-stage infectious larvae (IIL1) in gut at 0.9 hpi. The IIL1 intrude the intestinal epithelium of the host and undergo the first molting to develop IIL2 prior to 10 hpi, then IIL2 molt three times to adulthood at 10–14, 15–22, and 23–30 hpi, respectively (Liu et al., 2013). In order to observe the expression of natural TsGS at various IIL stages, the 6-, 10-, 12-, 15-, 18-, 27-, and 31-h IIL were used in the IIFA test. The results of IIFA with intact parasites showed that native TsGS was identified on the surface of IIL and NBL, but not on that of ML and AW stages. By using IIFA with parasite cross-sections, native TsGS was mainly localized at the cuticle and intrauterine embryos of the parasite. The results suggested that TsGS was closely related to the molting, growth, and development of the IIL (Ren et al., 2019). In addition, native TsGS with 41.1–67 kDa in IIL crude proteins and ES proteins was recognized by anti-rTsGS serum; it is likely because TsGS has various isoforms. The TsGS protein might be processed by post-translational processing and modification, or it is due to the native TsGS probed by polyclonal anti-rTsGS serum (Bien et al., 2015; Cui et al., 2015b; Sun et al., 2018; Hu et al., 2020).

The enzymatic activity test showed that rTsGS had the enzymatic activity of native GS to hydrolyze its specific substrate (Glu, ATP, and NH_4_^+^). The optimal temperature and pH value of rTsGS *in vitro* were 45°C and pH 7.0, which is similar with that of *Schistosoma japonicum* GS (Qiu et al., 2012). Acid resistance is the response of *Trichinella* ML to the pH change of the external environment. TsGS plays an important role in the process of glutamine synthesis within the ML worm body. Only when the ML are in acidic conditions of the host stomach do ML initiate the AR4 system to adapt to the acidic environment. The whole process of ML acid resistance is developed within the worm body, not in the external condition. Hence, rTsGS is inactive at acidic conditions in the external environment. The rTsGS activity was obviously enhanced by Mg^2+^, and Mg^2+^ was a TsGS-dependent metal ion. Similar to other GS, rTsGS was sensitive to glufosinate and EDTA (Crispim et al., 2018). The TsGS activity in IIL stage was significantly higher than in other worm stages (as shown in Figure 7H). It is likely because TsGS is involved in the molting, growth, and development of *T. spiralis* IIL in the host intestine. GS catalytically condenses glutamine with glutamate in the presence of ATP, NH_4_^+^, and Mg^2+^ to form glutamine, which is a carbon and nitrogen donor for the production of biomolecules and participates in redox dynamic equilibrium (Lai et al., 2011; Wang et al., 2014). After invading into the IECs, the IIL develops into adults after molting four times. Molting is a key step for parasitic nematodes to enlarge their body size and adapt to the environment, while glutamine synthesized by GS is involved in nucleotides, amino acid synthesis, and energy metabolism in the mitochondria, which is important for larval growth and development. TsGS is involved in the synthesis of glutamine, which provides nitrogen for the production of purines, pyrimidines, amino acids, and other compounds needed for complex cellular functions.

The survival of any organisms must depend on their ability to maintain an appropriate range of pH values. It is known that physiological and biological buffering systems mainly rely on ammonia and carbon dioxide (Krulwich et al., 2011), which are responsible for the neutralization of acids (protons) and bases (hydroxide ions), respectively. The pathogenicity of foodborne pathogens determines the inevitability of their acid resistance. Stimulation of an acidic environment induces the expression of acid-resistant genes in pathogens, which is an important cause of disease (Djoko et al., 2017). A glutamine-dependent acid-resistant system is one of the important acid-resistant systems in *E. coli*, and it uses amino acid antiporter GadC to absorb the intracellular YbaS of Gln and catalyze the hydrolysis of Gln to Glu and NH_3_, which consumes GadC and is transported out of the cell by GadC and continues to transfer into Gln. Glutamine transport and hydrolysis can help *E. coli* maintain acid–base balance (Kowalczyk et al., 2011; Wu et al., 2013). The acidic environment in the stomach of the host can also stimulate the effective expression of acid-fast genes of *T. spiralis* ML. A previous study showed that a glutaminase (TsGLS) plays an important role in the acid-resistant system of *T. spiralis* (Gao et al., 2021). As shown in Figures 10A,B, when the ML were cultured at pH 2.5 for 18 h, the larval survival was more obviously decreased at pH 2.5 than that at pH 4.5 and 7.4, and the enzyme activity of TsGS was significantly lower than that of the ML at pH 7.4. The ML survival decrease at pH 2.5 is likely because the decreased enzyme activity of TsGS led to the reduction of glutamine synthesis, which impeded the activation of the AR4 system in ML. Therefore, partial ML were not adaptable to an acidic environment and died. Our results indicated that GS is also involved in *T. spiralis* ML acid resistance, and the supplement of glutamine in culture medium obviously increased the larval survival at pH 2.5 acid environments. Glutamine is a key amino acid in biosynthesis, and it has the basic functions of providing amino acids, lipids, nucleotides, hexosamine, and polyamines, but it also provides metabolic energy (ATP) as a multi-functional cellular signal molecule (Mates et al., 2019). In addition, glutamine is an indispensable substance for the production of glutathione, and glutathione is the most important antioxidant molecule and anabolism adaptation enzyme in cells. Moreover, silencing of the TsGS gene significantly reduced the survival of the ML at pH 2.5 compared to the normal ML at pH 2.5 and the siRNA-356-treated ML at pH 7.4. The results further indicated that TsGS participates in *T. spiralis* larval acid resistance, and glutamine is also an essential component of this AR.

As an important means of studying gene function, RNA interference technology is widely used to identify genes related to the growth and development of parasites, such as *Fasciola hepatica*, *S. japonicum*, and *Ascaris suis* (Chen et al., 2011). In the parasitic nematode *T. spiralis*, silencing of some genes evidently suppressed larval invasion of the intestinal epithelium and impeded worm development and reproduction (Han et al., 2020; Yi et al., 2020). In this study, TsGS-specific siRNA-356 was introduced into the ML by electroporation. After the ML was treated with 3 μM siRNA-356 for 3 days, the TsGS expression and activity were decreased by 66.67 and 37.42%, respectively. The results of the challenge infection showed that silencing of the TsGS gene significantly reduced the intestinal 24-h IIL burden and impaired the IIL molting. Intestinal adult burden, worm development, and female fecundity were also obviously impeded by TsGS-specific siRNA as demonstrated by shorter female adults and lower female reproduction capacity. The results demonstrated that TsGS plays an essential role for larval molting, development, and female reproduction in the life cycle of *T. spiralis* (Yang et al., 2019).

In conclusion, TsGS was highly expressed at the molting IIL stage in the *T. spiralis* life cycle and principally located at the cuticle and around embryos in the uterus of the parasite. The rTsGS had good immunogenicity and the enzymatic activity of natural GS to hydrolyze the substrate (Glu, ATP, and NH_4_^+^). Silencing of the TsGS gene significantly decreased the IIL survival at acidic condition, reduced intestinal 24-h IIL burden, and impaired the IIL molting, development, and female fecundity. The results indicated that TsGS is involved in ML acid resistance and the IIL molting, development, and reproduction at the intestinal stage of the *T. spiralis* life cycle, and it might be a candidate vaccine target against *Trichinella* molting and development.

## Data Availability Statement

The original contributions presented in the study are included in the article/Supplementary Material, further inquiries can be directed to the corresponding authors.

## Ethics Statement

The animal study was reviewed and approved by the Life Science Ethics Committee of Zhengzhou University.

## Author Contributions

ZQW and JC designed this study. TXZ, ZW, YYS, SWY, RDL, and XZ conducted the experiments. TXZ, ZQW, and JC drafted and revised the manuscript. All authors read and approved the final version of this manuscript.

## Conflict of Interest

The authors declare that the research was conducted in the absence of any commercial or financial relationships that could be construed as a potential conflict of interest.

## Publisher’s Note

All claims expressed in this article are solely those of the authors and do not necessarily represent those of their affiliated organizations, or those of the publisher, the editors and the reviewers. Any product that may be evaluated in this article, or claim that may be made by its manufacturer, is not guaranteed or endorsed by the publisher.
