# Supplementary figures and images for: Characterization of a Novel Glutamine Synthetase From Trichinella spiralis and Its Participation in Larval Acid Resistance, Molting, and Development
Source: Front Cell Dev Biol. 2021 Sep 20;9:729402. doi: 10.3389/fcell.2021.729402 (PMC8488193; doi:10.3389/fcell.2021.729402)

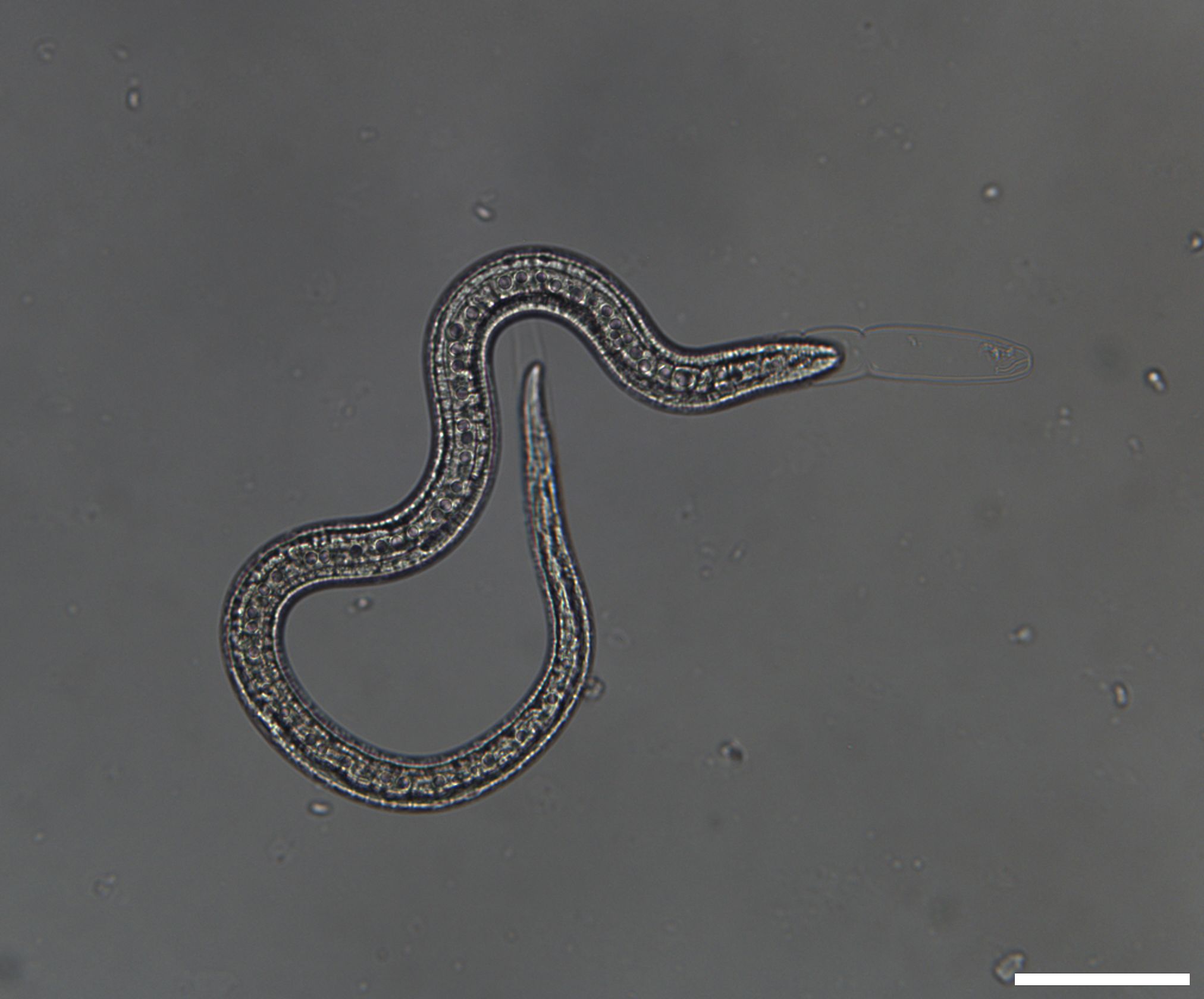

Supplement: Supplementary file 1 [file Data_Sheet_1.ZIP › 729402-supplementary material-original figures and dates-jpg-2021-7-2/729402 Fig12/Fig 12-Control siRNA.jpg]

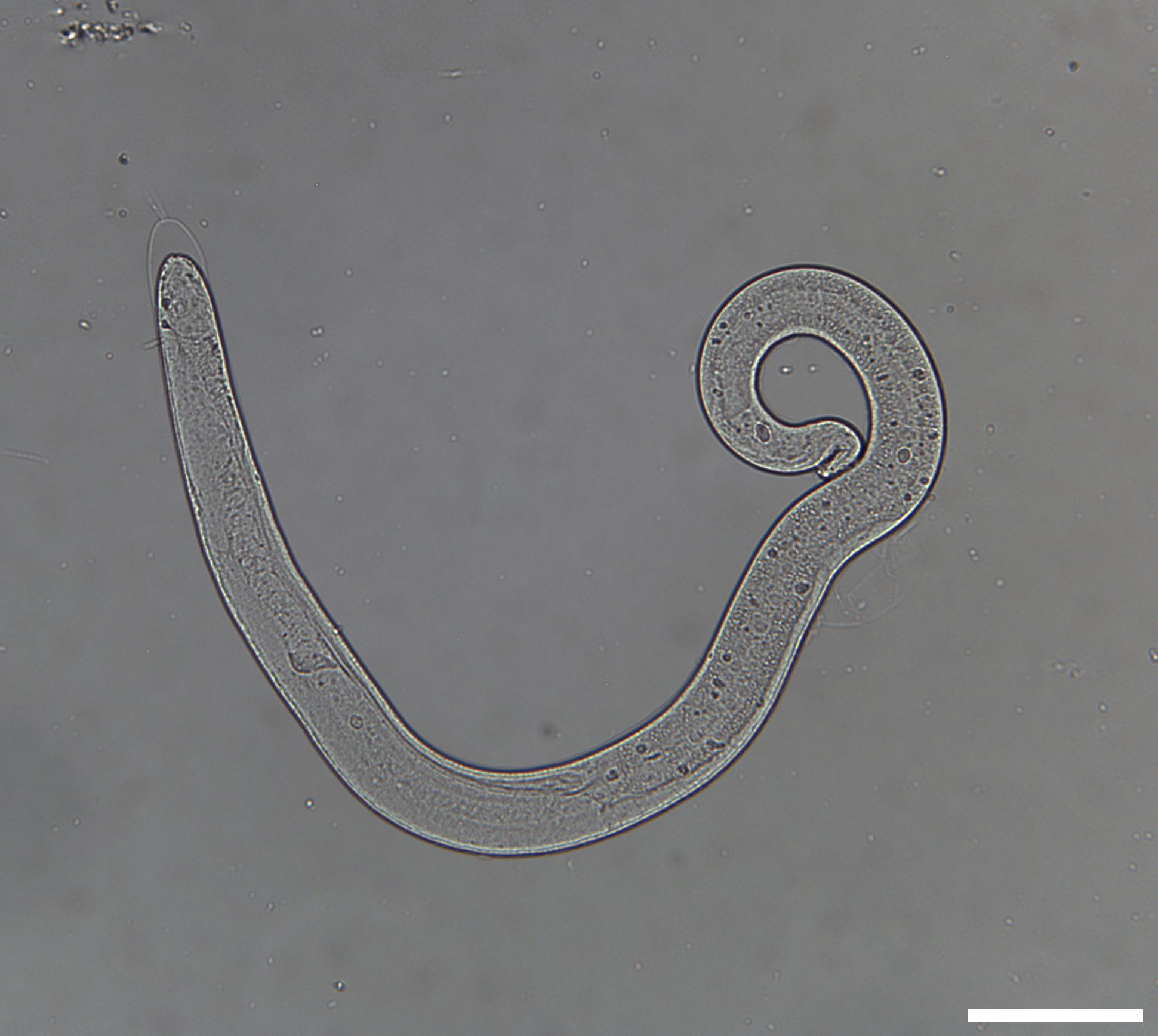

Supplement: Supplementary file 1 [file Data_Sheet_1.ZIP › 729402-supplementary material-original figures and dates-jpg-2021-7-2/729402 Fig12/Fig 12-Glufosinate.jpg]

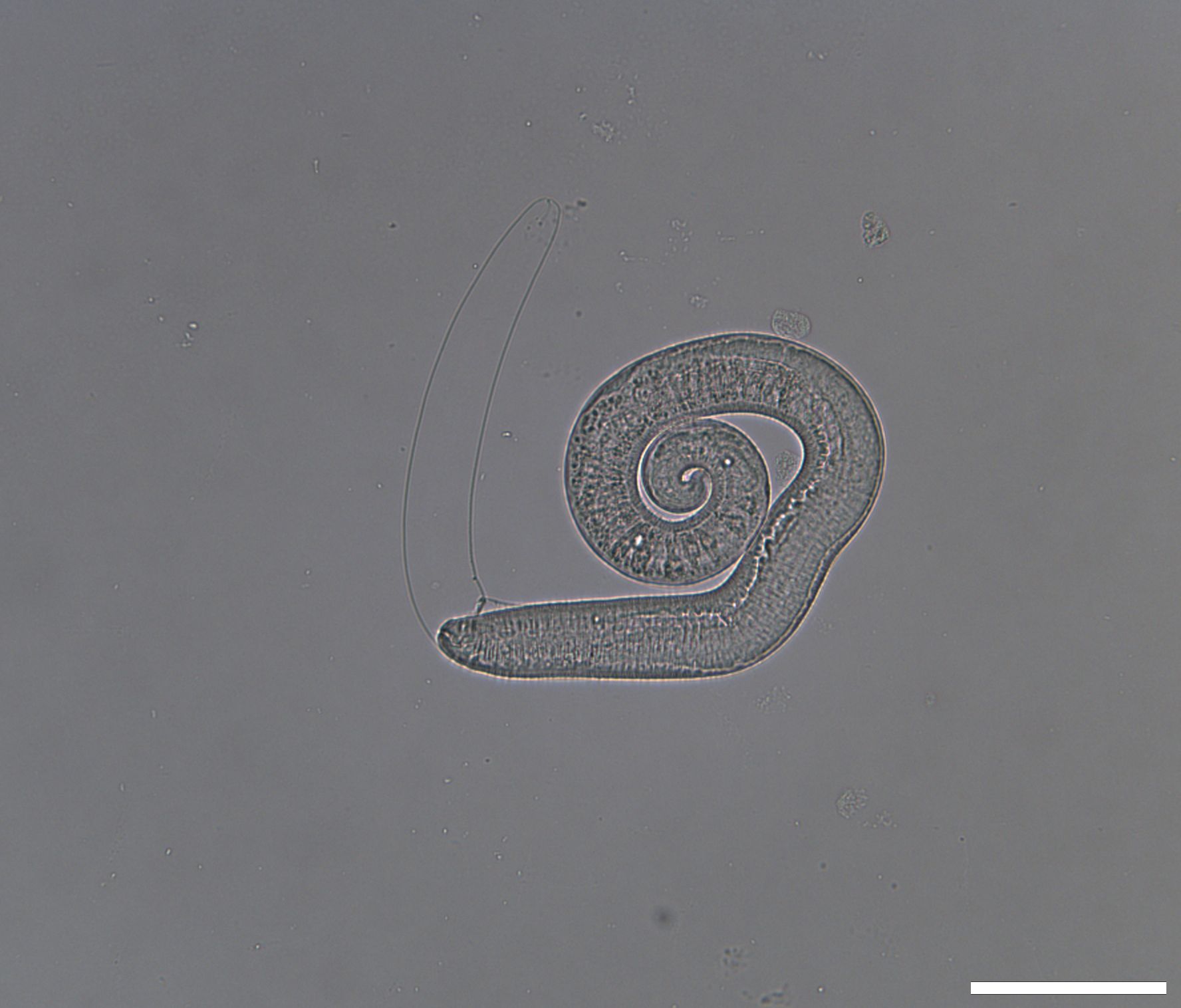

Supplement: Supplementary file 1 [file Data_Sheet_1.ZIP › 729402-supplementary material-original figures and dates-jpg-2021-7-2/729402 Fig12/Fig 12-PBS.jpg]

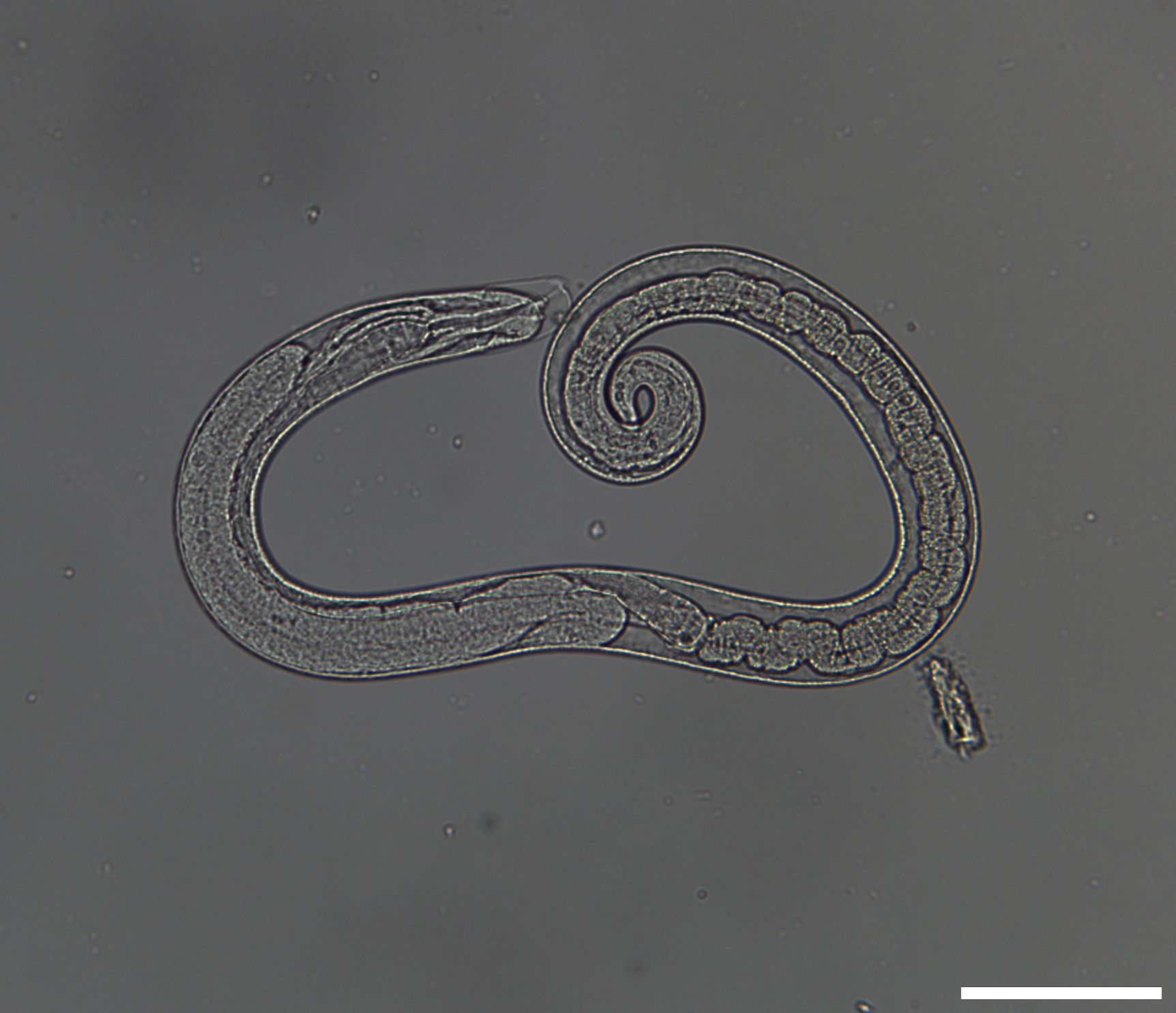

Supplement: Supplementary file 1 [file Data_Sheet_1.ZIP › 729402-supplementary material-original figures and dates-jpg-2021-7-2/729402 Fig12/Fig 12-siRNA-356.jpg]

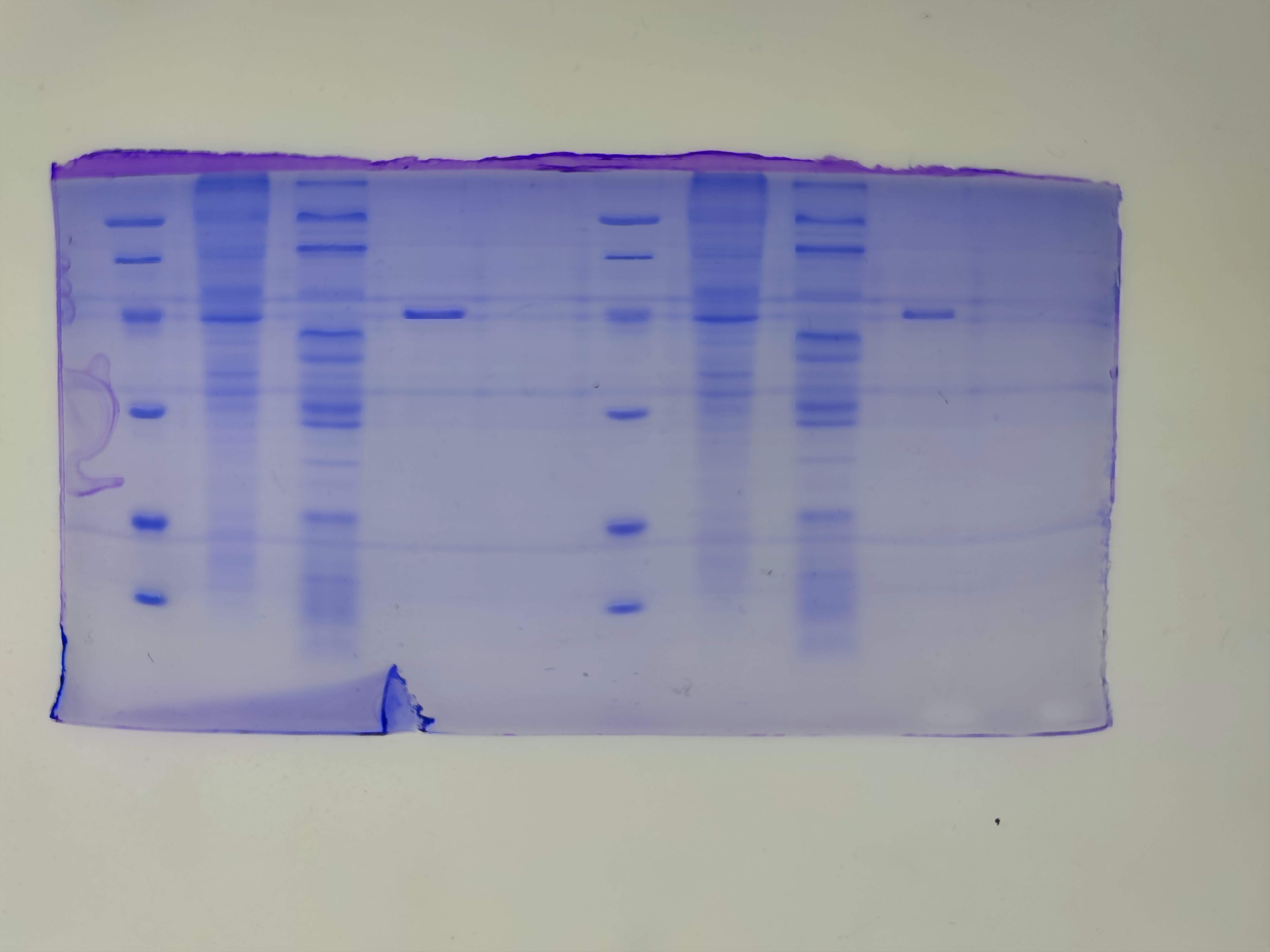

Supplement: Supplementary file 1 [file Data_Sheet_1.ZIP › 729402-supplementary material-original figures and dates-jpg-2021-7-2/729402 Fig3/Fig3-A.jpg]

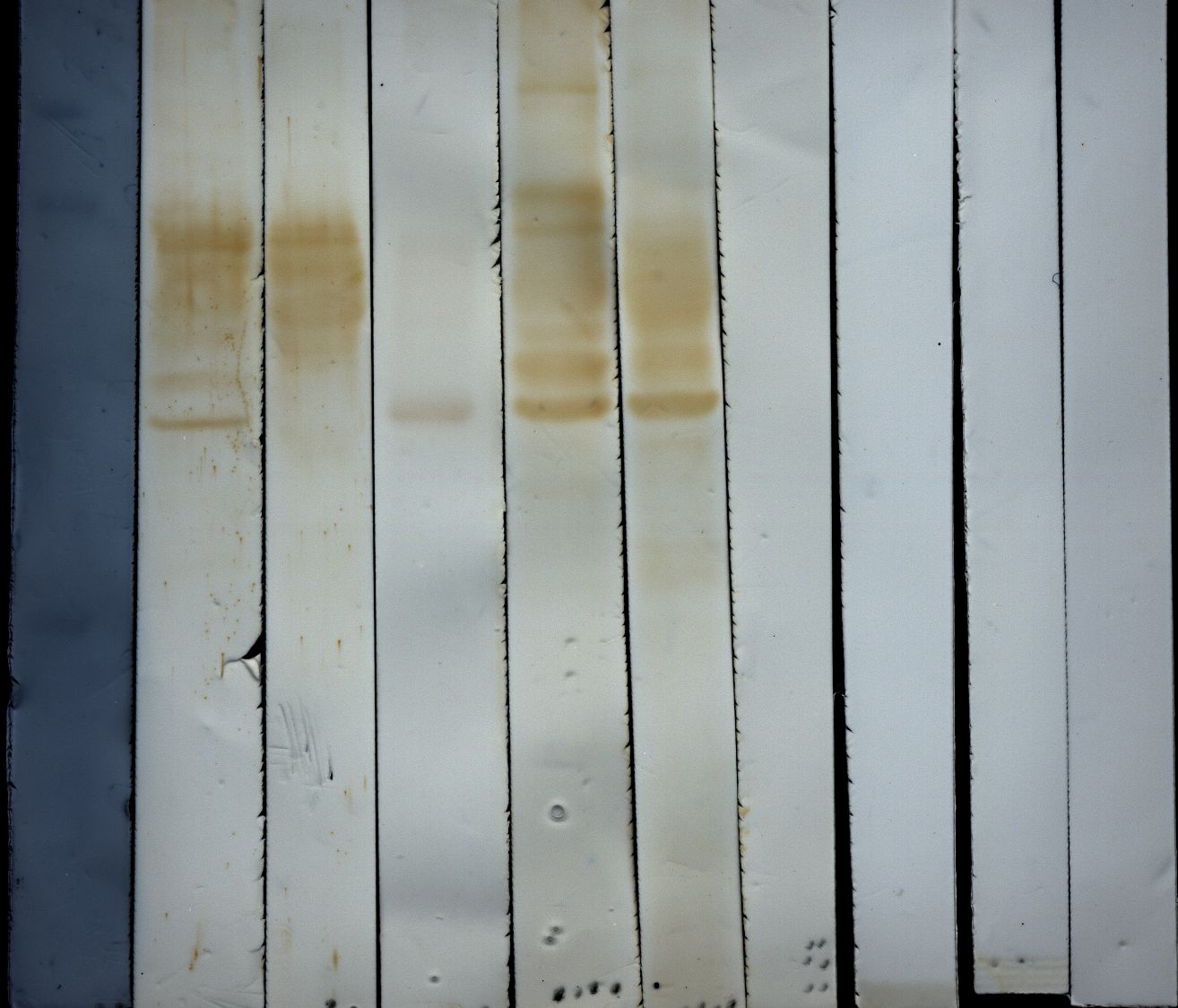

Supplement: Supplementary file 1 [file Data_Sheet_1.ZIP › 729402-supplementary material-original figures and dates-jpg-2021-7-2/729402 Fig3/Fig3-B.jpg]

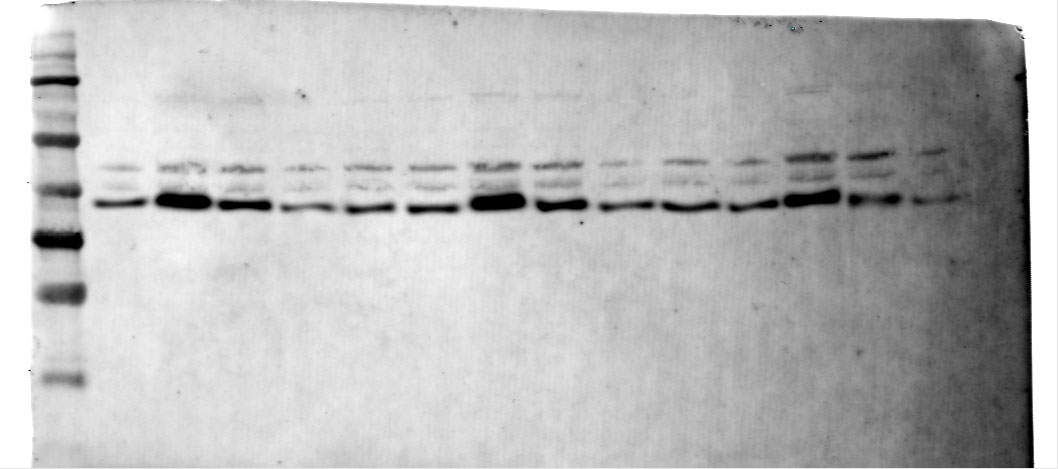

Supplement: Supplementary file 1 [file Data_Sheet_1.ZIP › 729402-supplementary material-original figures and dates-jpg-2021-7-2/729402 Fig4/Fig4-B/Fig 4B-TsGS.jpg]

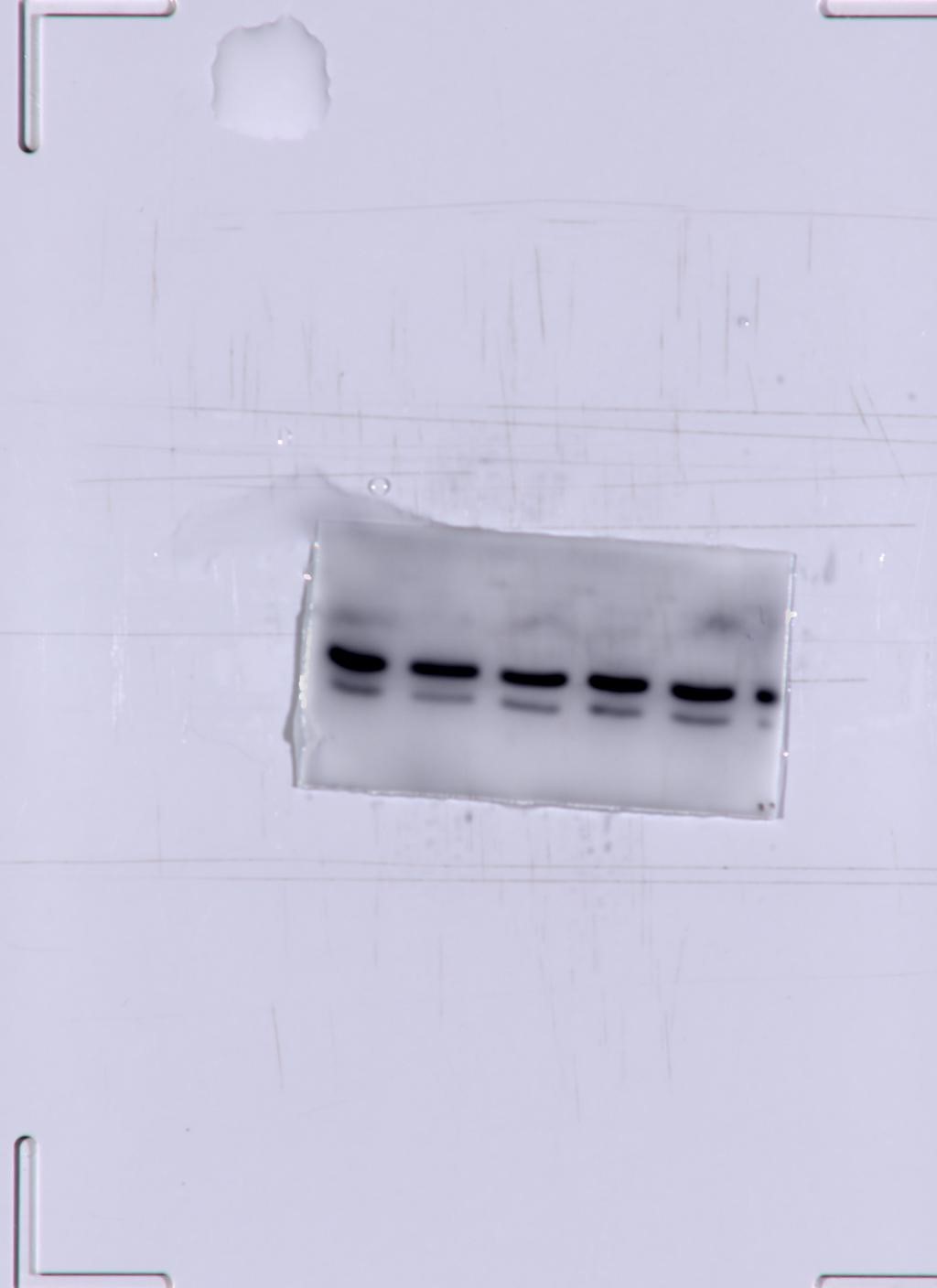

Supplement: Supplementary file 1 [file Data_Sheet_1.ZIP › 729402-supplementary material-original figures and dates-jpg-2021-7-2/729402 Fig4/Fig4-B/Fig 4B-Tubulin.jpg]

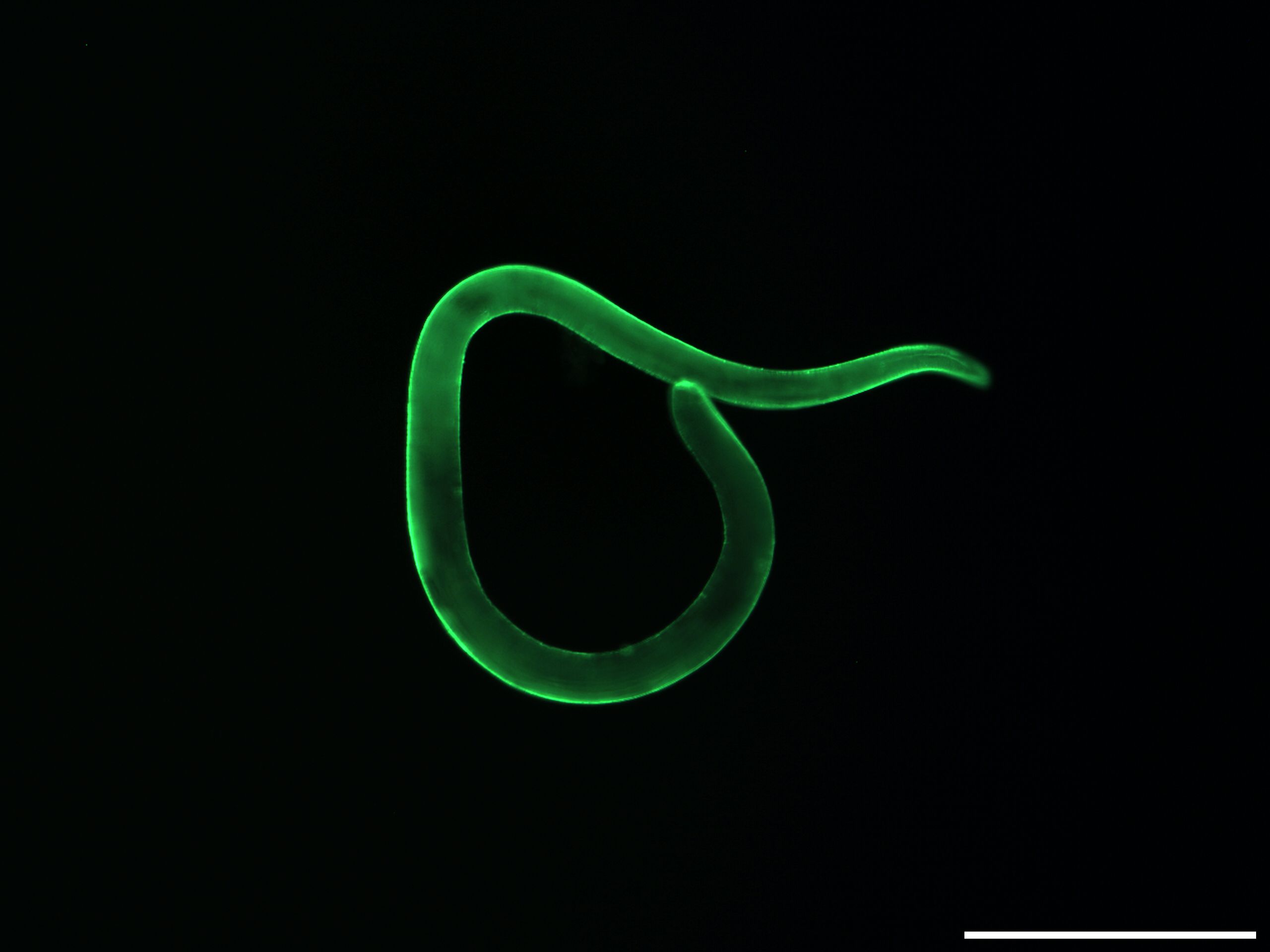

Supplement: Supplementary file 1 [file Data_Sheet_1.ZIP › 729402-supplementary material-original figures and dates-jpg-2021-7-2/729402 Fig5/Anti-rTsGS serum/Fig 5-10h IIL+Anti-rTsGS serum.jpg]

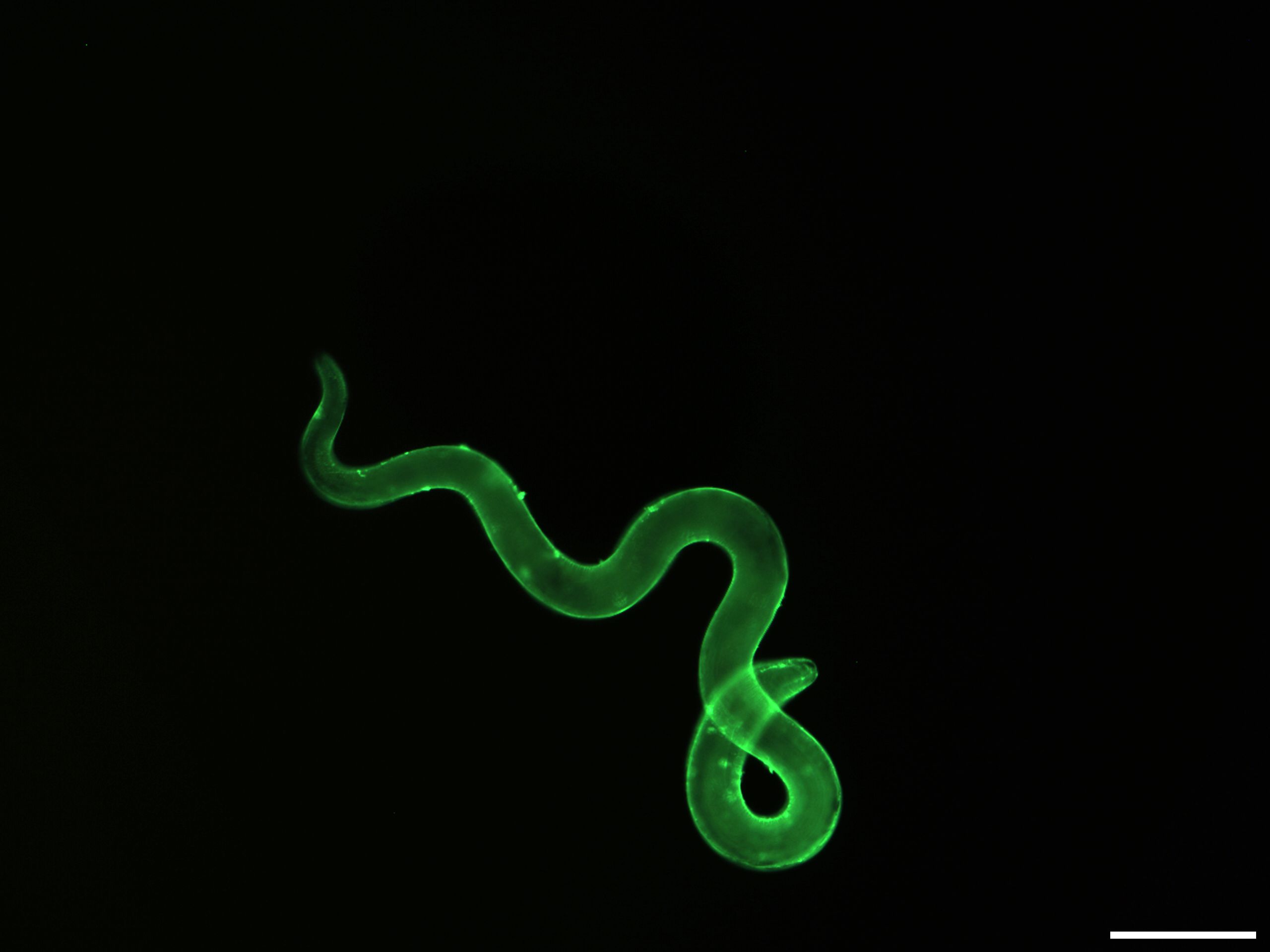

Supplement: Supplementary file 1 [file Data_Sheet_1.ZIP › 729402-supplementary material-original figures and dates-jpg-2021-7-2/729402 Fig5/Anti-rTsGS serum/Fig 5-12h+Anti-rTsGS serum.jpg]

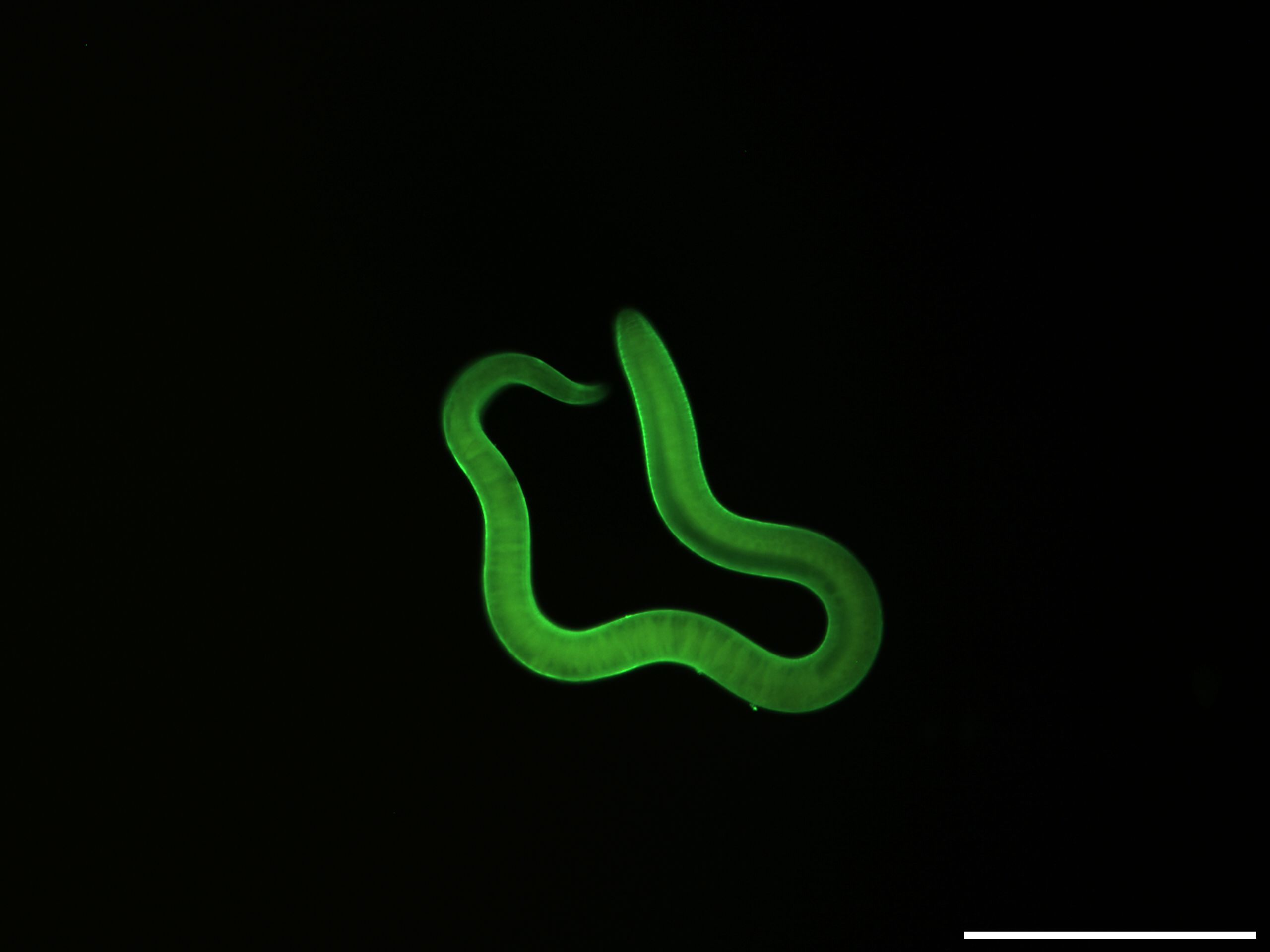

Supplement: Supplementary file 1 [file Data_Sheet_1.ZIP › 729402-supplementary material-original figures and dates-jpg-2021-7-2/729402 Fig5/Anti-rTsGS serum/Fig 5-15h+Anti-rTsGS serum.jpg]

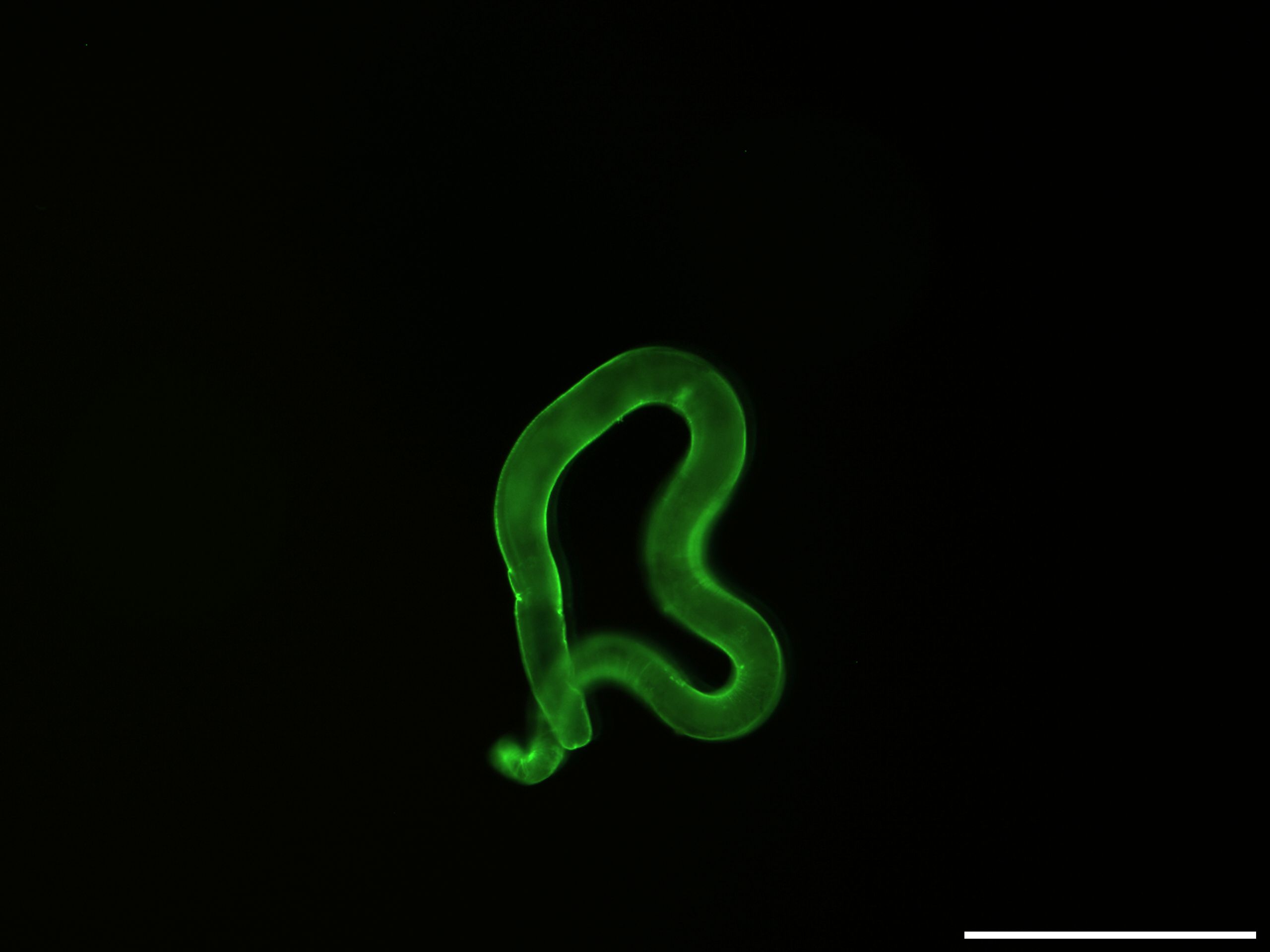

Supplement: Supplementary file 1 [file Data_Sheet_1.ZIP › 729402-supplementary material-original figures and dates-jpg-2021-7-2/729402 Fig5/Anti-rTsGS serum/Fig 5-18h+Anti-rTsGS serum.jpg]

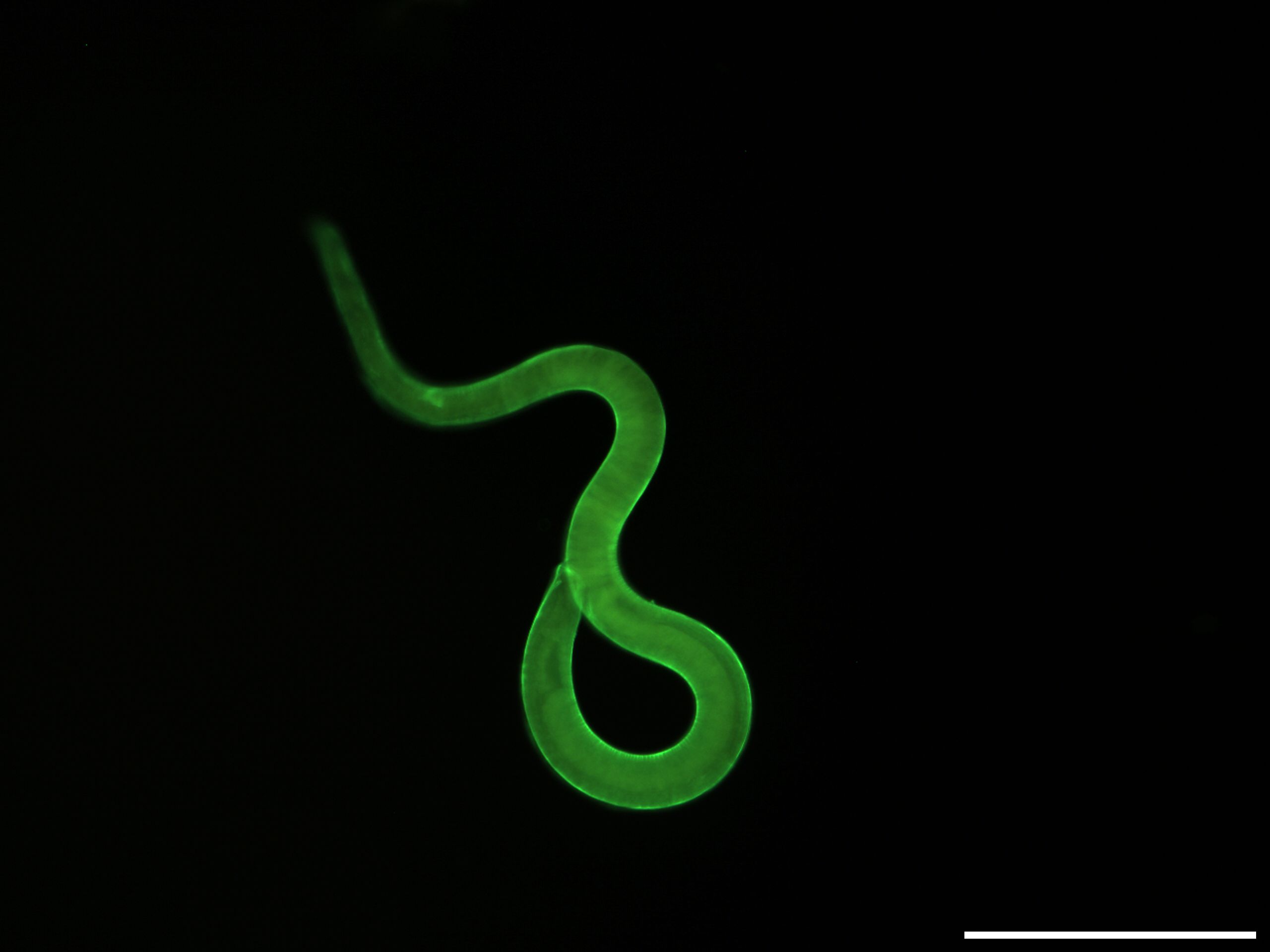

Supplement: Supplementary file 1 [file Data_Sheet_1.ZIP › 729402-supplementary material-original figures and dates-jpg-2021-7-2/729402 Fig5/Anti-rTsGS serum/Fig 5-27h+Anti-rTsGS serum.jpg]

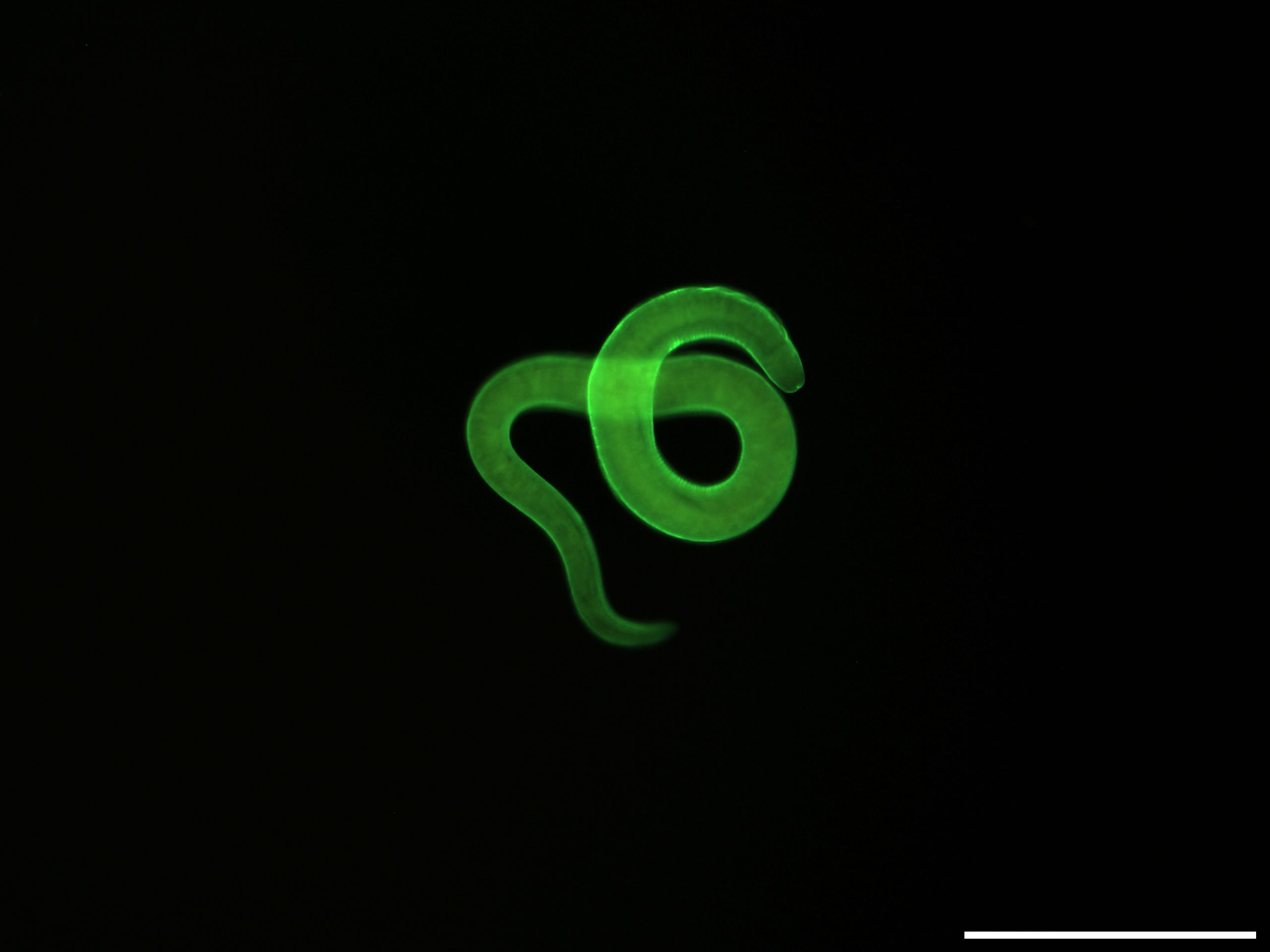

Supplement: Supplementary file 1 [file Data_Sheet_1.ZIP › 729402-supplementary material-original figures and dates-jpg-2021-7-2/729402 Fig5/Anti-rTsGS serum/Fig 5-31h+Anti-rTsGS serum.jpg]

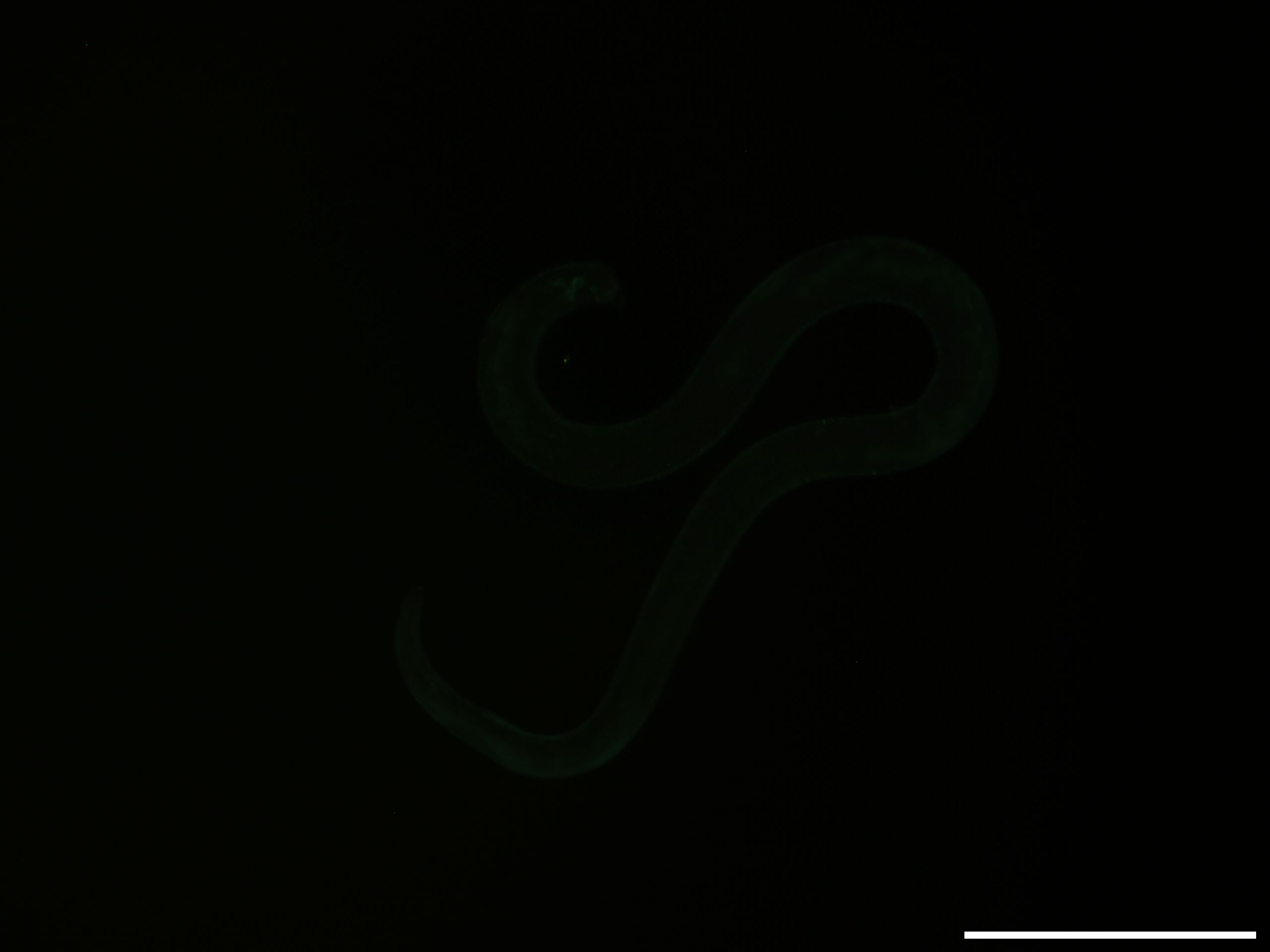

Supplement: Supplementary file 1 [file Data_Sheet_1.ZIP › 729402-supplementary material-original figures and dates-jpg-2021-7-2/729402 Fig5/Anti-rTsGS serum/Fig 5-3dAW+Anti-rTsGS serum.jpg]

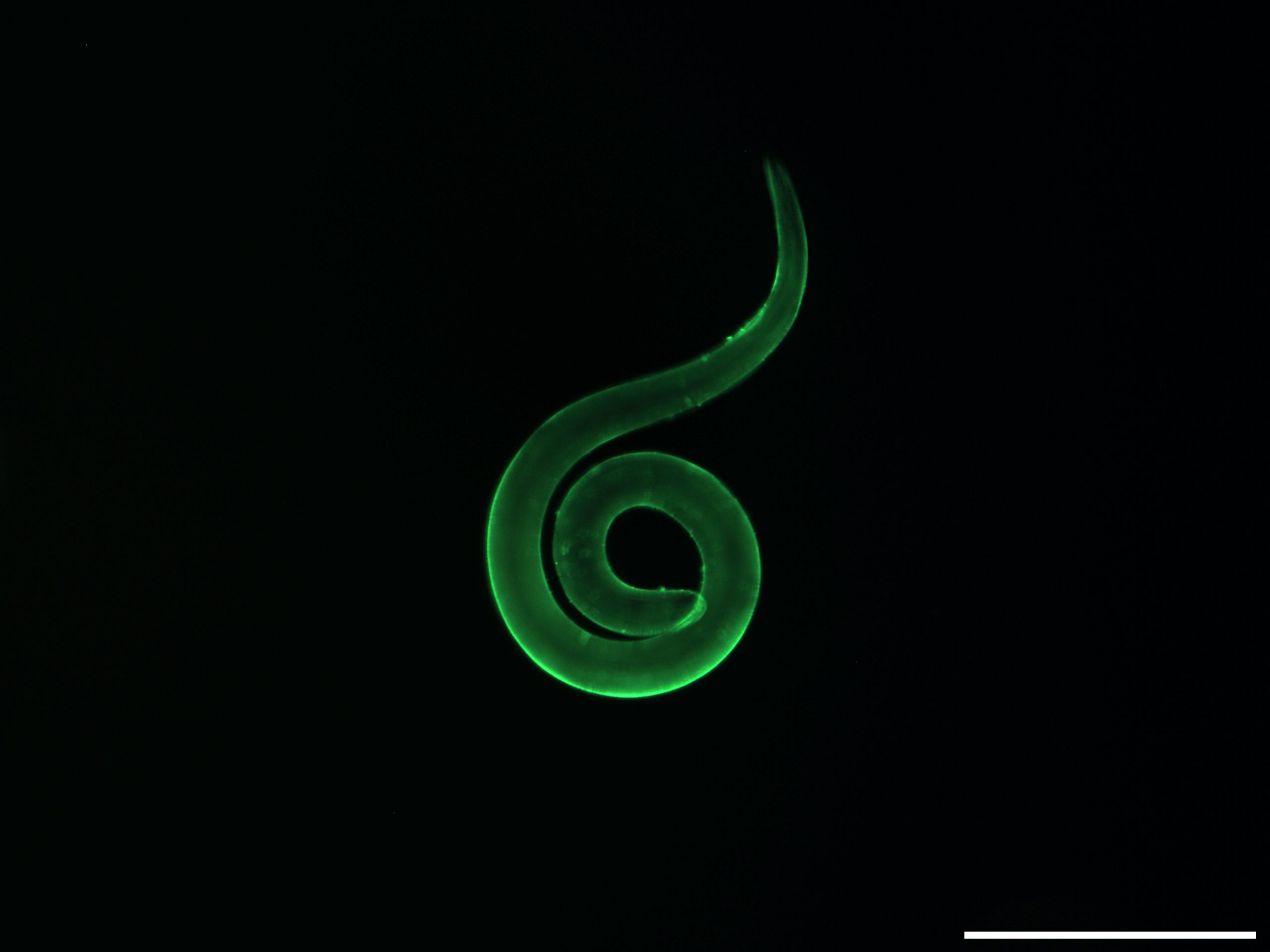

Supplement: Supplementary file 1 [file Data_Sheet_1.ZIP › 729402-supplementary material-original figures and dates-jpg-2021-7-2/729402 Fig5/Anti-rTsGS serum/Fig 5-6h+Anti-rTsGS serum.jpg]

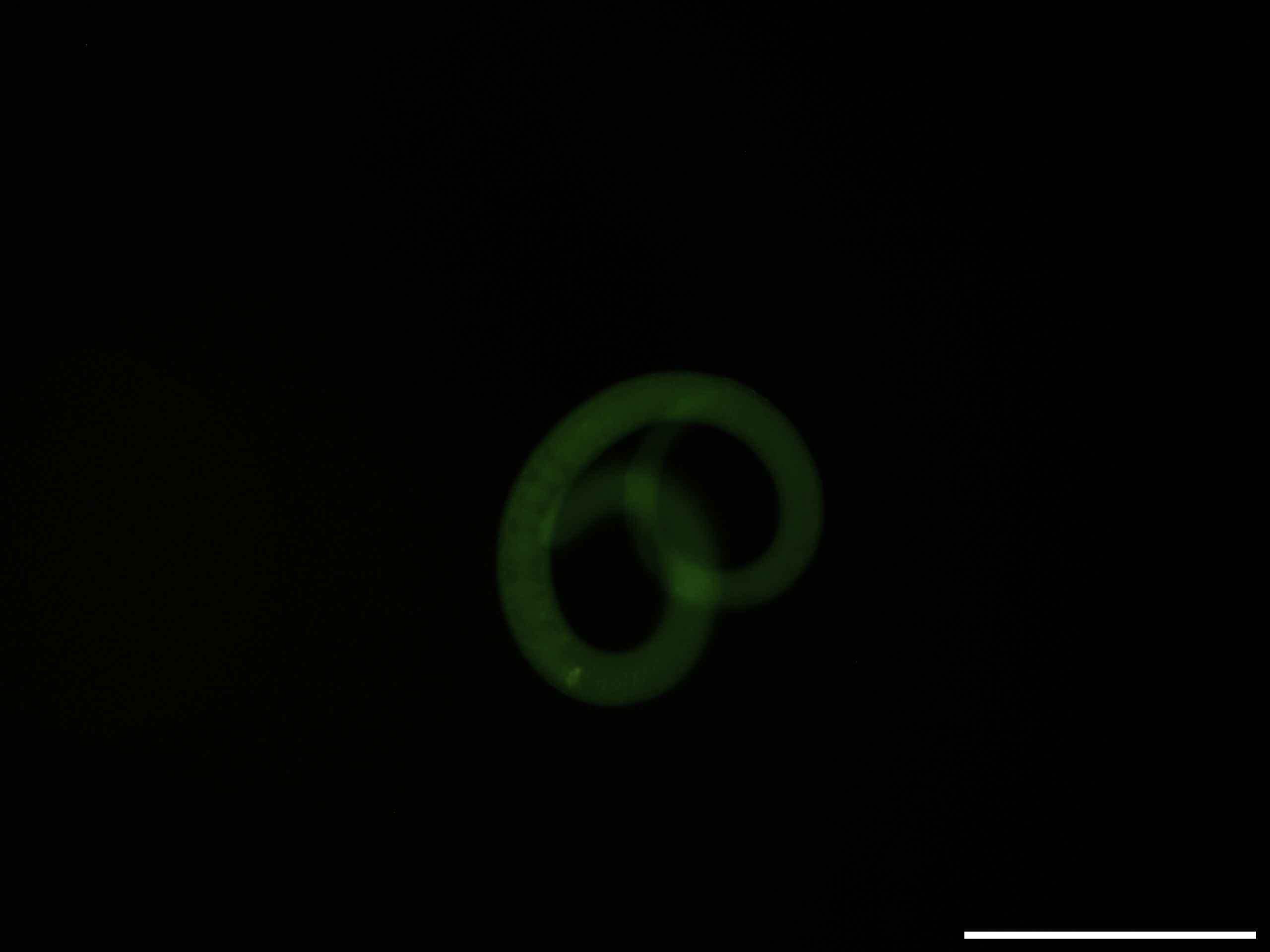

Supplement: Supplementary file 1 [file Data_Sheet_1.ZIP › 729402-supplementary material-original figures and dates-jpg-2021-7-2/729402 Fig5/Anti-rTsGS serum/Fig 5-ML+Anti-rTsGS serum.jpg]

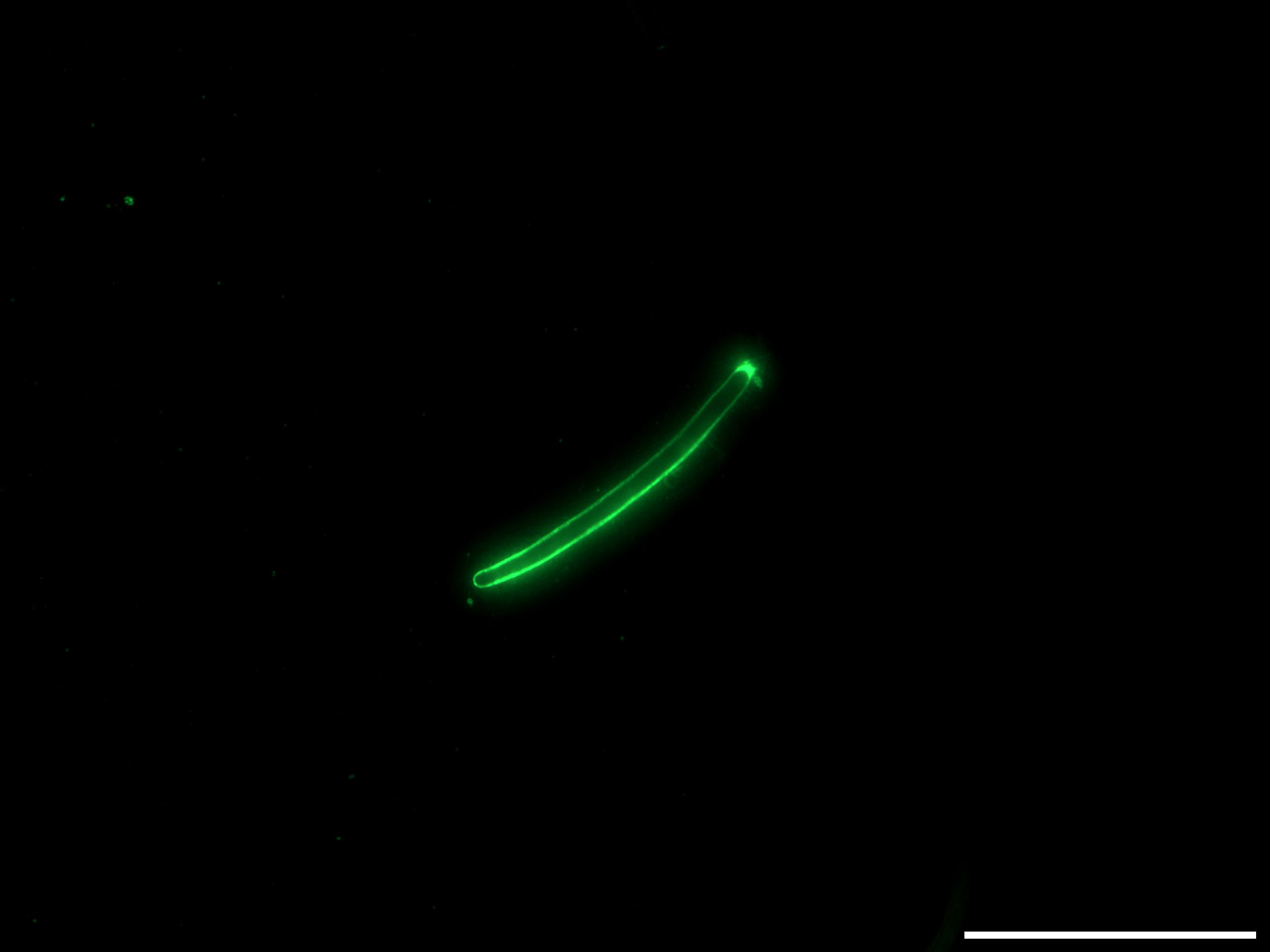

Supplement: Supplementary file 1 [file Data_Sheet_1.ZIP › 729402-supplementary material-original figures and dates-jpg-2021-7-2/729402 Fig5/Anti-rTsGS serum/Fig 5-NBL+Anti-rTsGS serum.jpg]

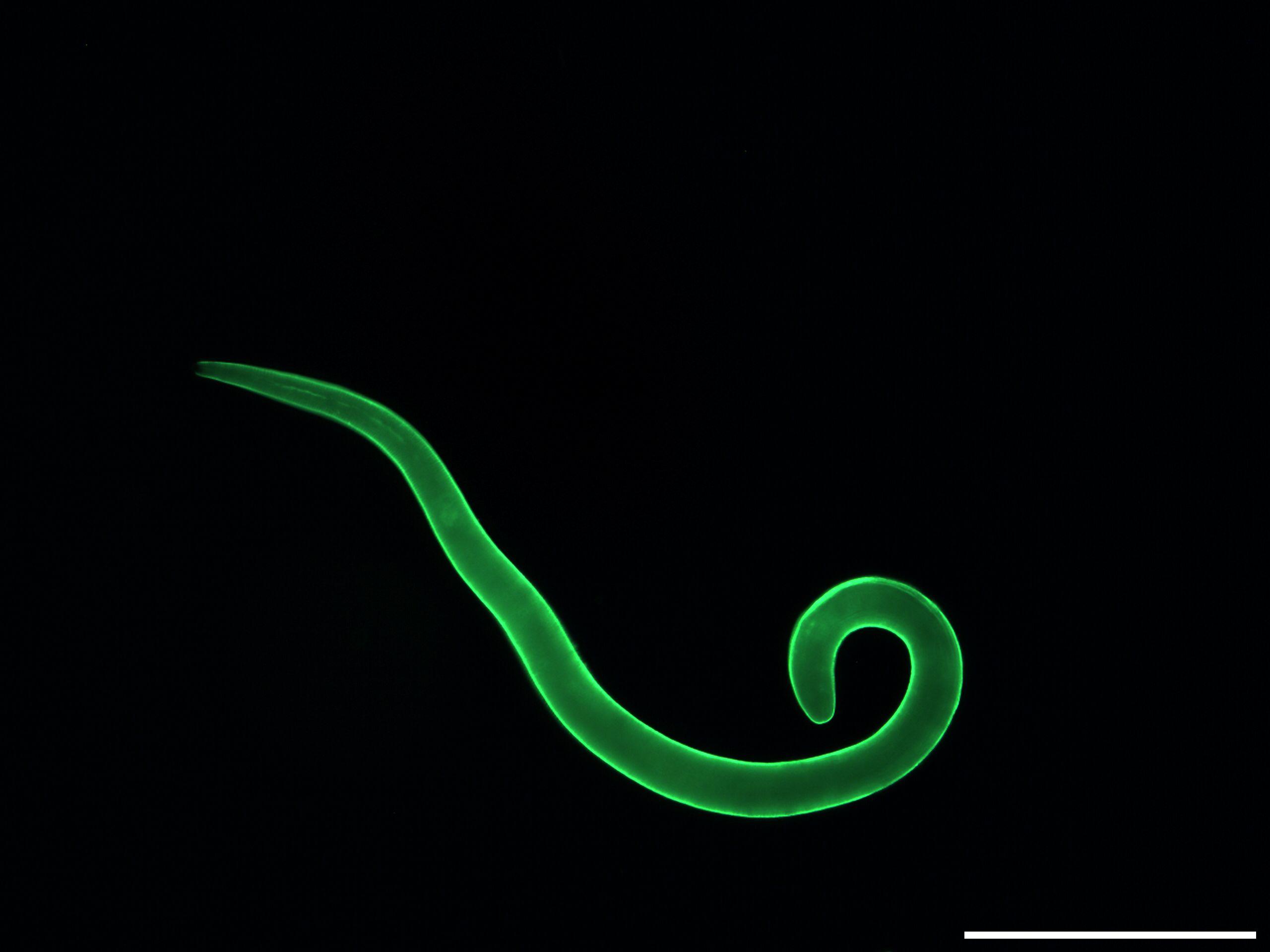

Supplement: Supplementary file 1 [file Data_Sheet_1.ZIP › 729402-supplementary material-original figures and dates-jpg-2021-7-2/729402 Fig5/Infection serum/Fig 5-10h+Infection serum.jpg]

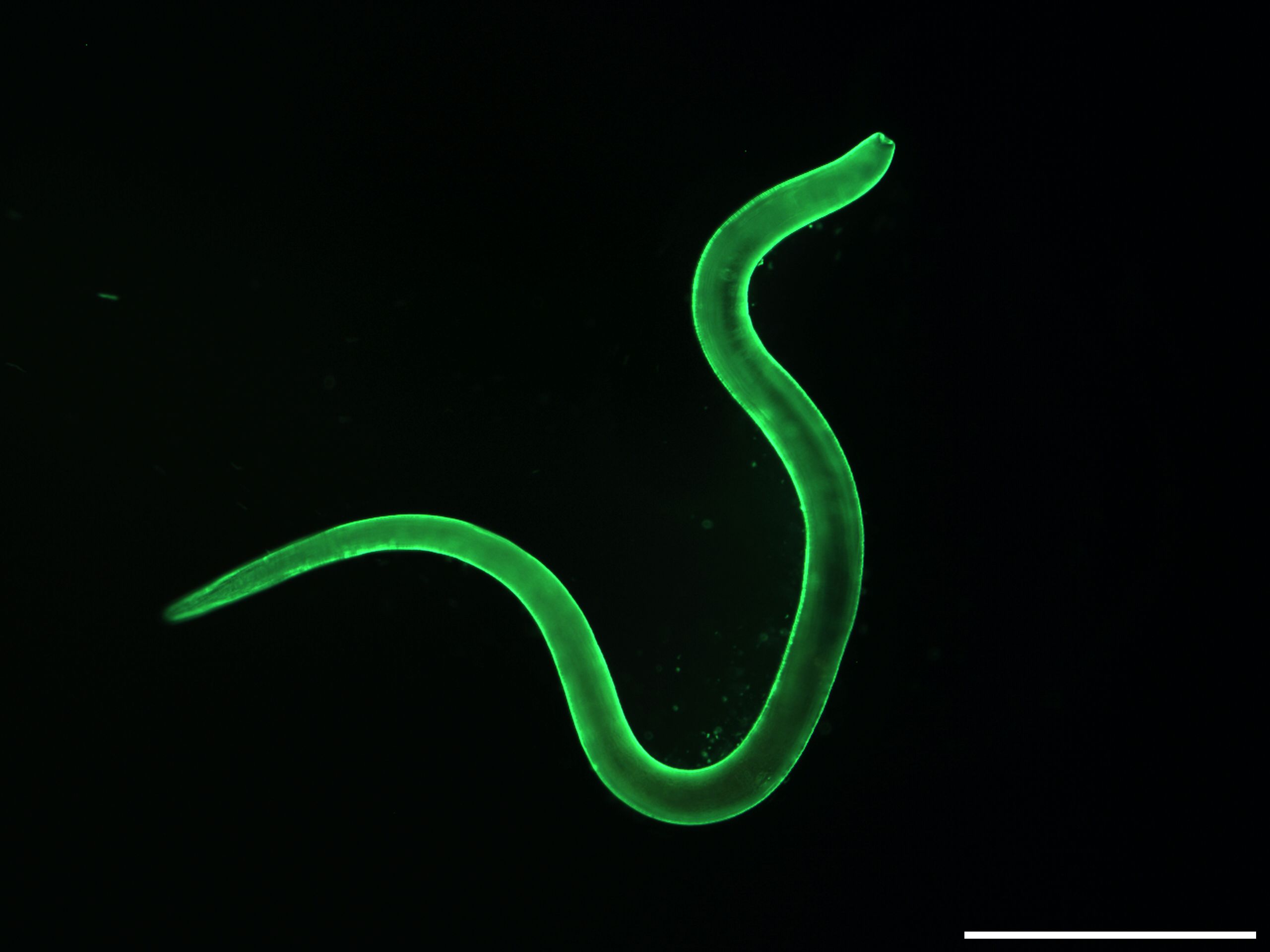

Supplement: Supplementary file 1 [file Data_Sheet_1.ZIP › 729402-supplementary material-original figures and dates-jpg-2021-7-2/729402 Fig5/Infection serum/Fig 5-12h+Infection serum.jpg]

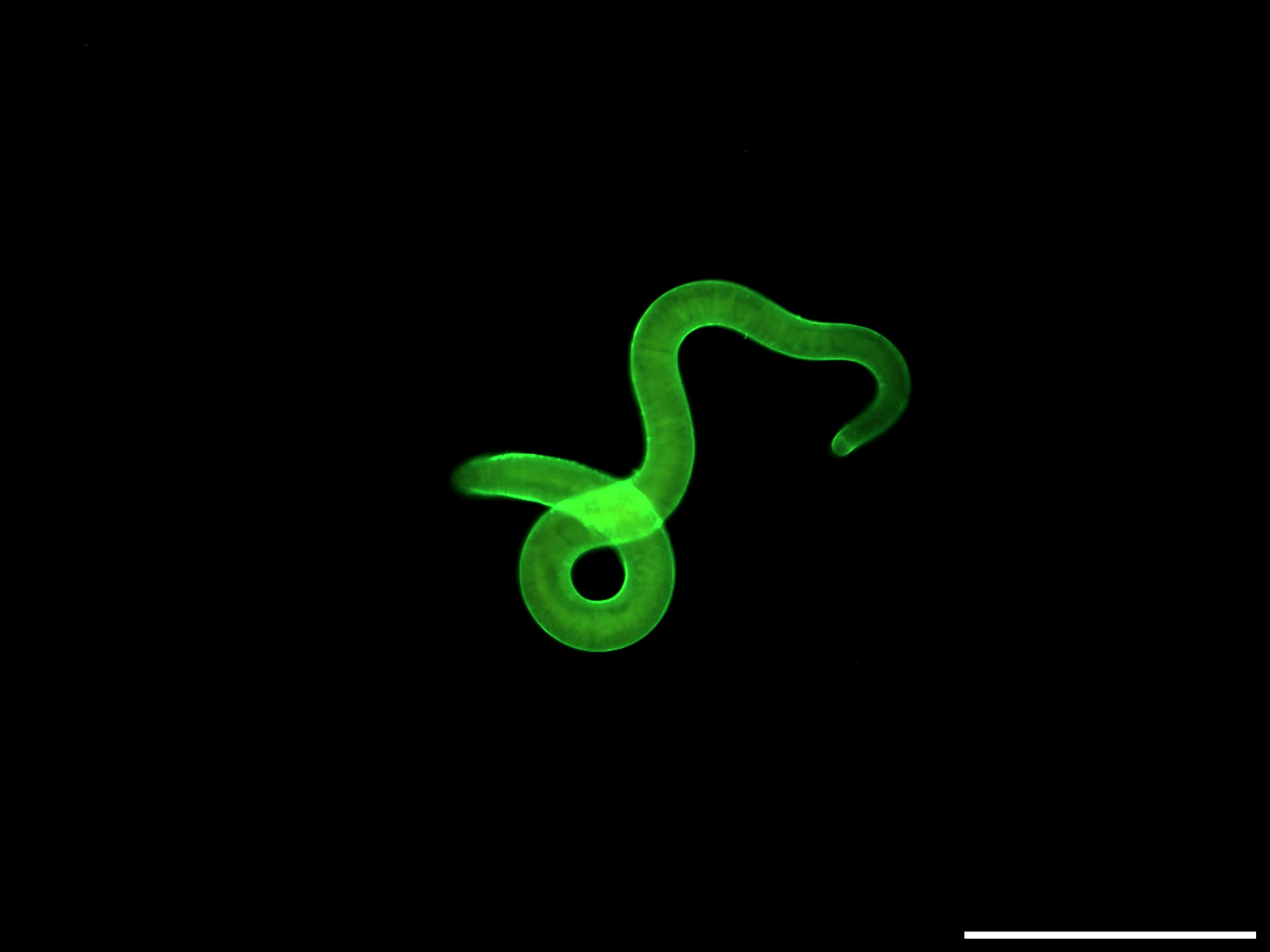

Supplement: Supplementary file 1 [file Data_Sheet_1.ZIP › 729402-supplementary material-original figures and dates-jpg-2021-7-2/729402 Fig5/Infection serum/Fig 5-15h+Infection serum.jpg]

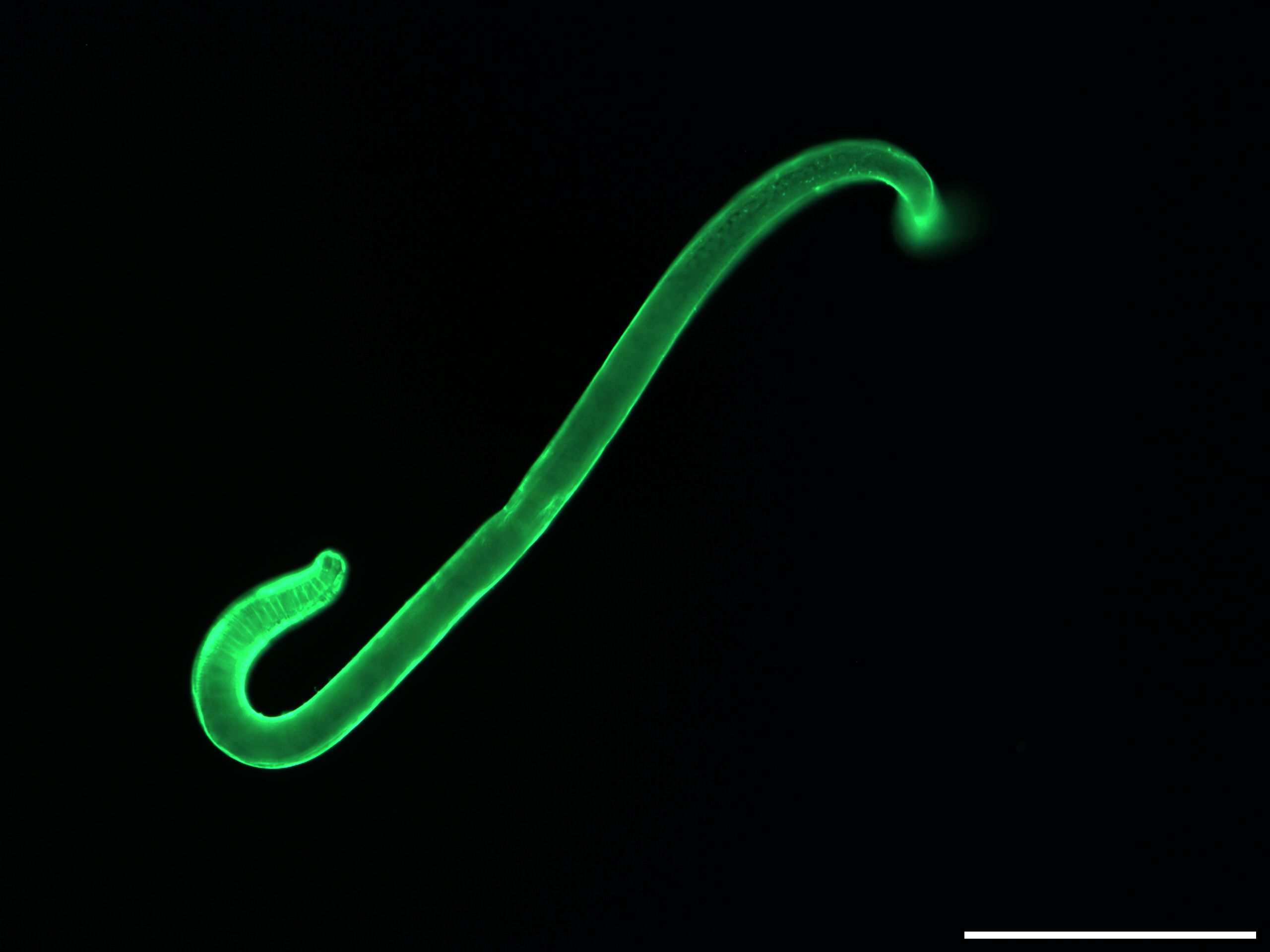

Supplement: Supplementary file 1 [file Data_Sheet_1.ZIP › 729402-supplementary material-original figures and dates-jpg-2021-7-2/729402 Fig5/Infection serum/Fig 5-18h+Infection serum.jpg]

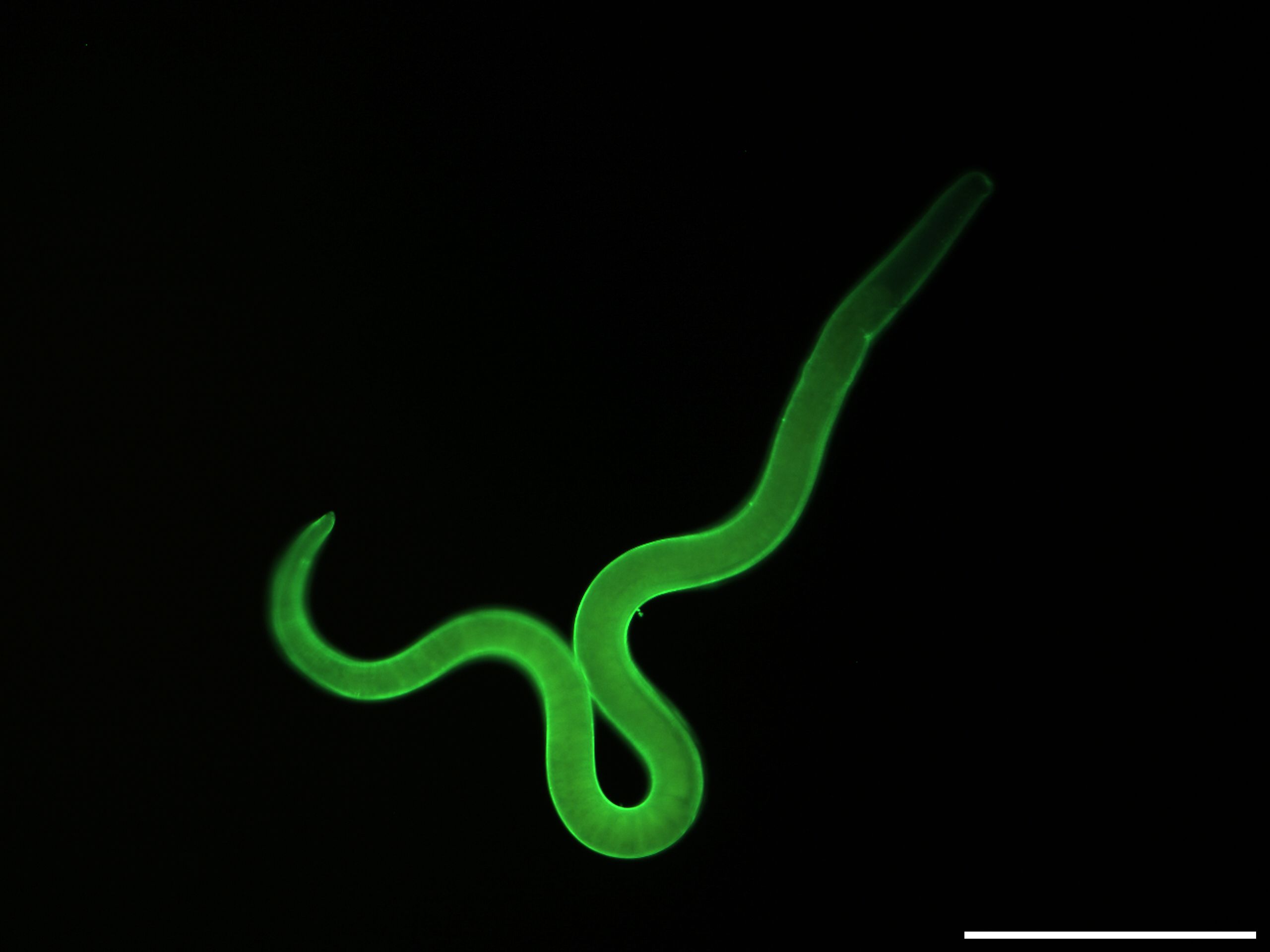

Supplement: Supplementary file 1 [file Data_Sheet_1.ZIP › 729402-supplementary material-original figures and dates-jpg-2021-7-2/729402 Fig5/Infection serum/Fig 5-27h+Infection serum.jpg]

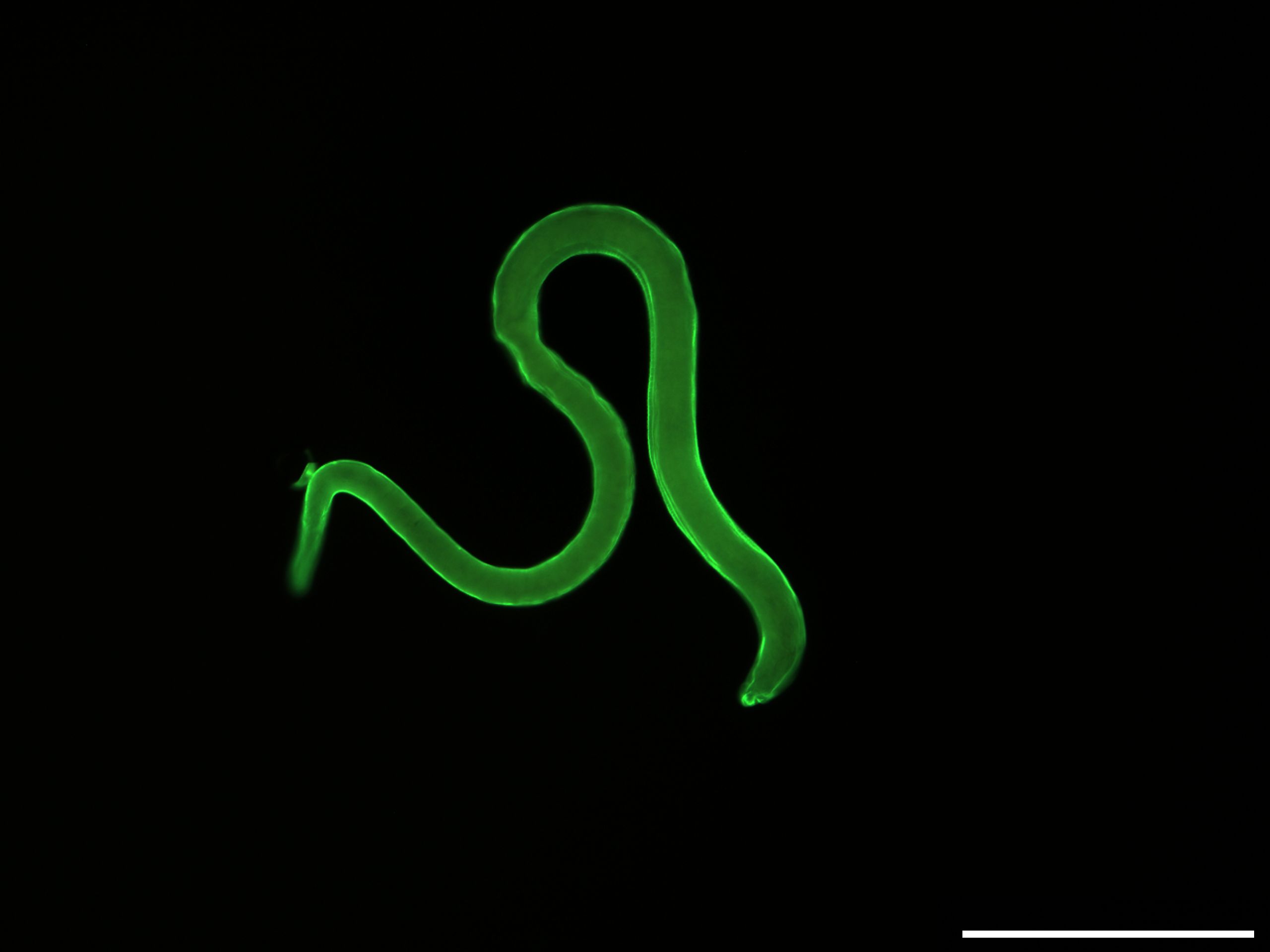

Supplement: Supplementary file 1 [file Data_Sheet_1.ZIP › 729402-supplementary material-original figures and dates-jpg-2021-7-2/729402 Fig5/Infection serum/Fig 5-31h+Infection serum.jpg]

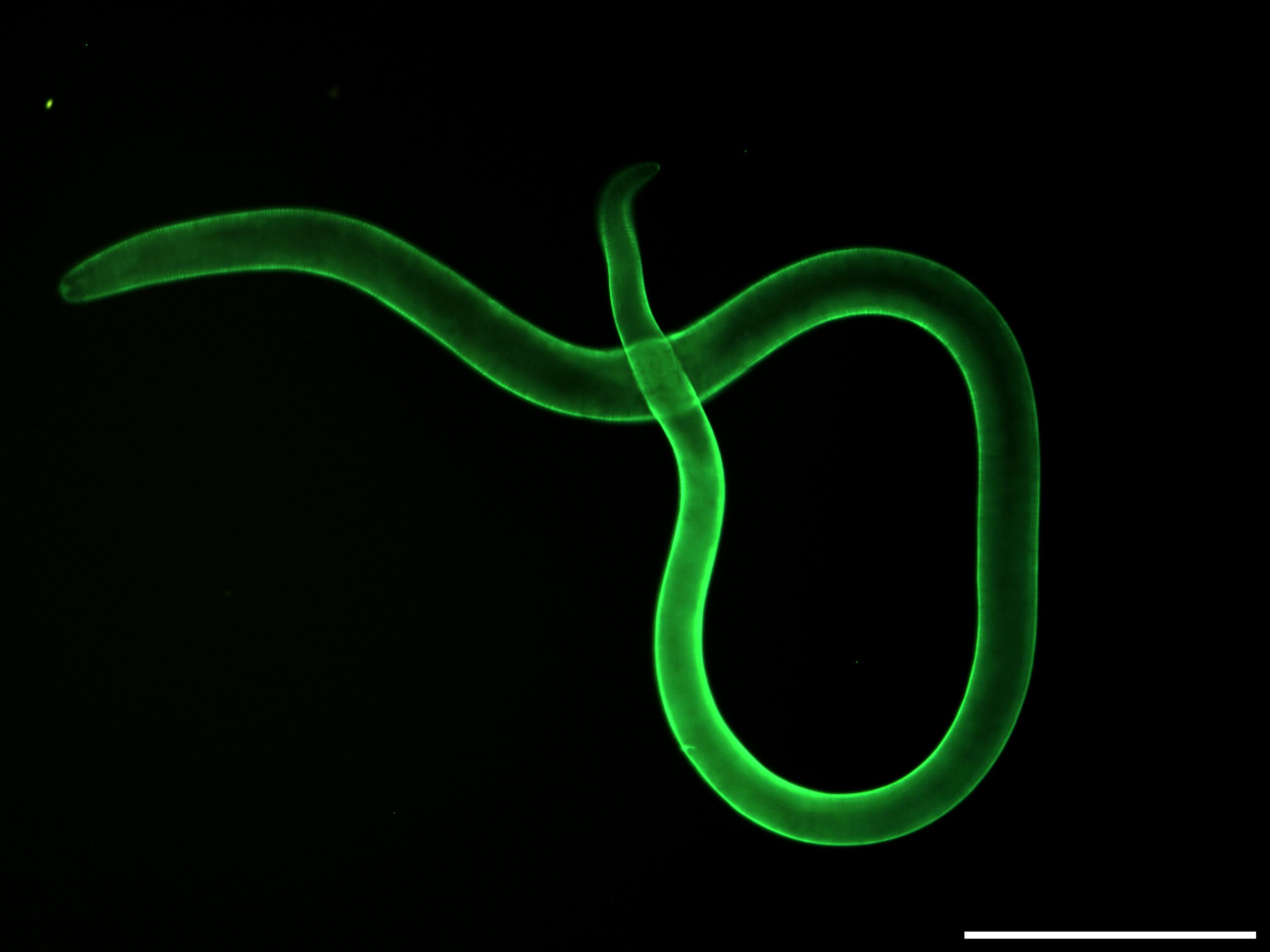

Supplement: Supplementary file 1 [file Data_Sheet_1.ZIP › 729402-supplementary material-original figures and dates-jpg-2021-7-2/729402 Fig5/Infection serum/Fig 5-3d+Infection serum.jpg]

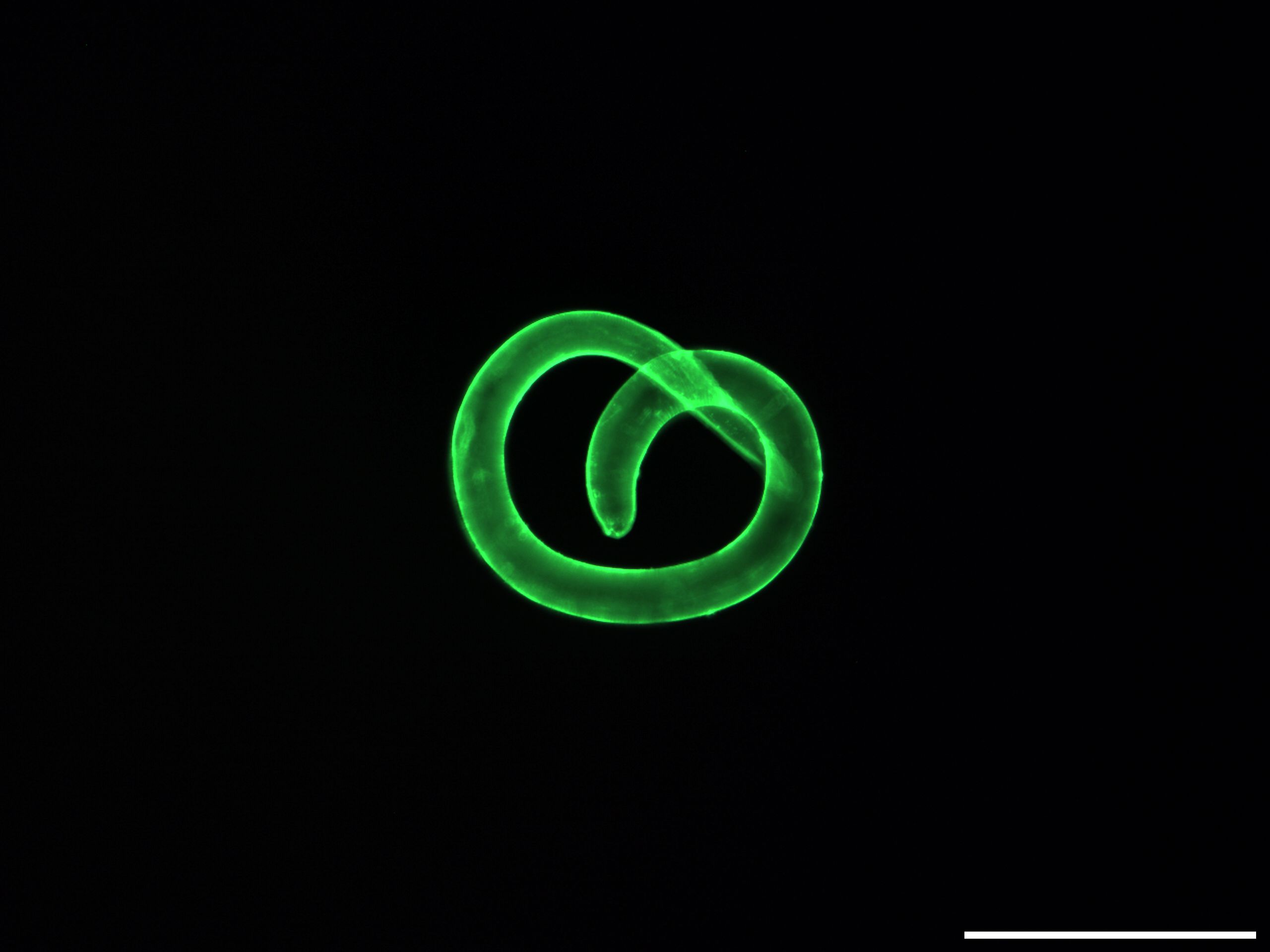

Supplement: Supplementary file 1 [file Data_Sheet_1.ZIP › 729402-supplementary material-original figures and dates-jpg-2021-7-2/729402 Fig5/Infection serum/Fig 5-6h+Infection serum.jpg]

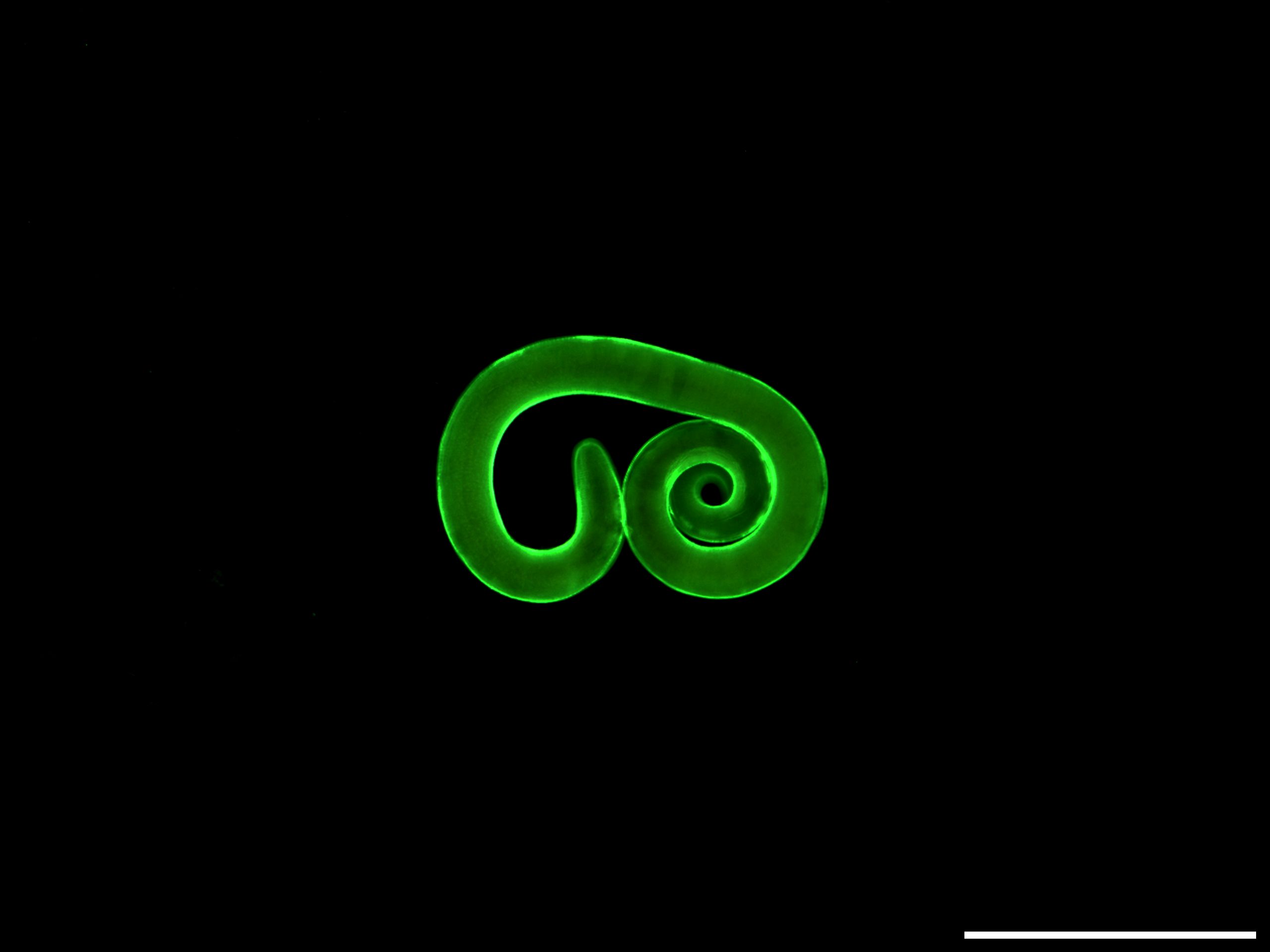

Supplement: Supplementary file 1 [file Data_Sheet_1.ZIP › 729402-supplementary material-original figures and dates-jpg-2021-7-2/729402 Fig5/Infection serum/Fig 5-ML+Infection serum.jpg]

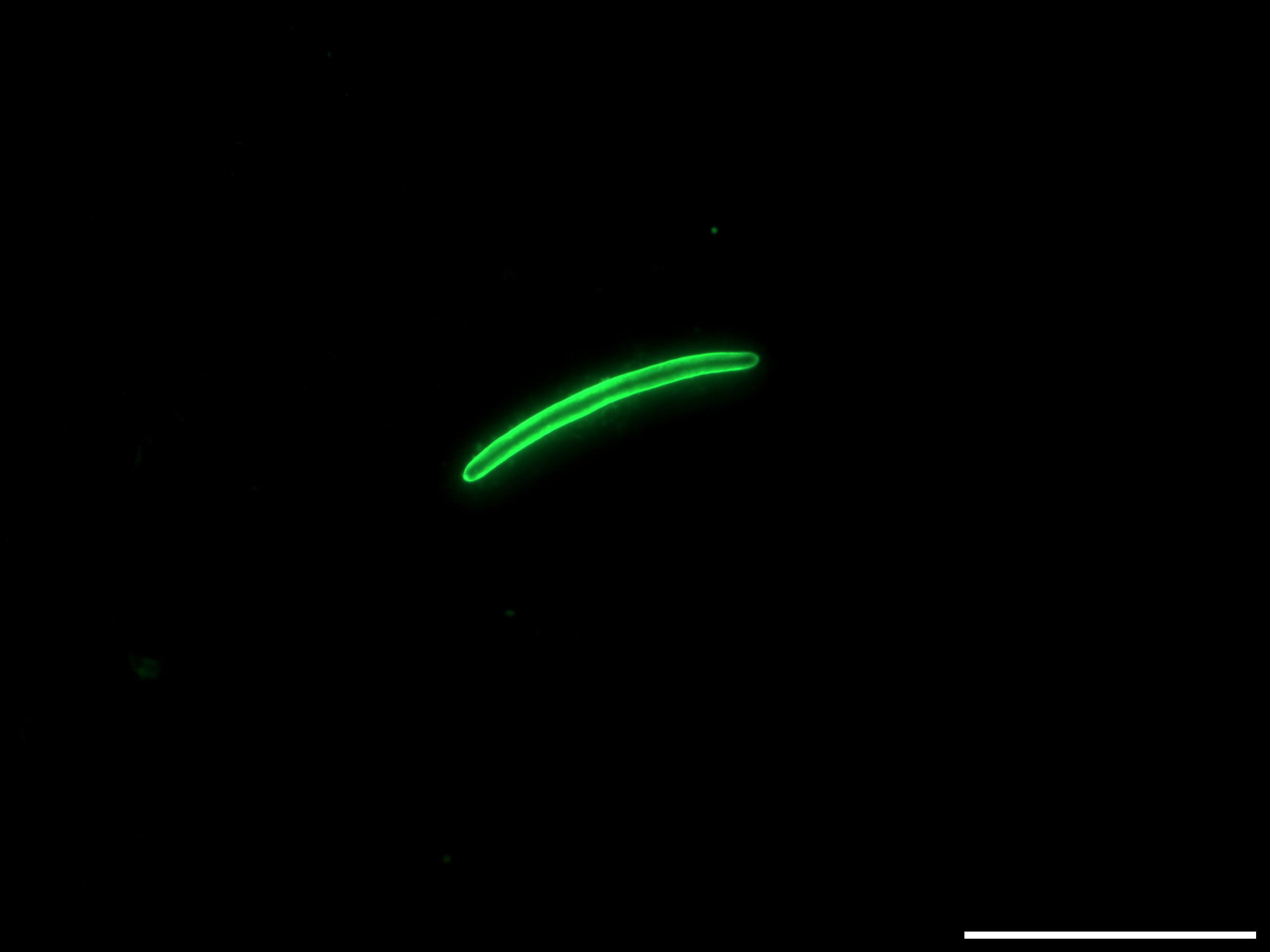

Supplement: Supplementary file 1 [file Data_Sheet_1.ZIP › 729402-supplementary material-original figures and dates-jpg-2021-7-2/729402 Fig5/Infection serum/Fig 5-NBL+Infection serum.jpg]

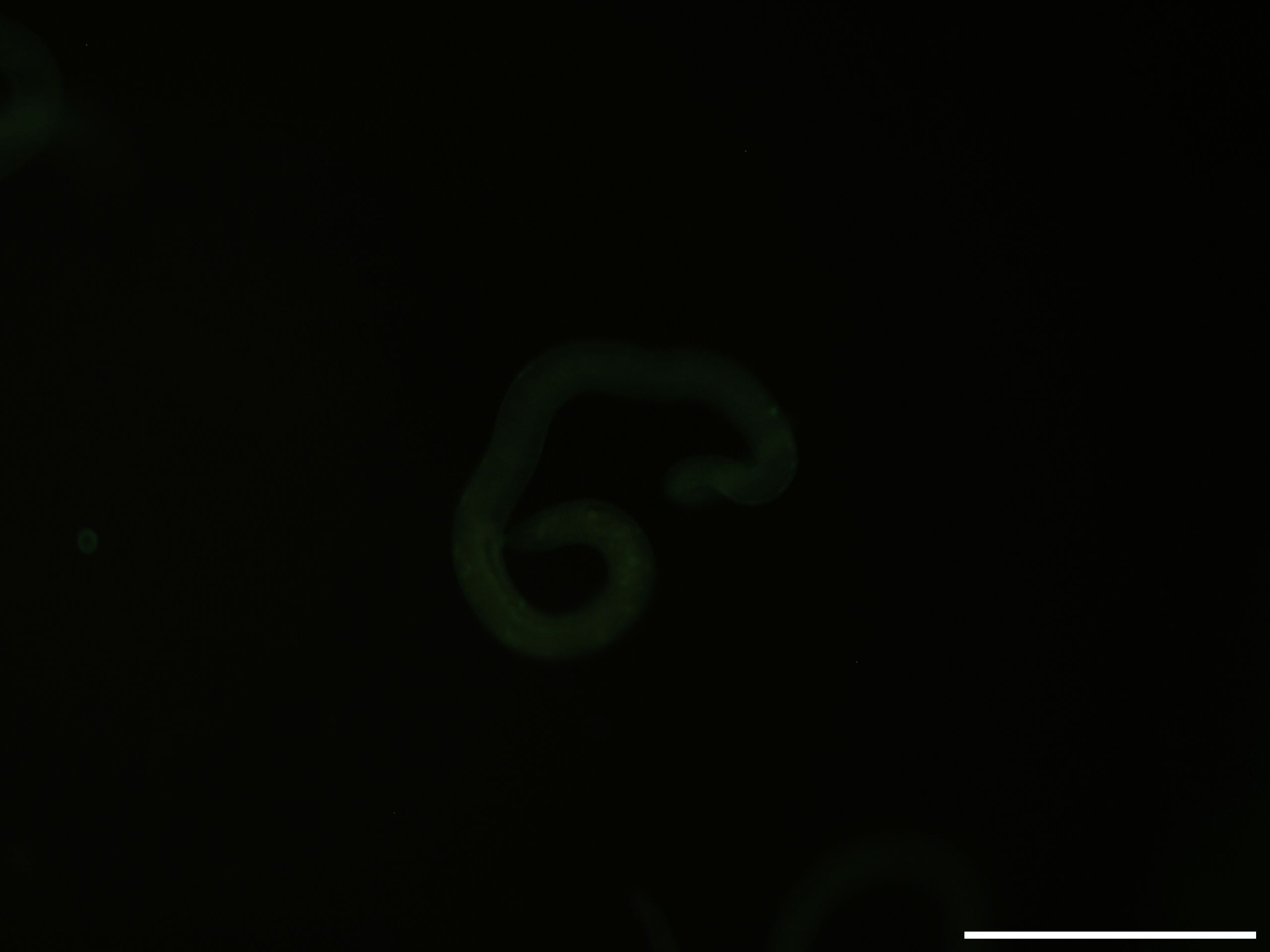

Supplement: Supplementary file 1 [file Data_Sheet_1.ZIP › 729402-supplementary material-original figures and dates-jpg-2021-7-2/729402 Fig5/Normal serum/Fig 5-10h+Normal serum.jpg]

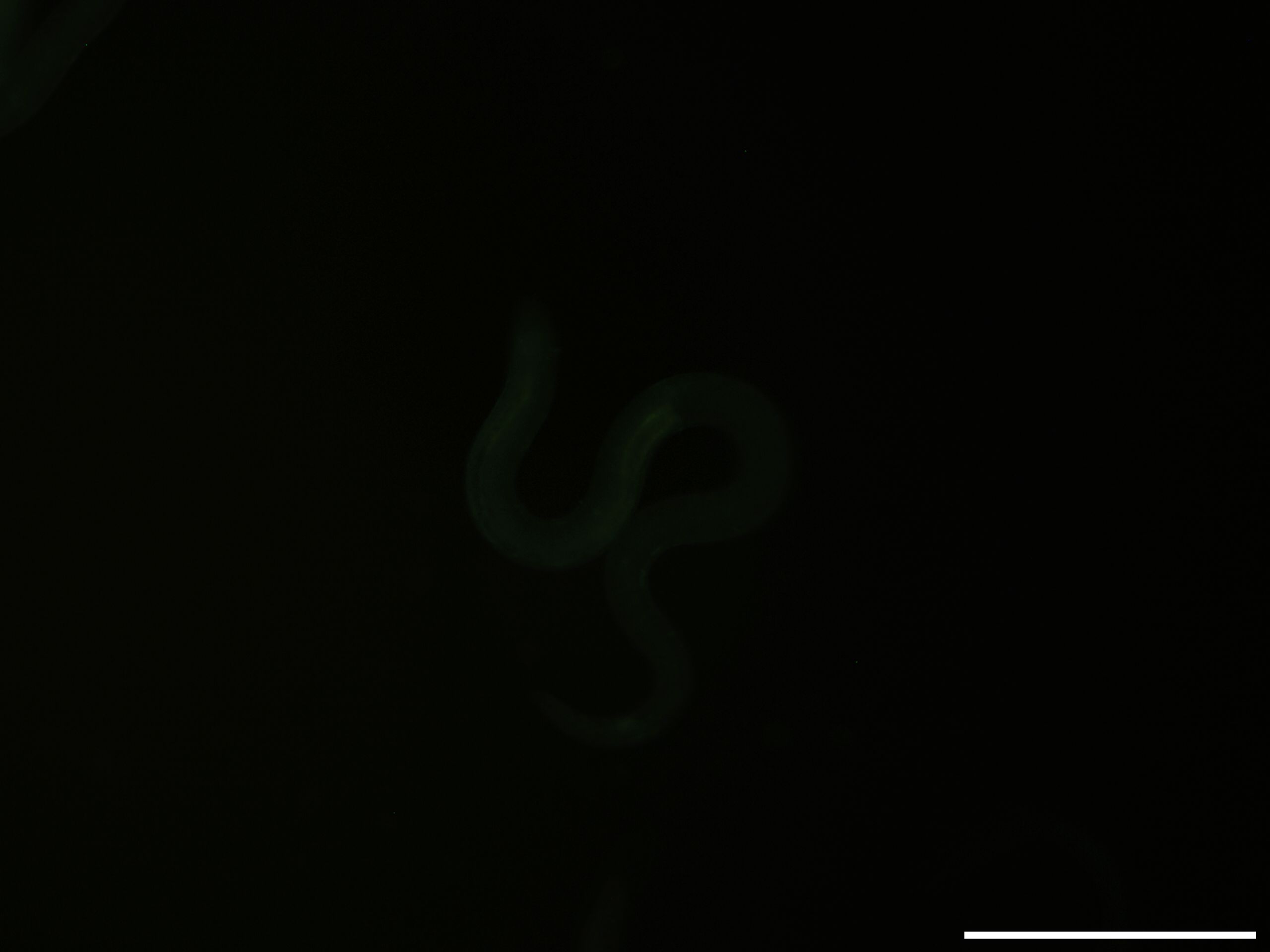

Supplement: Supplementary file 1 [file Data_Sheet_1.ZIP › 729402-supplementary material-original figures and dates-jpg-2021-7-2/729402 Fig5/Normal serum/Fig 5-12h+Normal serum.jpg]

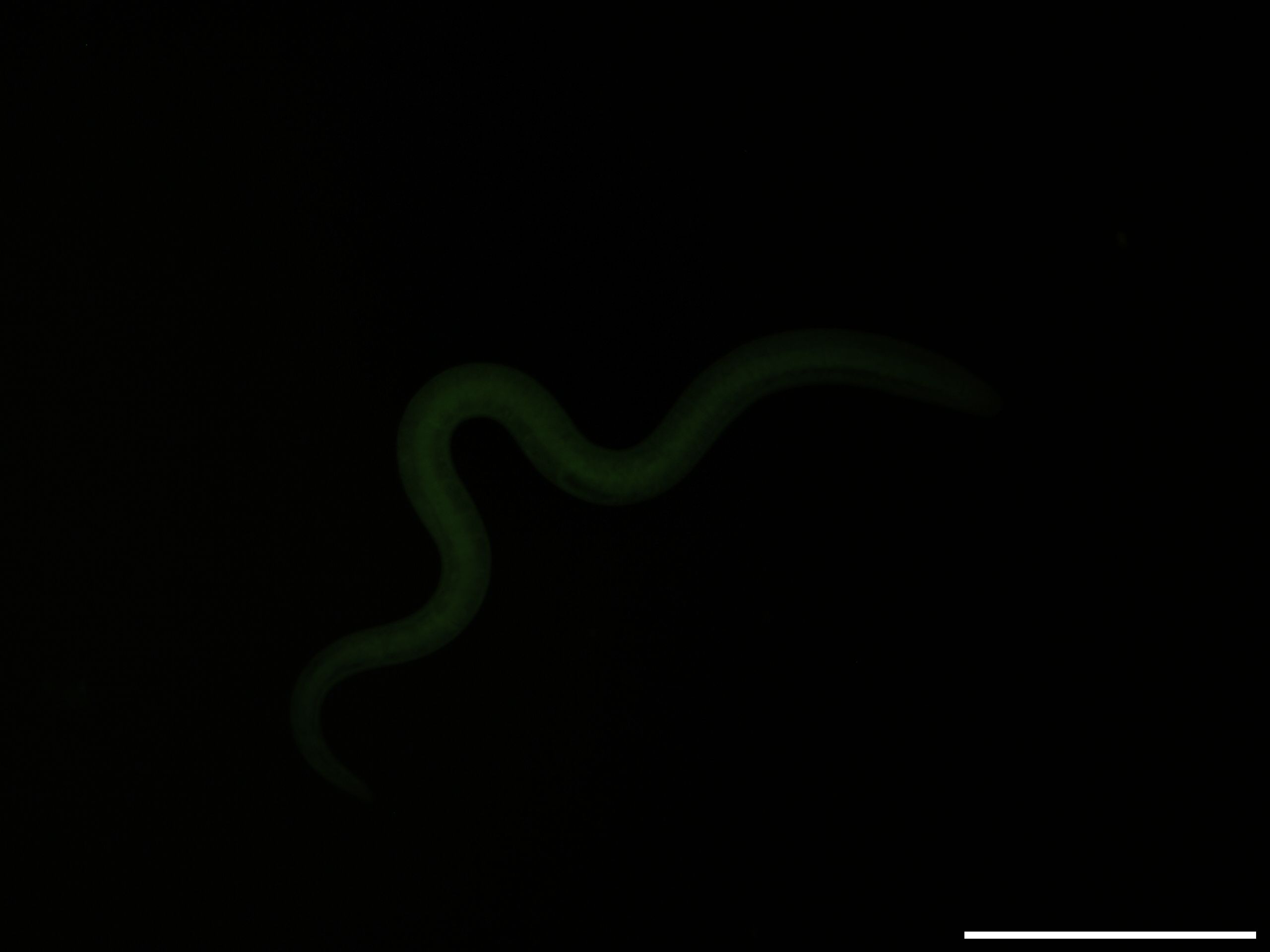

Supplement: Supplementary file 1 [file Data_Sheet_1.ZIP › 729402-supplementary material-original figures and dates-jpg-2021-7-2/729402 Fig5/Normal serum/Fig 5-15h+Normal serum.jpg]

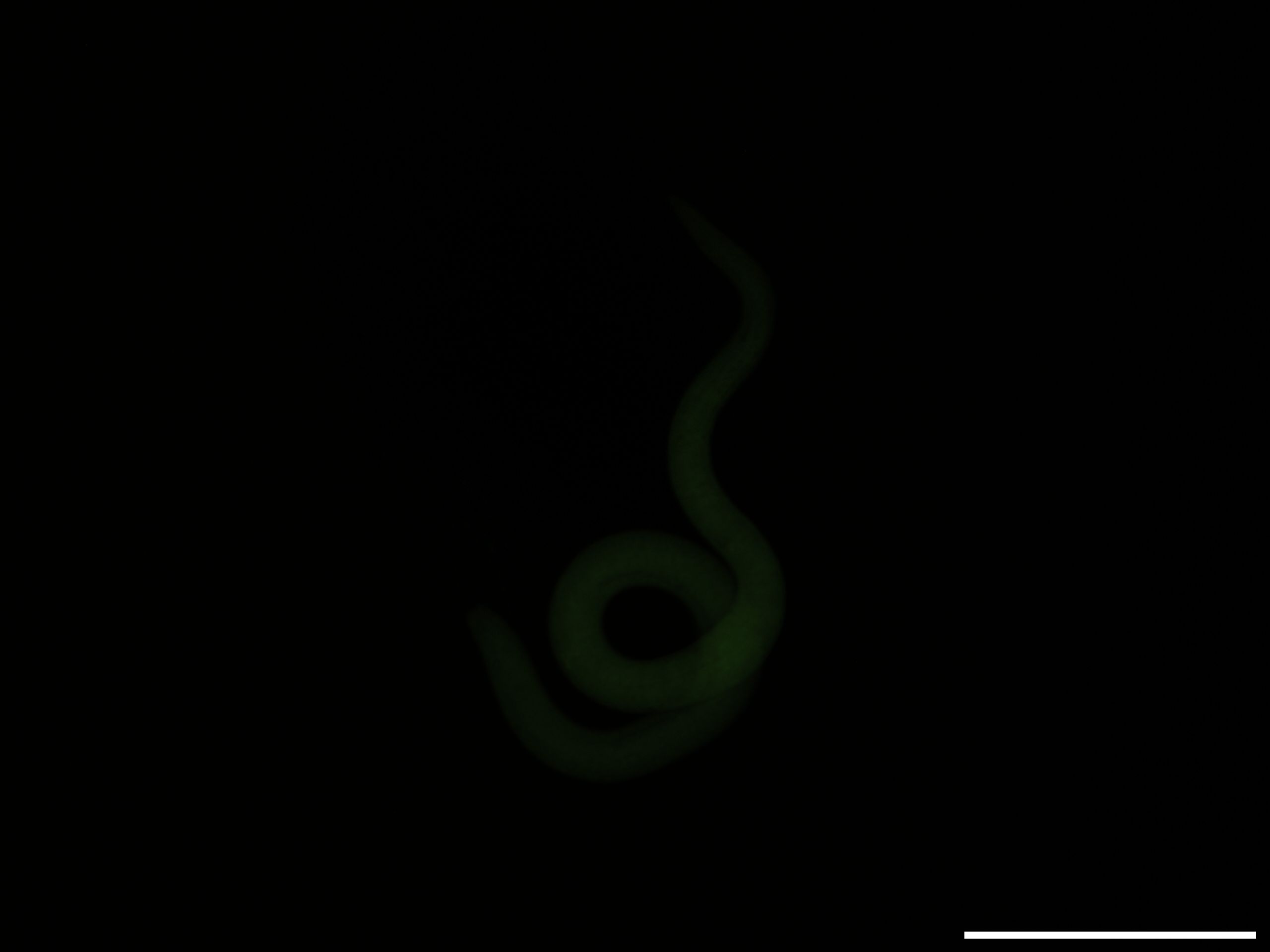

Supplement: Supplementary file 1 [file Data_Sheet_1.ZIP › 729402-supplementary material-original figures and dates-jpg-2021-7-2/729402 Fig5/Normal serum/Fig 5-18h+Normal serum.jpg]

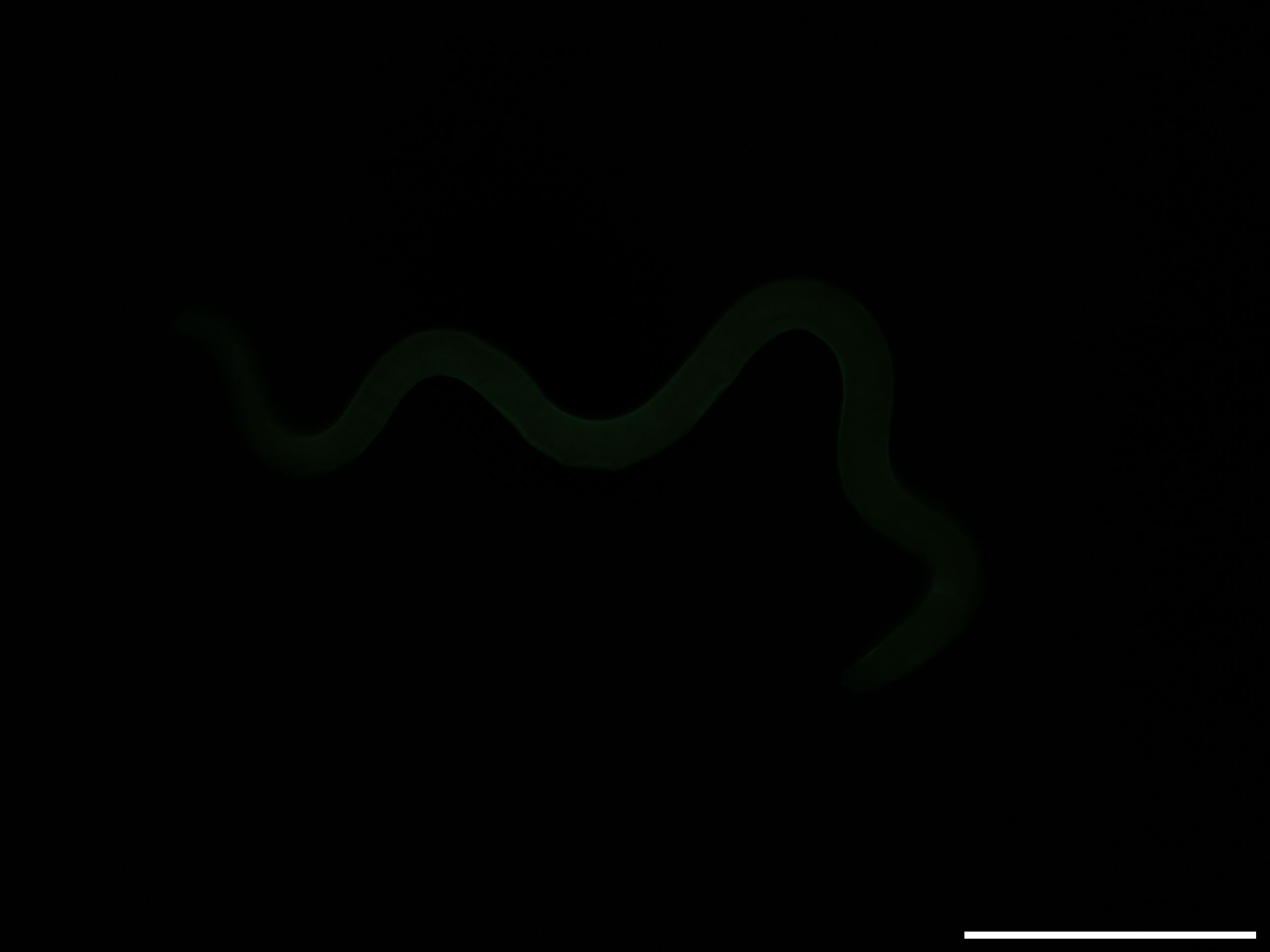

Supplement: Supplementary file 1 [file Data_Sheet_1.ZIP › 729402-supplementary material-original figures and dates-jpg-2021-7-2/729402 Fig5/Normal serum/Fig 5-27h+Normal serum.jpg]

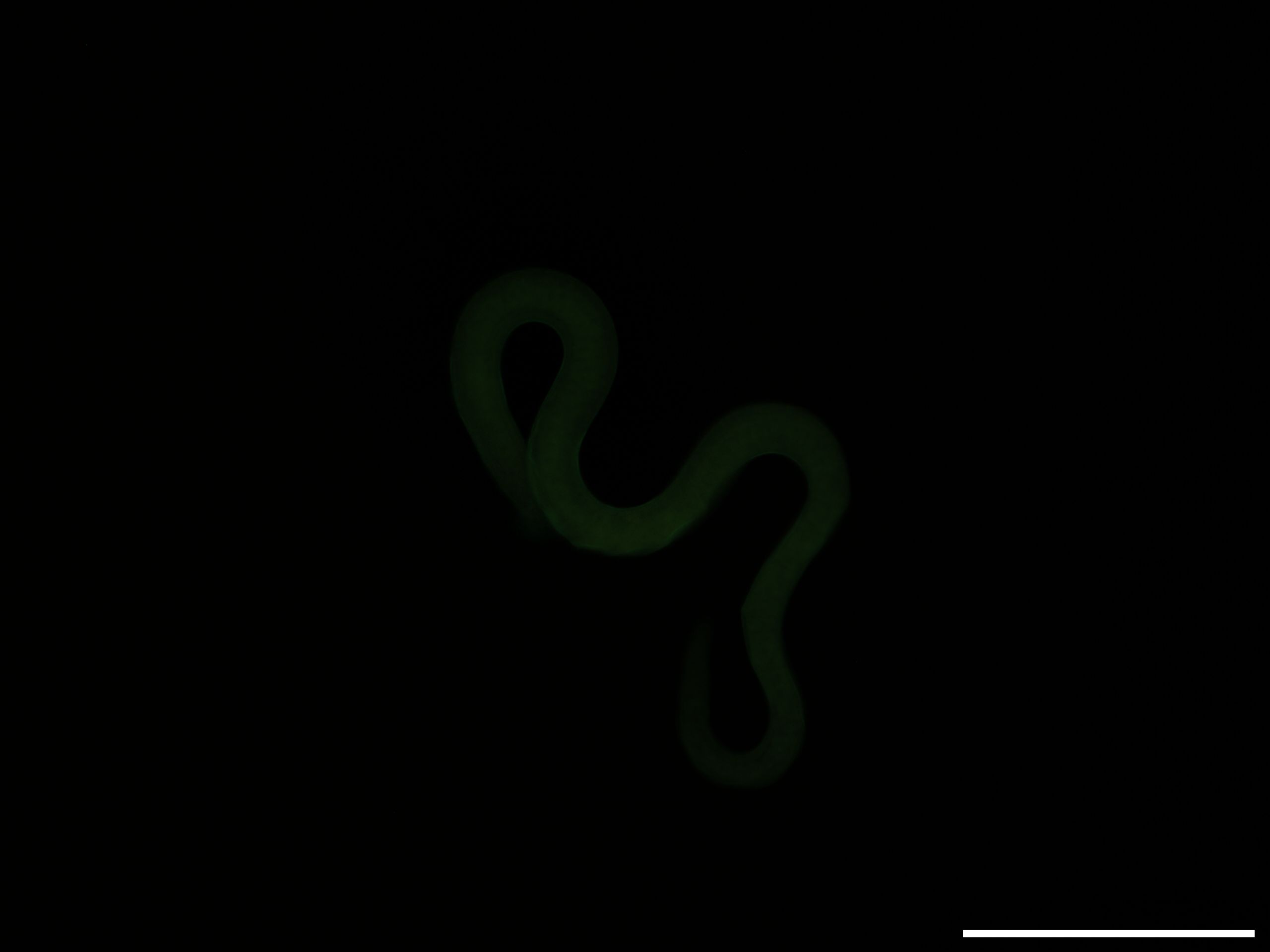

Supplement: Supplementary file 1 [file Data_Sheet_1.ZIP › 729402-supplementary material-original figures and dates-jpg-2021-7-2/729402 Fig5/Normal serum/Fig 5-31h +Normal serum.jpg]

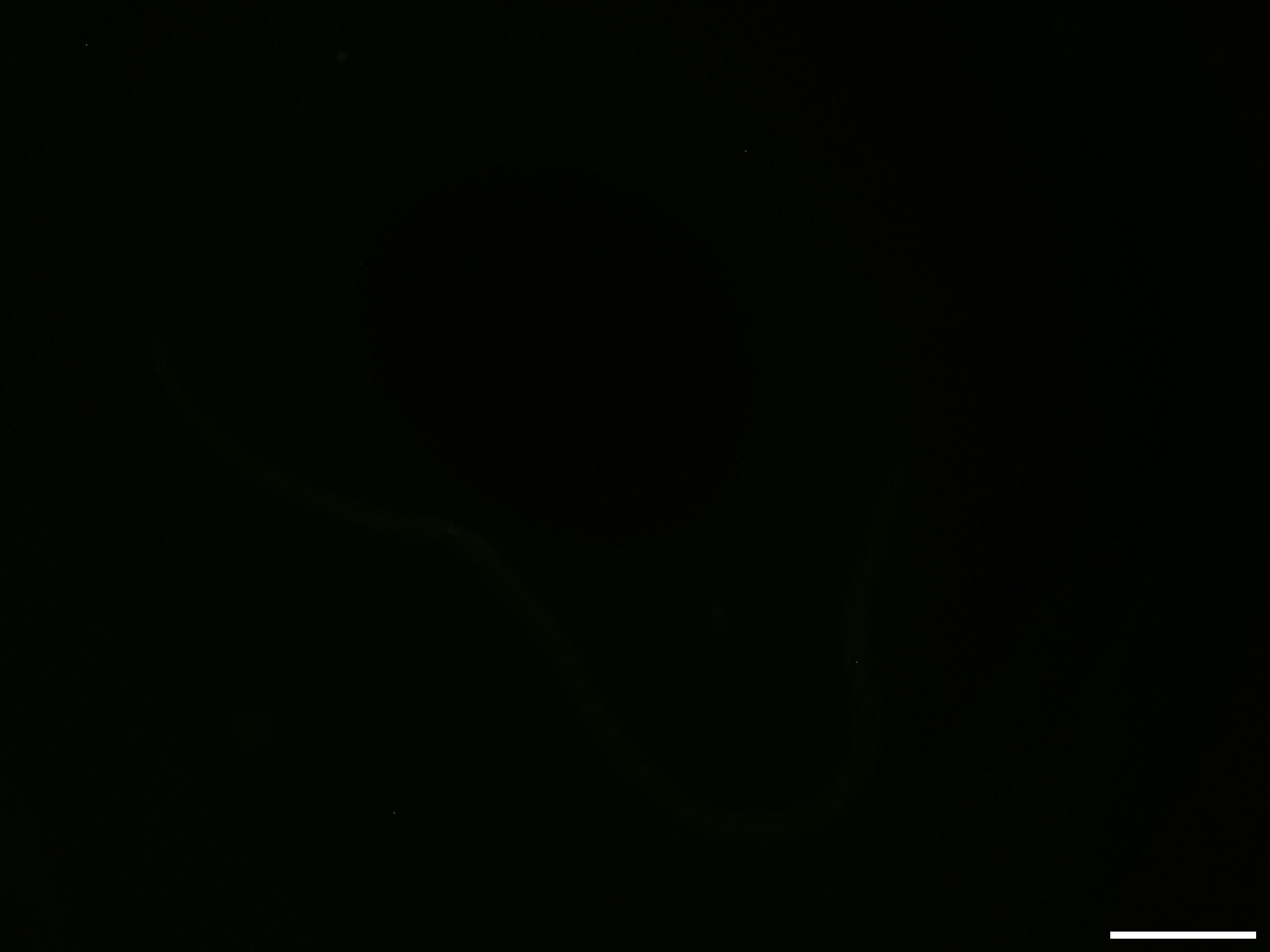

Supplement: Supplementary file 1 [file Data_Sheet_1.ZIP › 729402-supplementary material-original figures and dates-jpg-2021-7-2/729402 Fig5/Normal serum/Fig 5-3d+Normal serum.jpg]

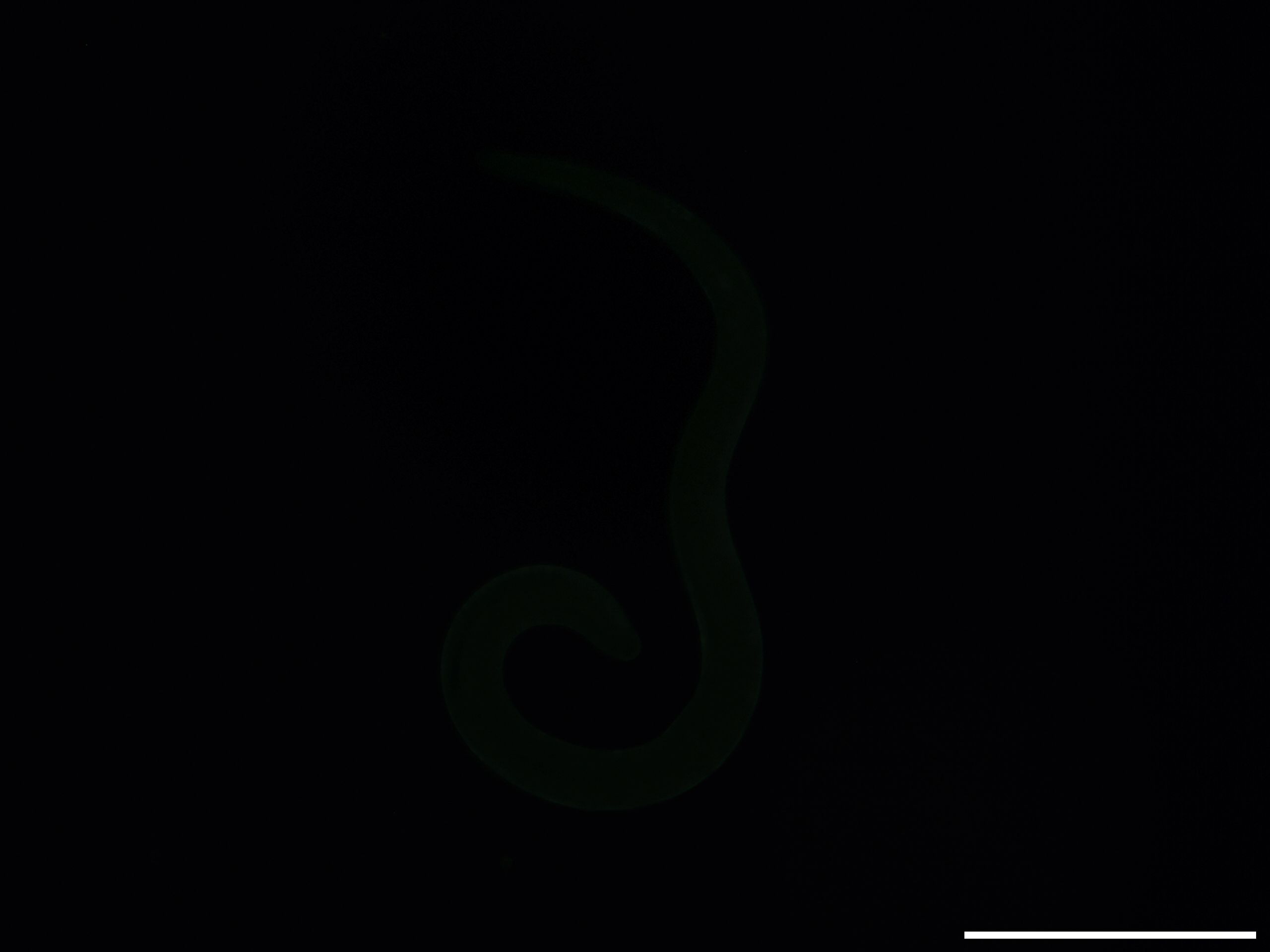

Supplement: Supplementary file 1 [file Data_Sheet_1.ZIP › 729402-supplementary material-original figures and dates-jpg-2021-7-2/729402 Fig5/Normal serum/Fig 5-6h+Normal serum.jpg]

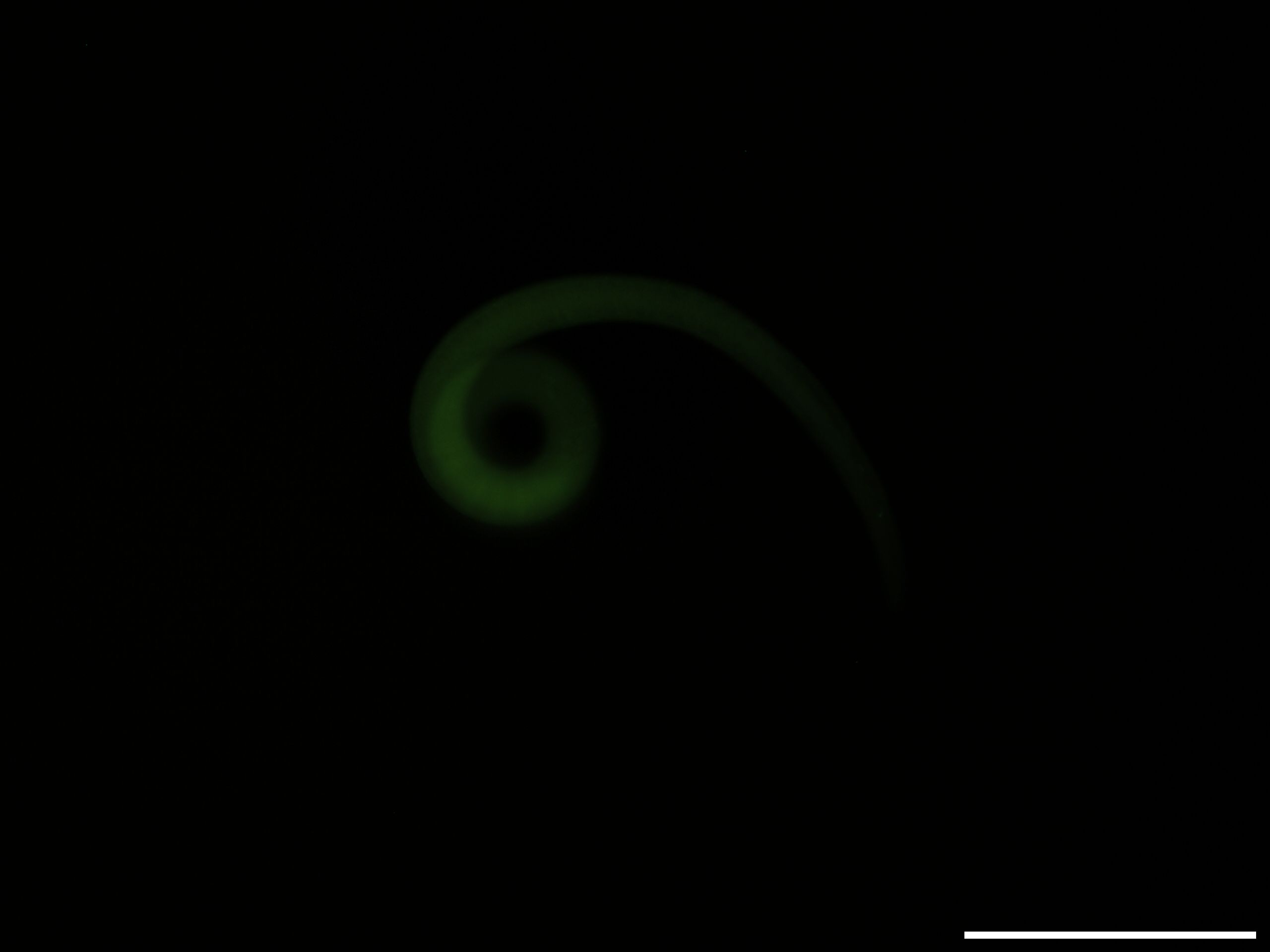

Supplement: Supplementary file 1 [file Data_Sheet_1.ZIP › 729402-supplementary material-original figures and dates-jpg-2021-7-2/729402 Fig5/Normal serum/Fig 5-ML+Normal serum.jpg]

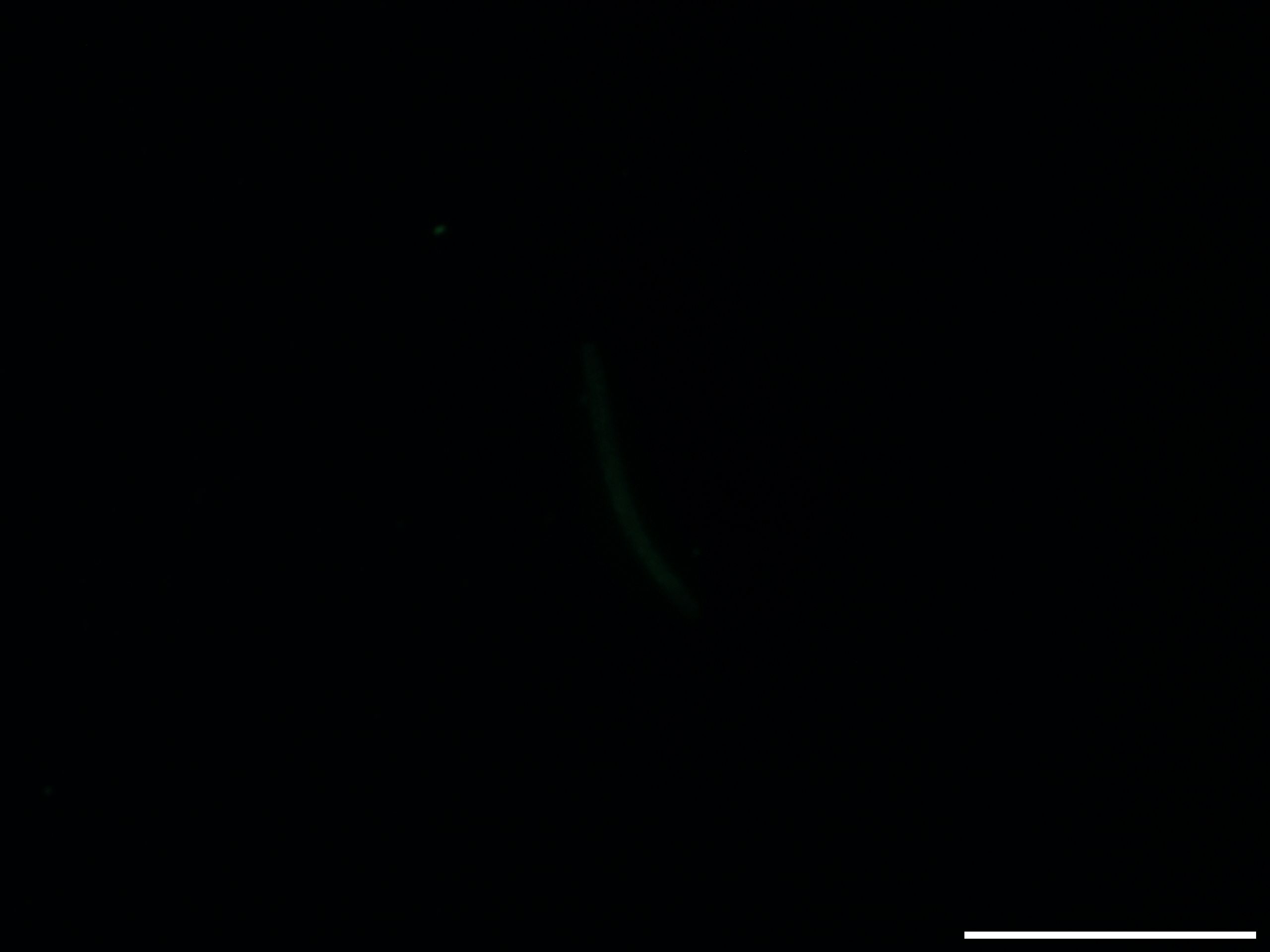

Supplement: Supplementary file 1 [file Data_Sheet_1.ZIP › 729402-supplementary material-original figures and dates-jpg-2021-7-2/729402 Fig5/Normal serum/Fig 5-NBL+Normal serum.jpg]

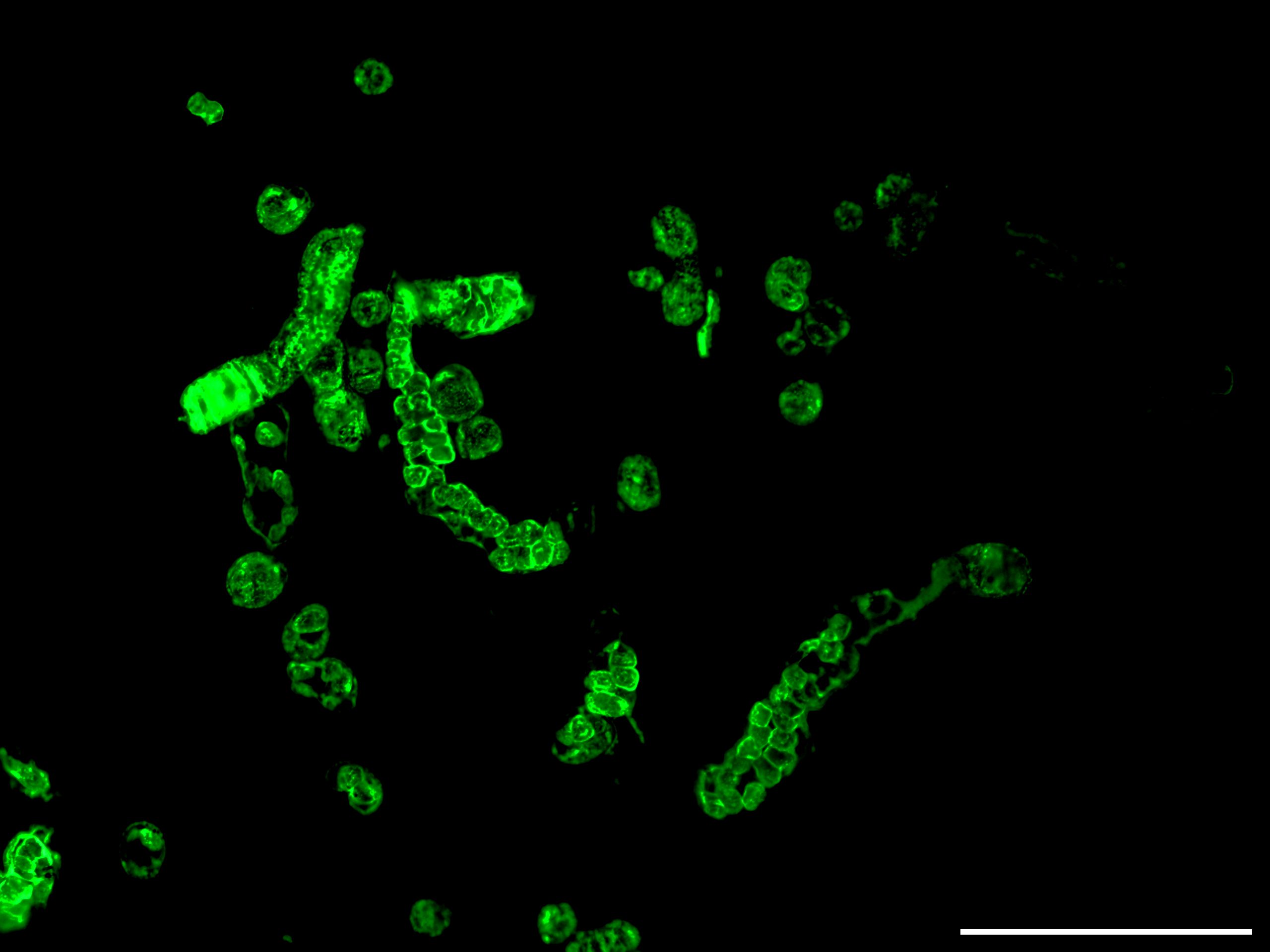

Supplement: Supplementary file 1 [file Data_Sheet_1.ZIP › 729402-supplementary material-original figures and dates-jpg-2021-7-2/729402 Fig6/Anti-rTsGS serum/Fig 6-3d AW+Anti-rTsGS serum.jpg]

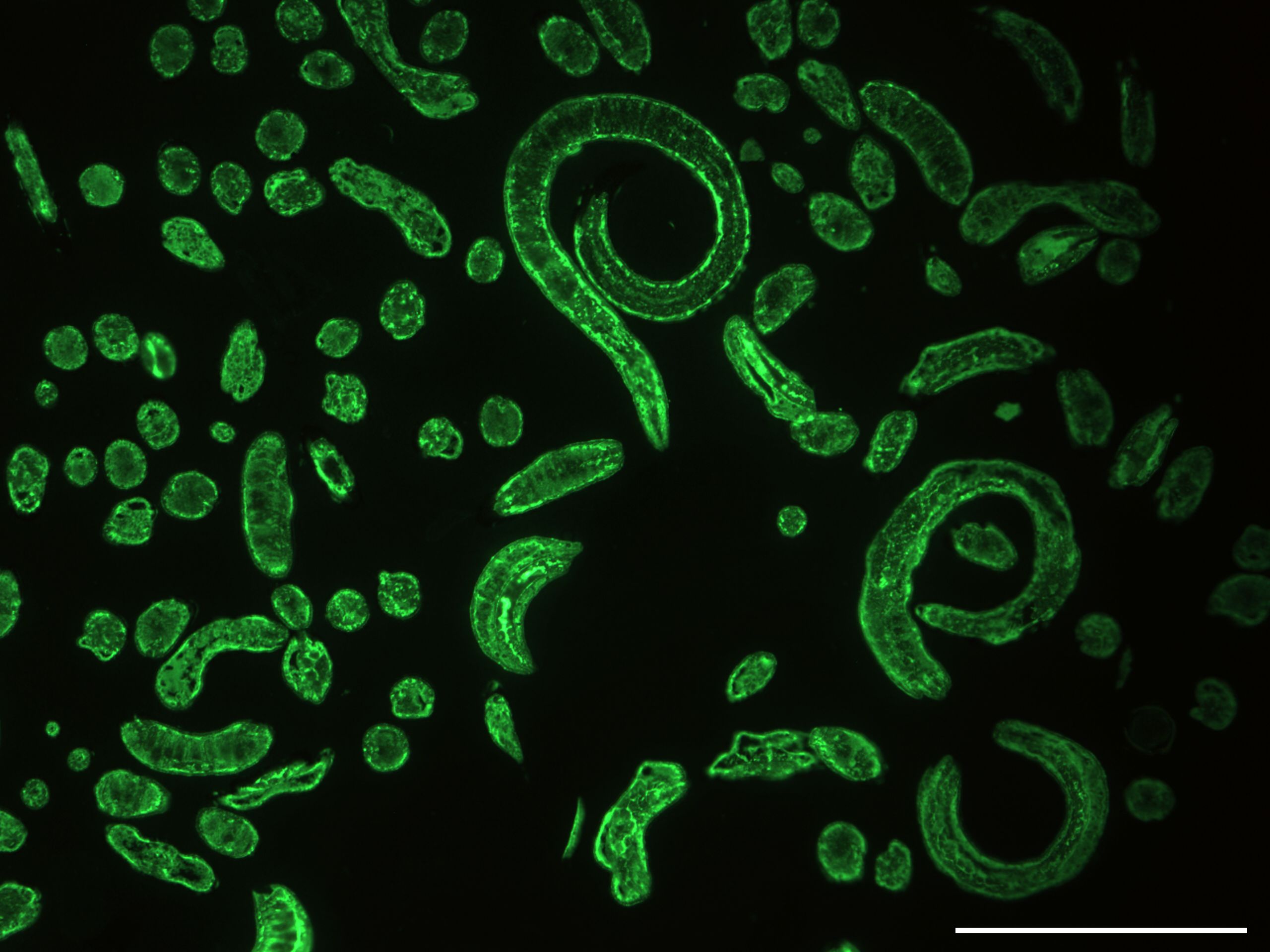

Supplement: Supplementary file 1 [file Data_Sheet_1.ZIP › 729402-supplementary material-original figures and dates-jpg-2021-7-2/729402 Fig6/Anti-rTsGS serum/Fig 6-6h IIL+Anti-rTsGS serum.jpg]

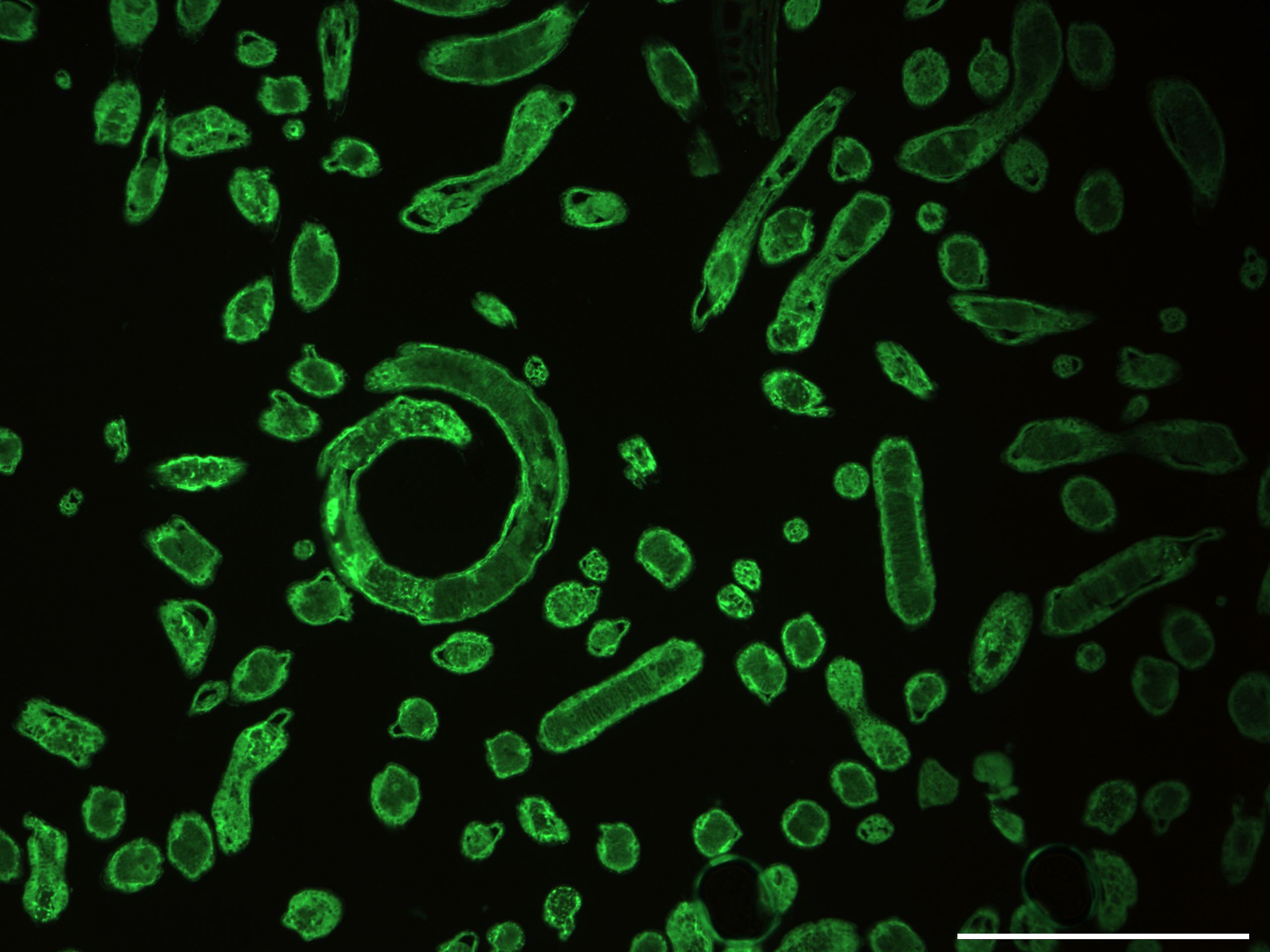

Supplement: Supplementary file 1 [file Data_Sheet_1.ZIP › 729402-supplementary material-original figures and dates-jpg-2021-7-2/729402 Fig6/Anti-rTsGS serum/Fig 6-ML+Anti-rTsGS serum.jpg]

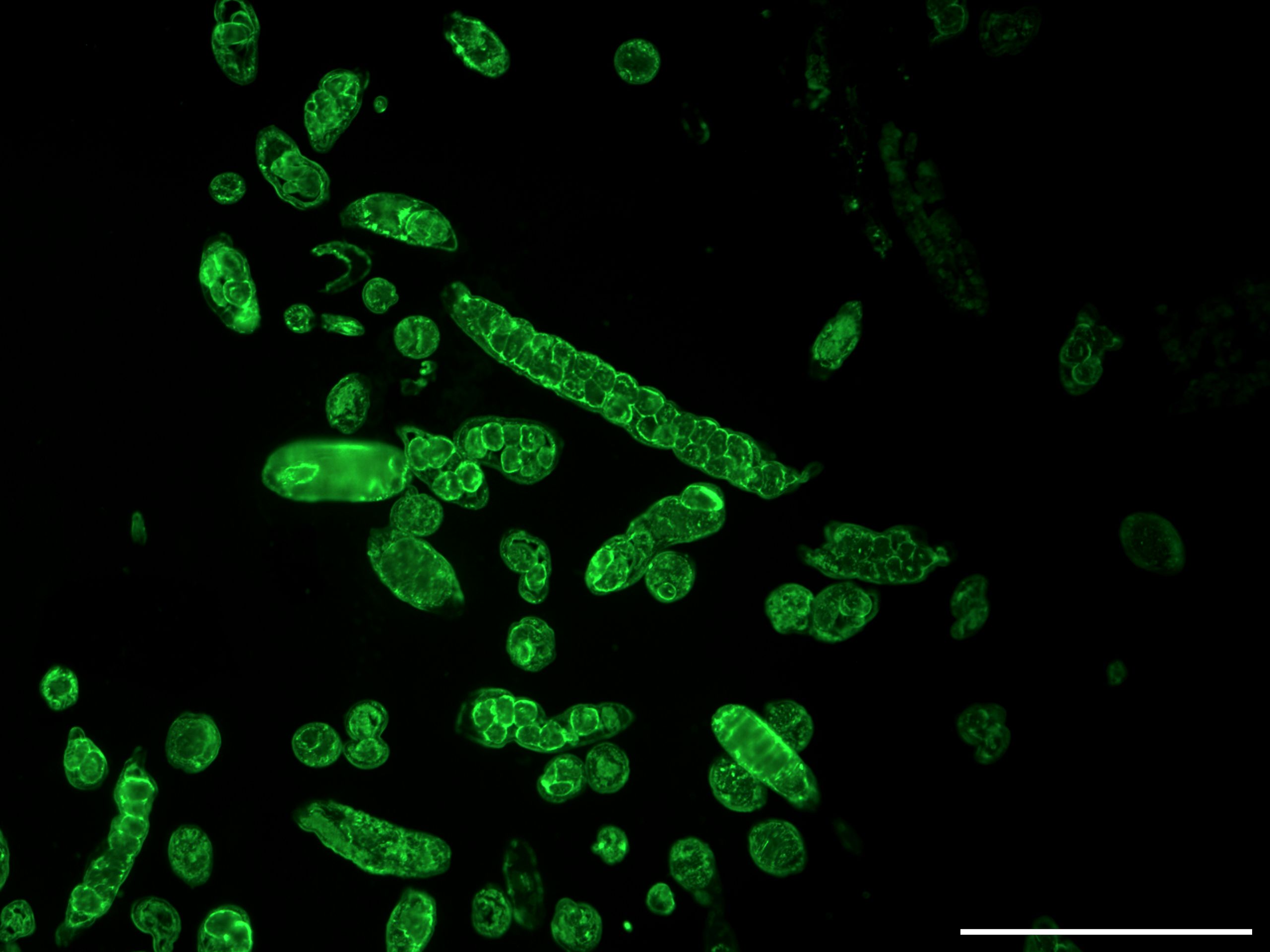

Supplement: Supplementary file 1 [file Data_Sheet_1.ZIP › 729402-supplementary material-original figures and dates-jpg-2021-7-2/729402 Fig6/Infection serum/Fig 6-3d AW+Infection serum.jpg]

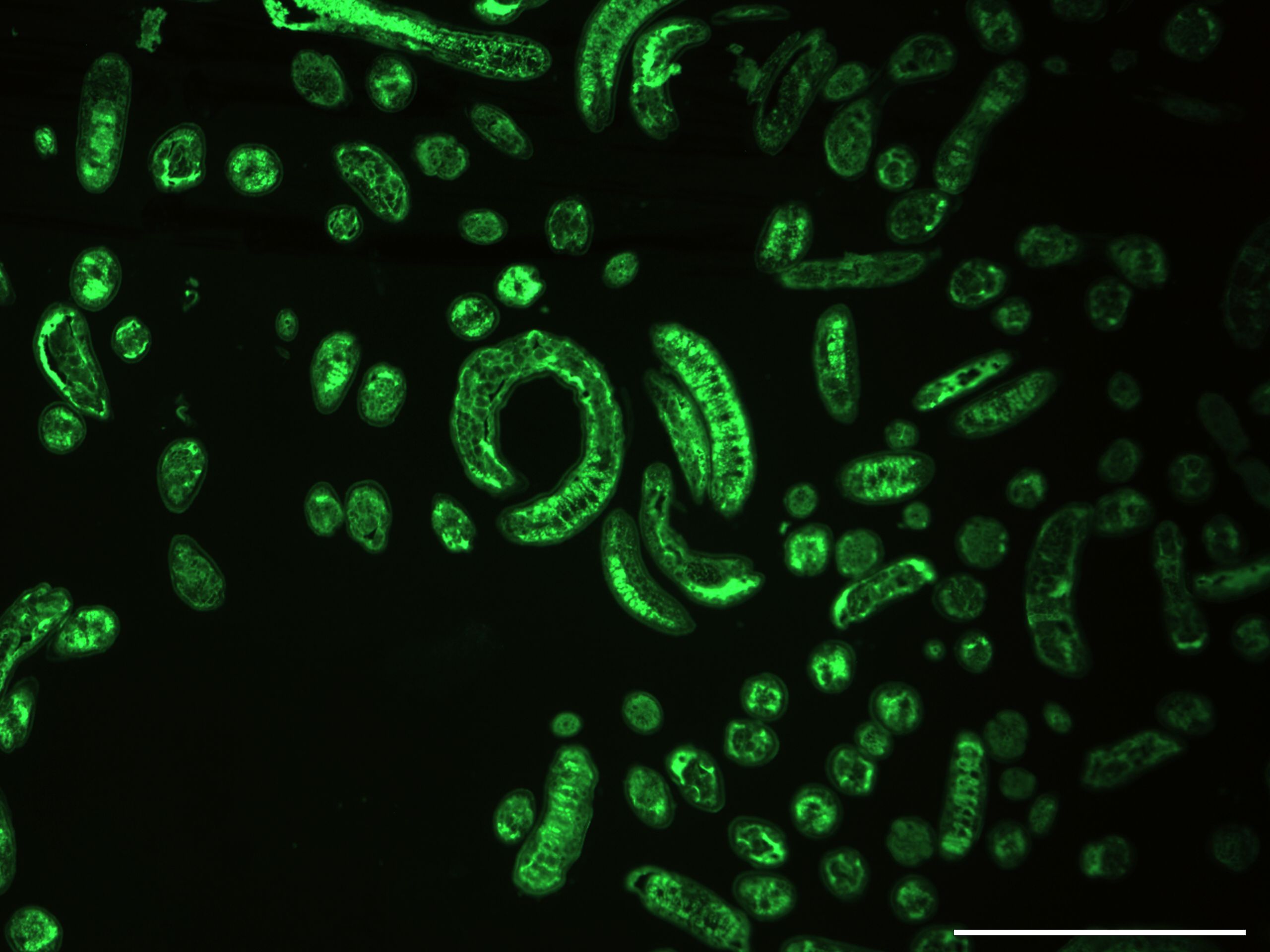

Supplement: Supplementary file 1 [file Data_Sheet_1.ZIP › 729402-supplementary material-original figures and dates-jpg-2021-7-2/729402 Fig6/Infection serum/Fig 6-6h IIL+Infection serum.jpg]

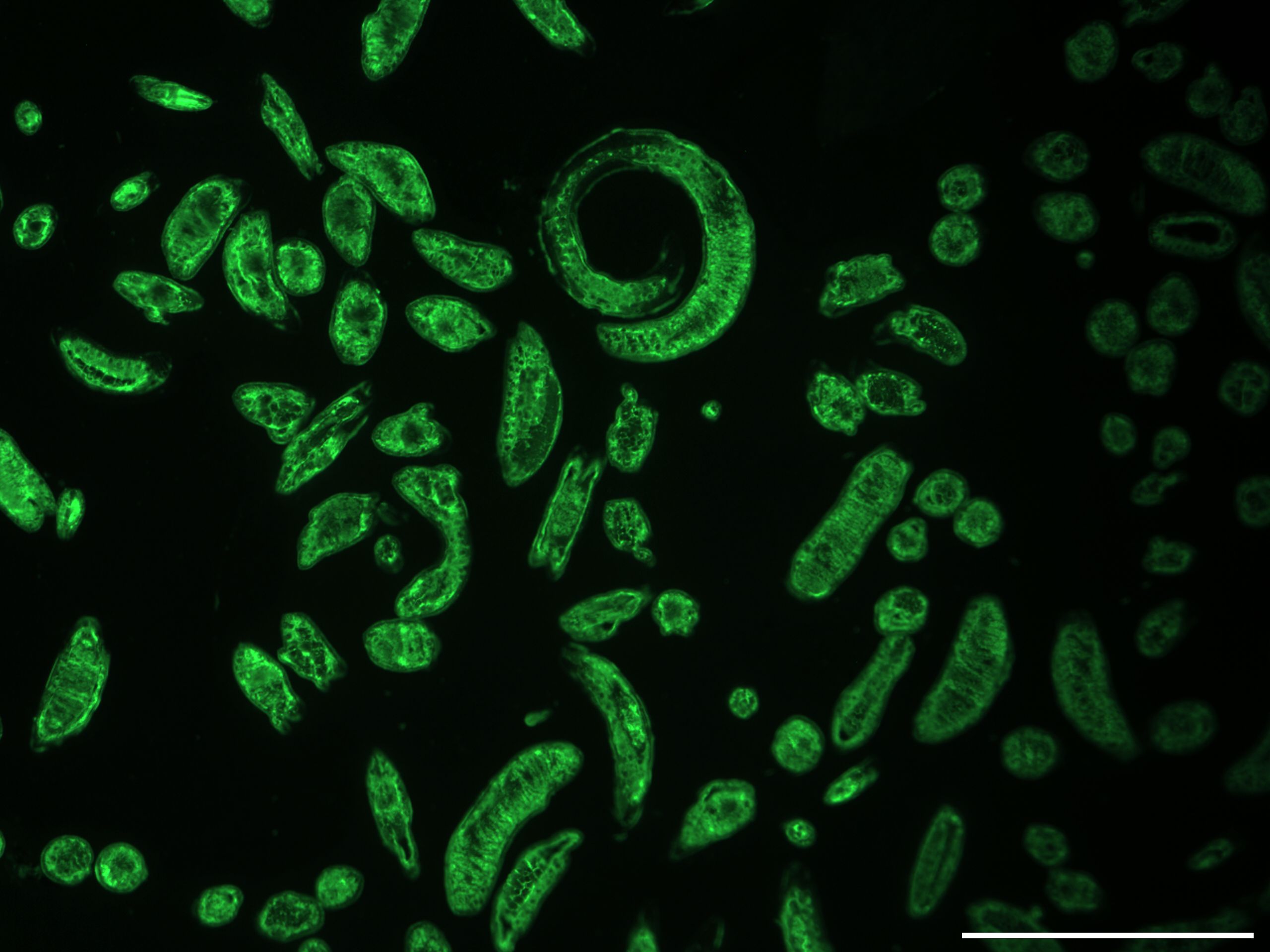

Supplement: Supplementary file 1 [file Data_Sheet_1.ZIP › 729402-supplementary material-original figures and dates-jpg-2021-7-2/729402 Fig6/Infection serum/Fig 6-ML+Infection serum.jpg]

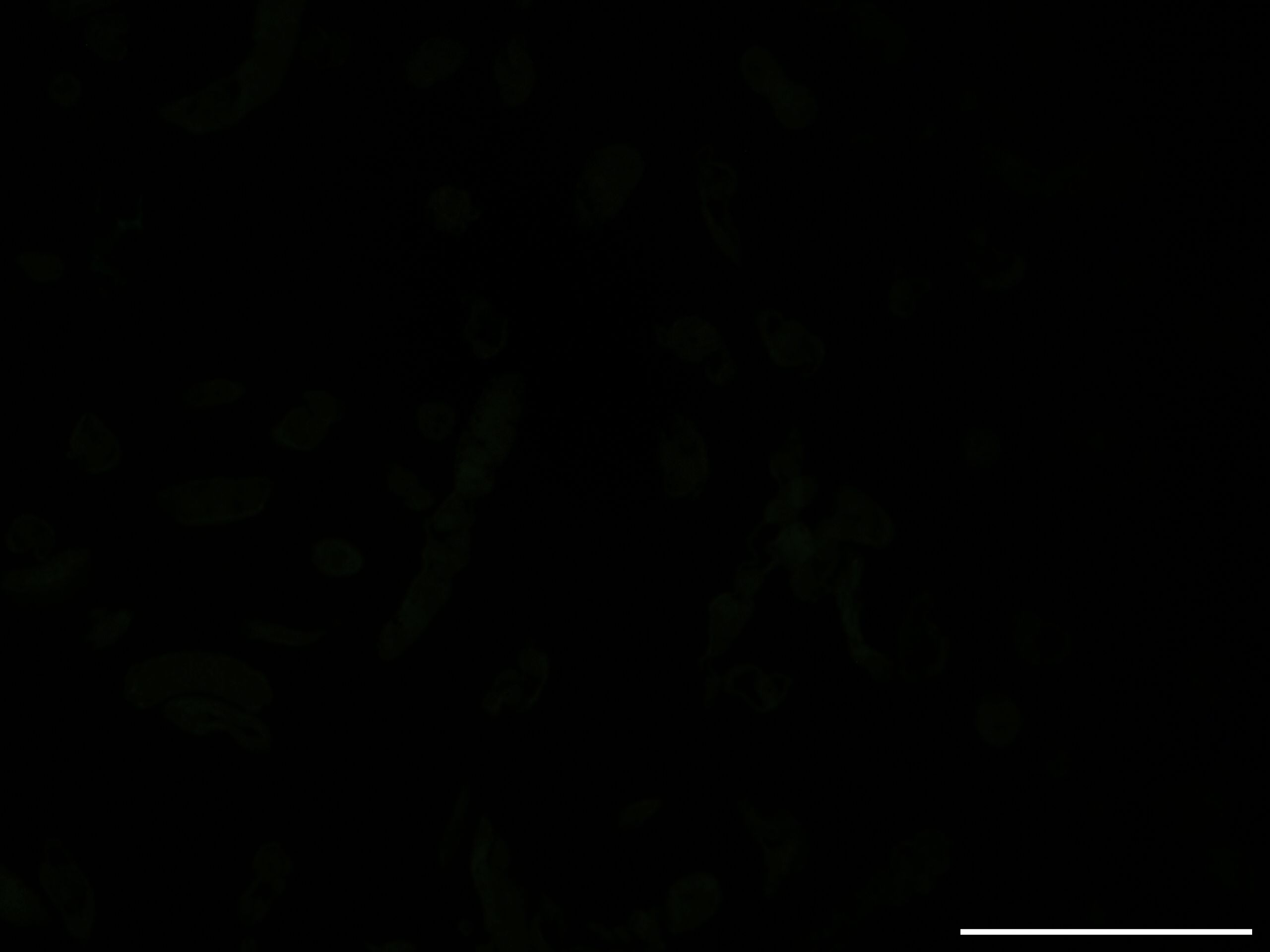

Supplement: Supplementary file 1 [file Data_Sheet_1.ZIP › 729402-supplementary material-original figures and dates-jpg-2021-7-2/729402 Fig6/Normal serum/Fig 6-3d AW+Normal serum.jpg]

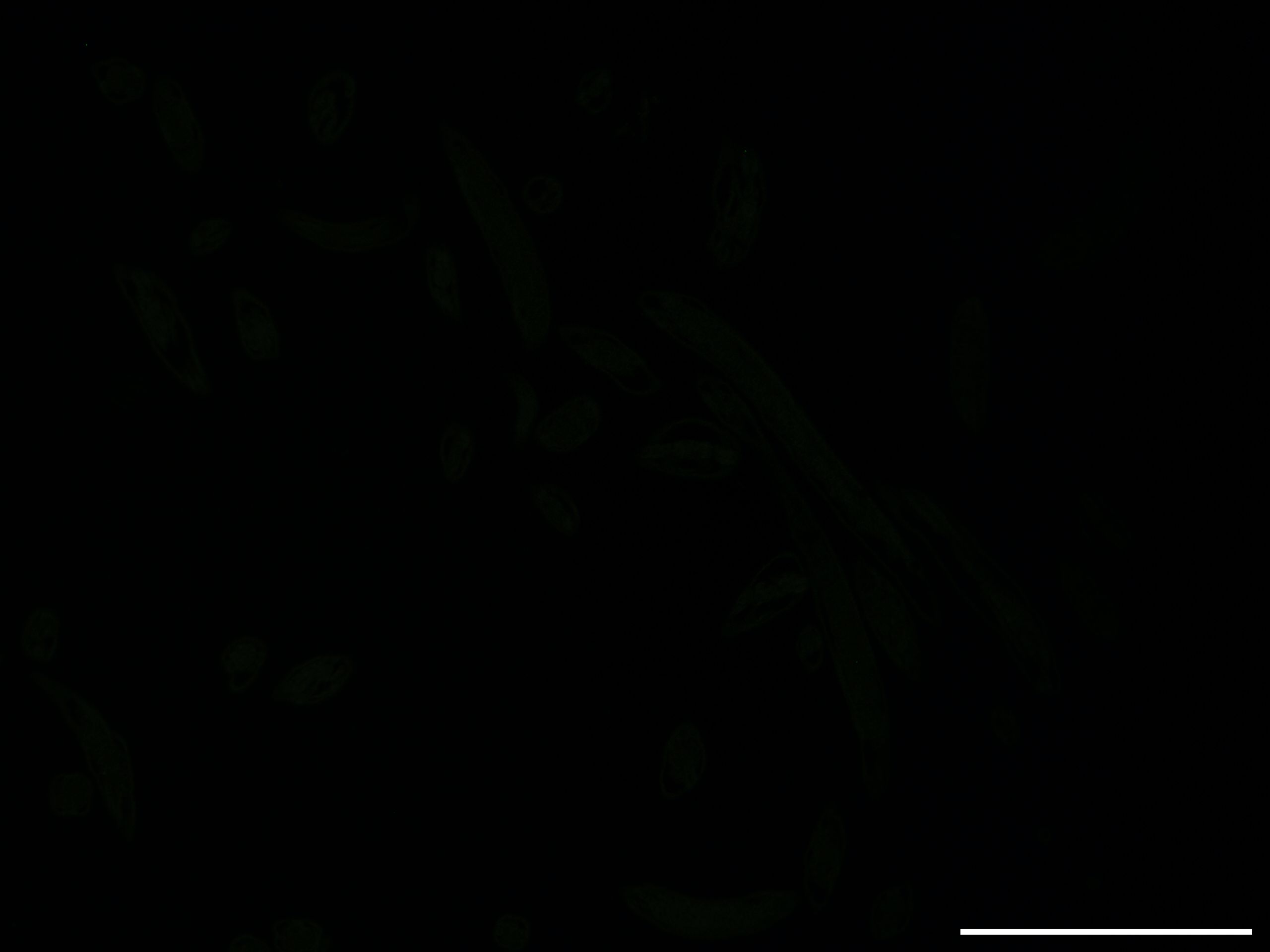

Supplement: Supplementary file 1 [file Data_Sheet_1.ZIP › 729402-supplementary material-original figures and dates-jpg-2021-7-2/729402 Fig6/Normal serum/Fig 6-6h IIL+Normal serum.jpg]

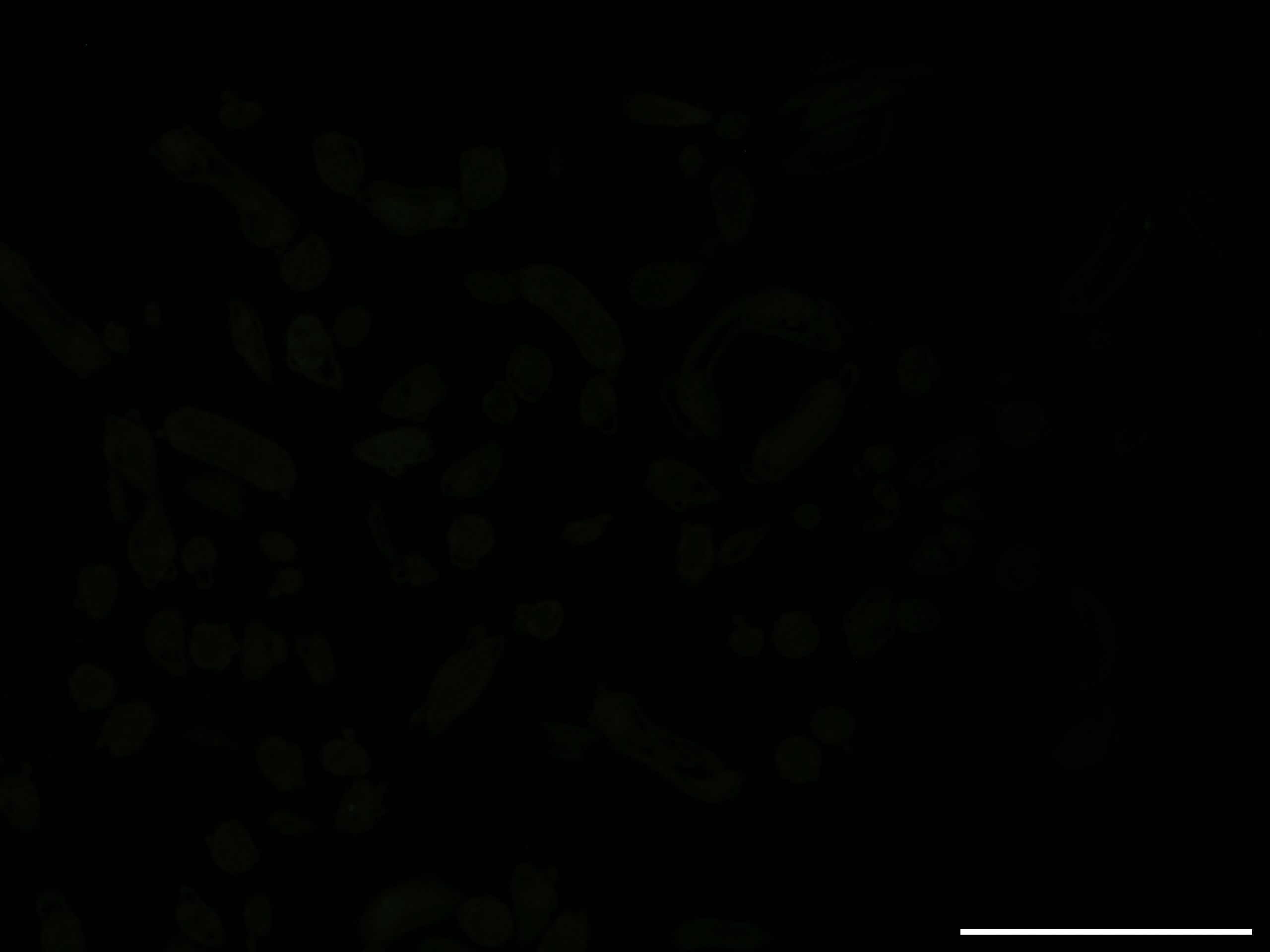

Supplement: Supplementary file 1 [file Data_Sheet_1.ZIP › 729402-supplementary material-original figures and dates-jpg-2021-7-2/729402 Fig6/Normal serum/Fig 6-ML+Normal serum.jpg]

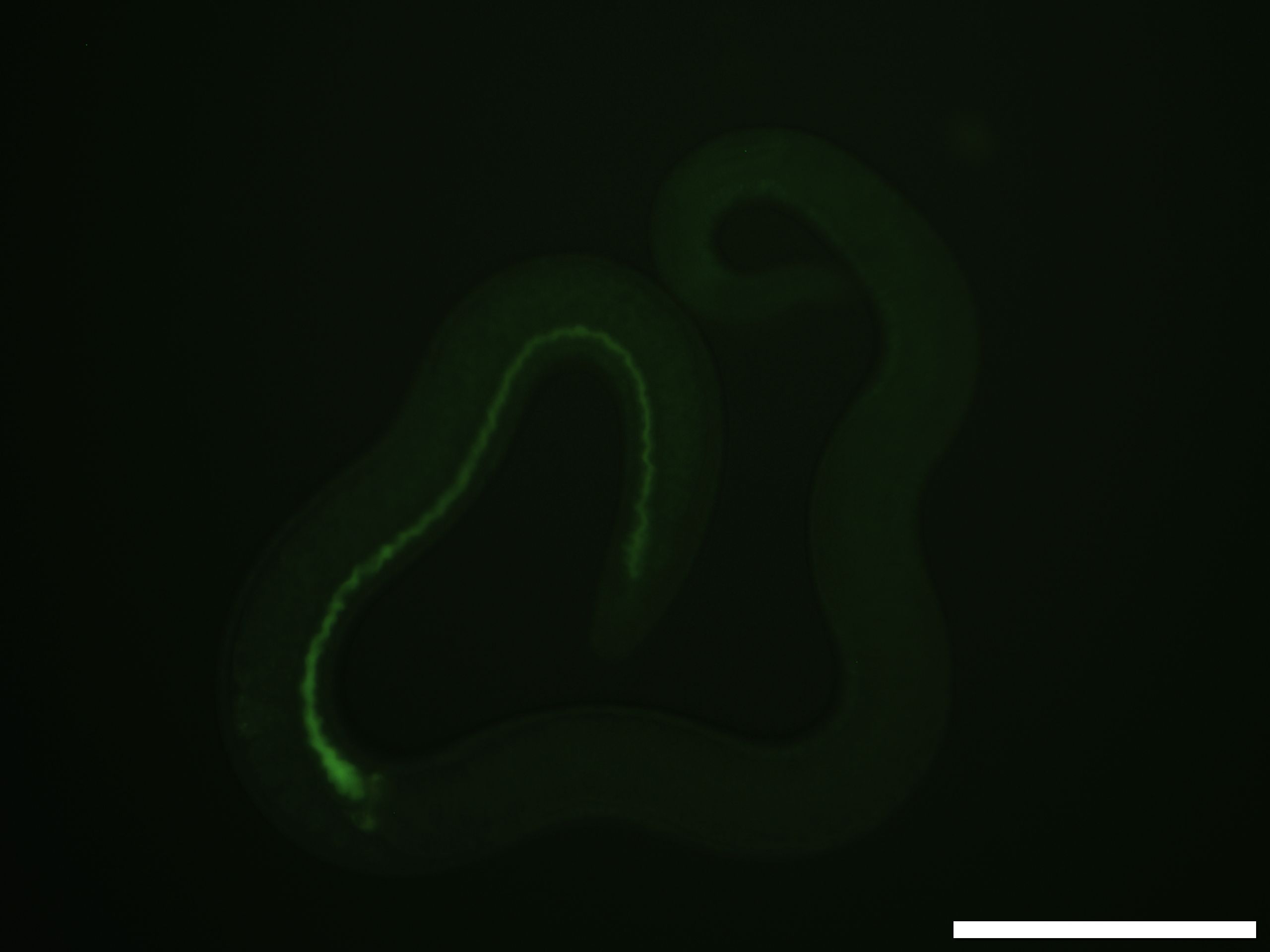

Supplement: Supplementary file 1 [file Data_Sheet_1.ZIP › 729402-supplementary material-original figures and dates-jpg-2021-7-2/729402 Fig8/Fig8-A.jpg]

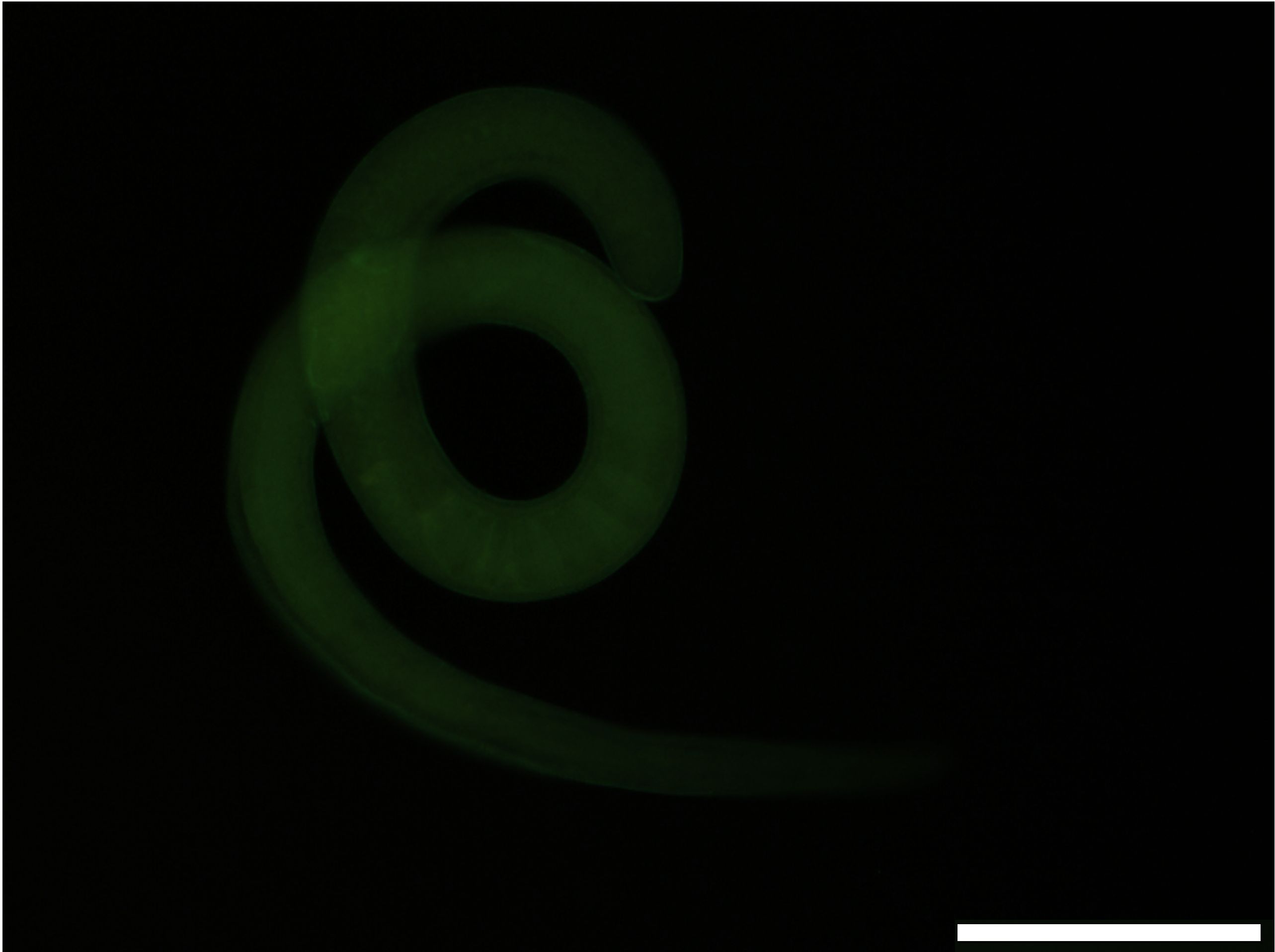

Supplement: Supplementary file 1 [file Data_Sheet_1.ZIP › 729402-supplementary material-original figures and dates-jpg-2021-7-2/729402 Fig8/Fig8-B.jpg]

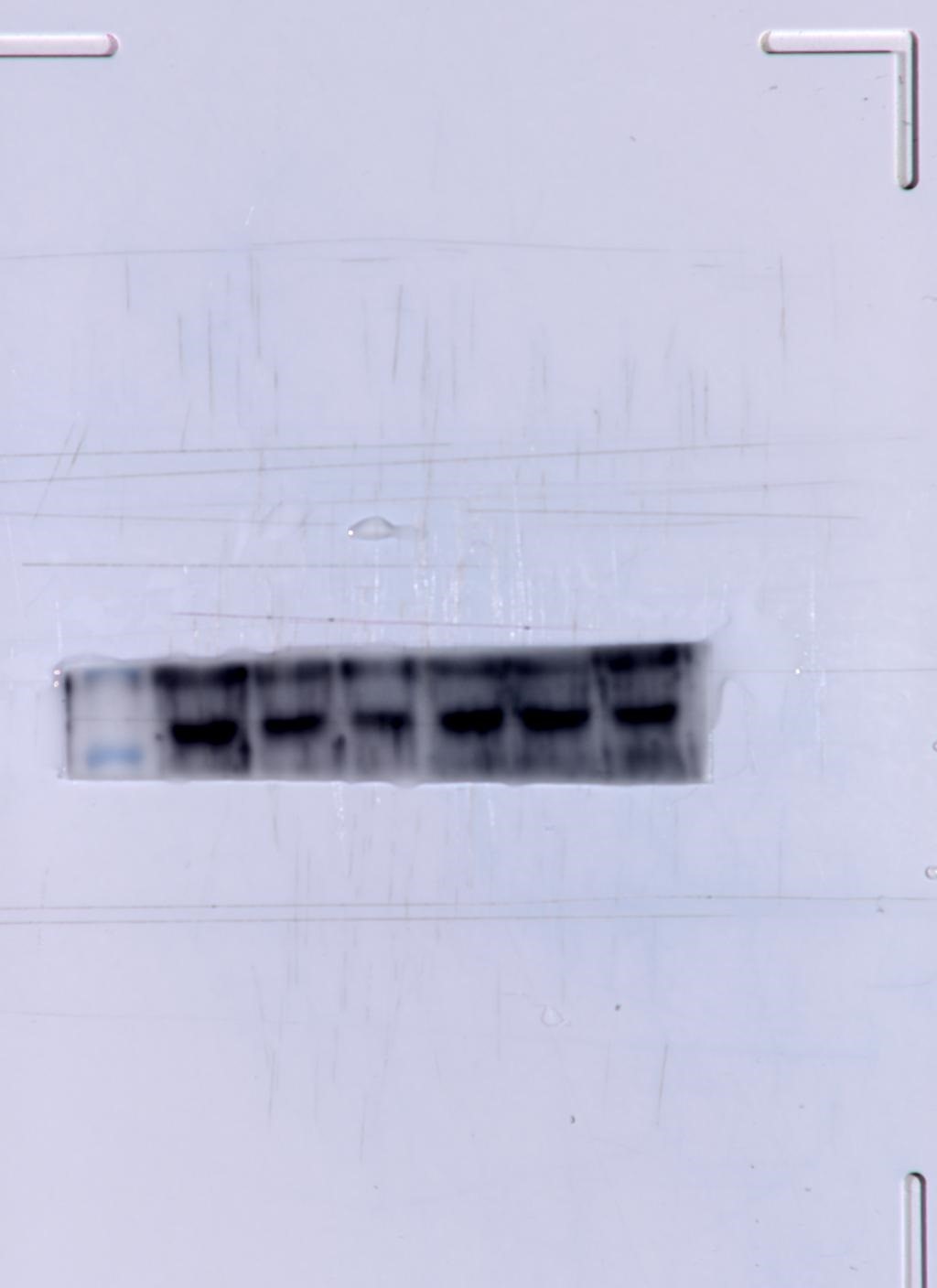

Supplement: Supplementary file 1 [file Data_Sheet_1.ZIP › 729402-supplementary material-original figures and dates-jpg-2021-7-2/729402 Fig9/Fig9-B/Fig 9B-TsGS.jpg]

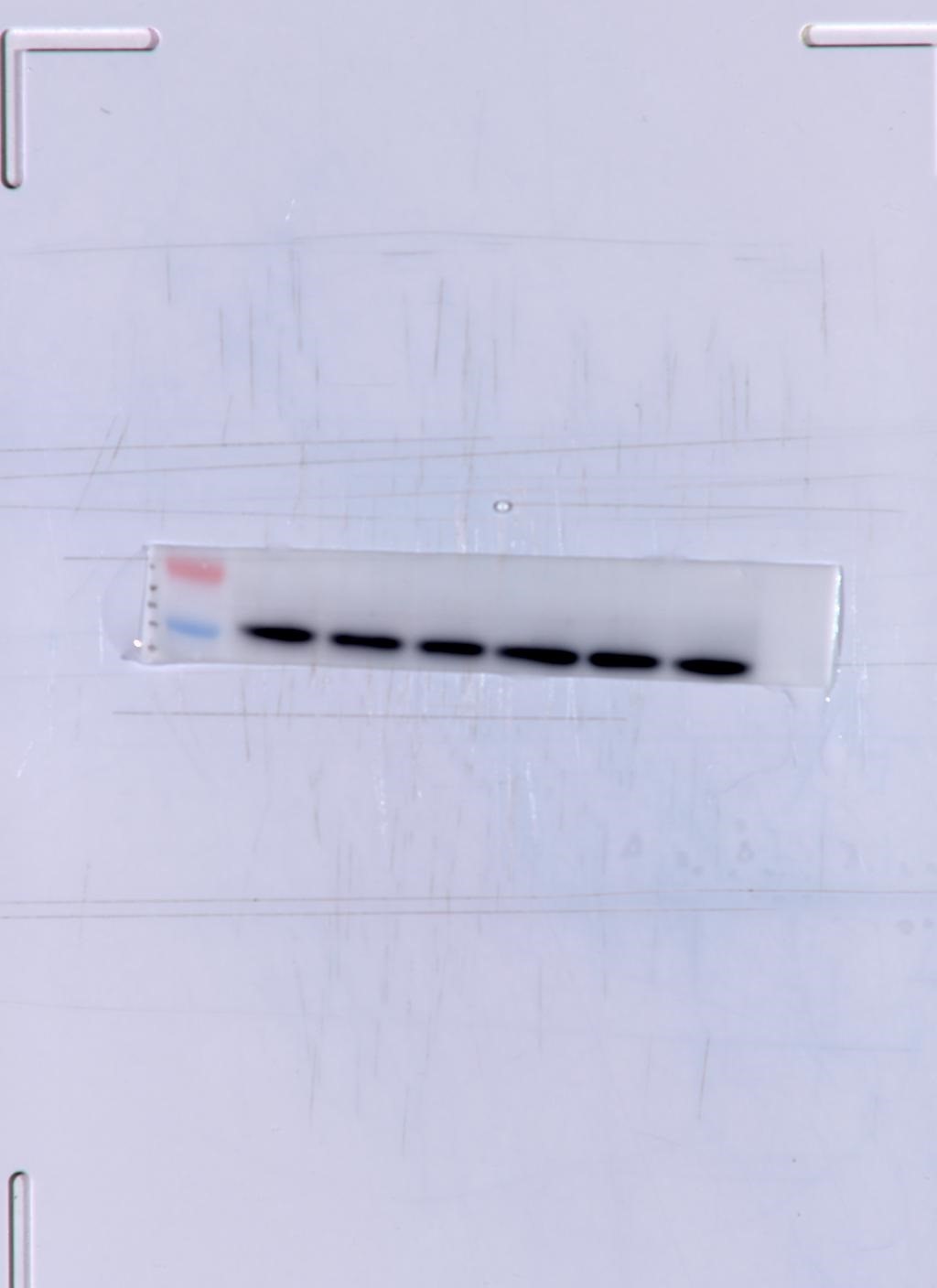

Supplement: Supplementary file 1 [file Data_Sheet_1.ZIP › 729402-supplementary material-original figures and dates-jpg-2021-7-2/729402 Fig9/Fig9-B/Fig 9B-Tubulin.jpg]

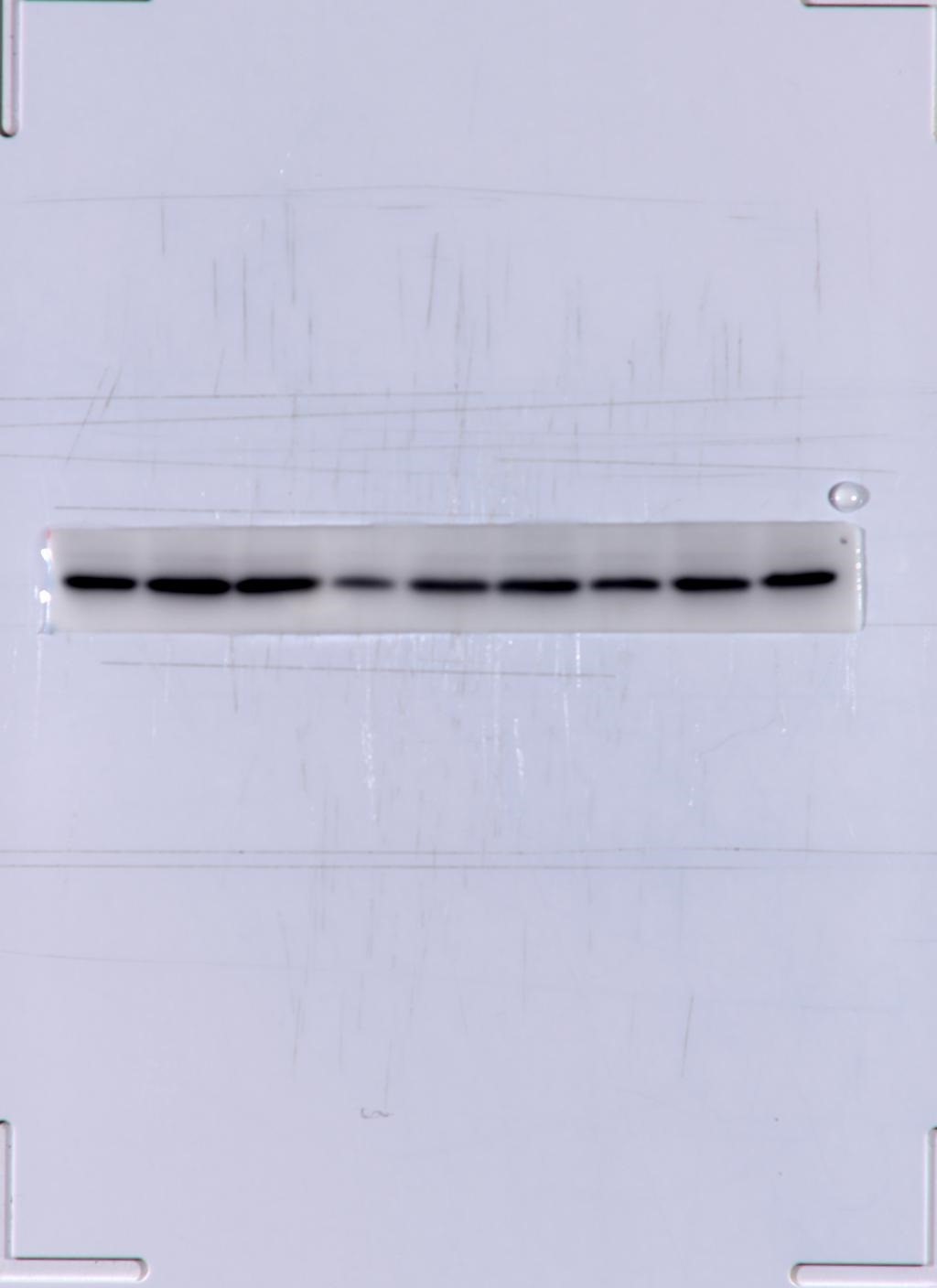

Supplement: Supplementary file 1 [file Data_Sheet_1.ZIP › 729402-supplementary material-original figures and dates-jpg-2021-7-2/729402 Fig9/Fig9-D/Fig 9D-TsGS.jpg]

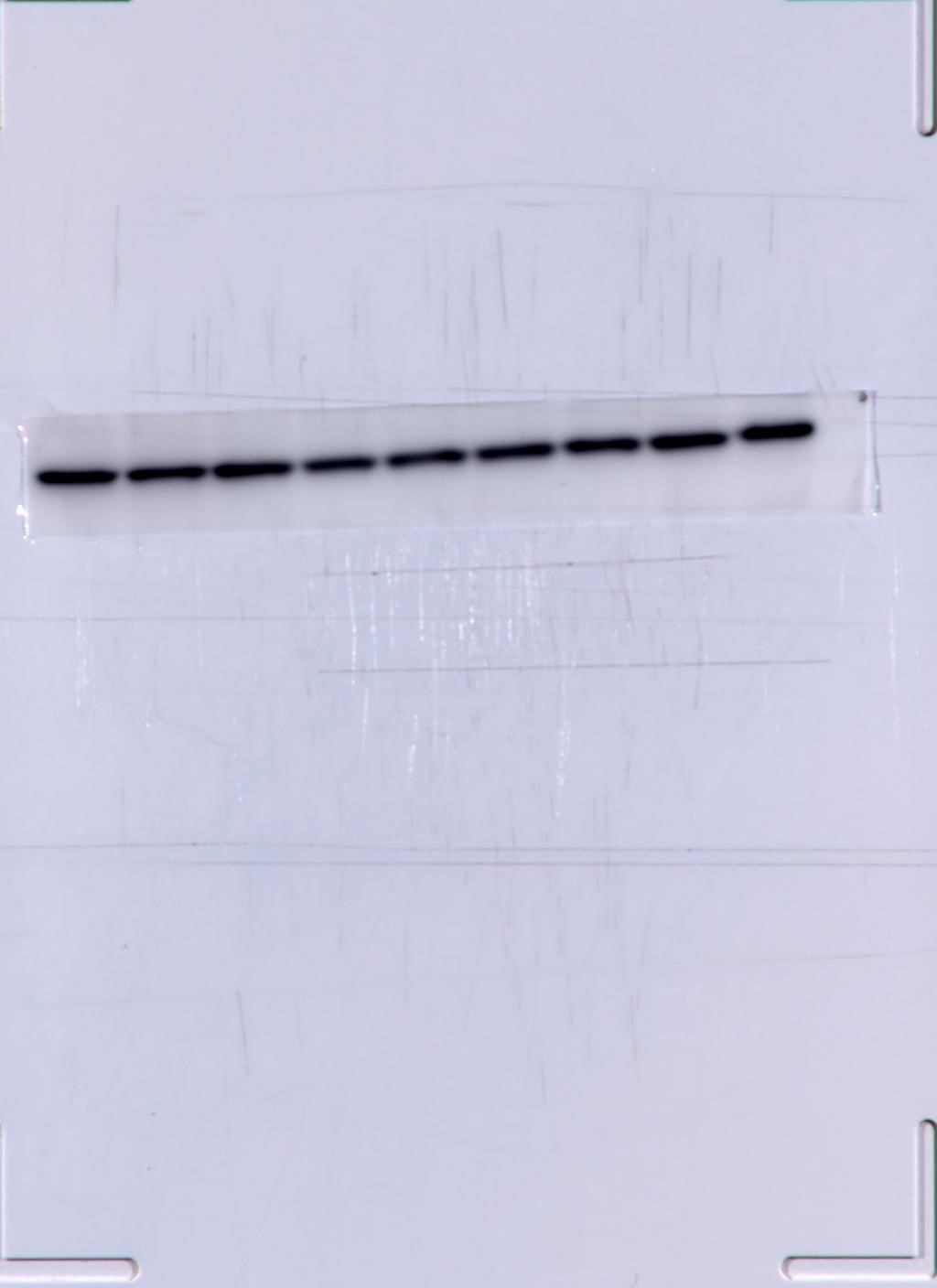

Supplement: Supplementary file 1 [file Data_Sheet_1.ZIP › 729402-supplementary material-original figures and dates-jpg-2021-7-2/729402 Fig9/Fig9-D/Fig 9D-Tubulin.jpg]

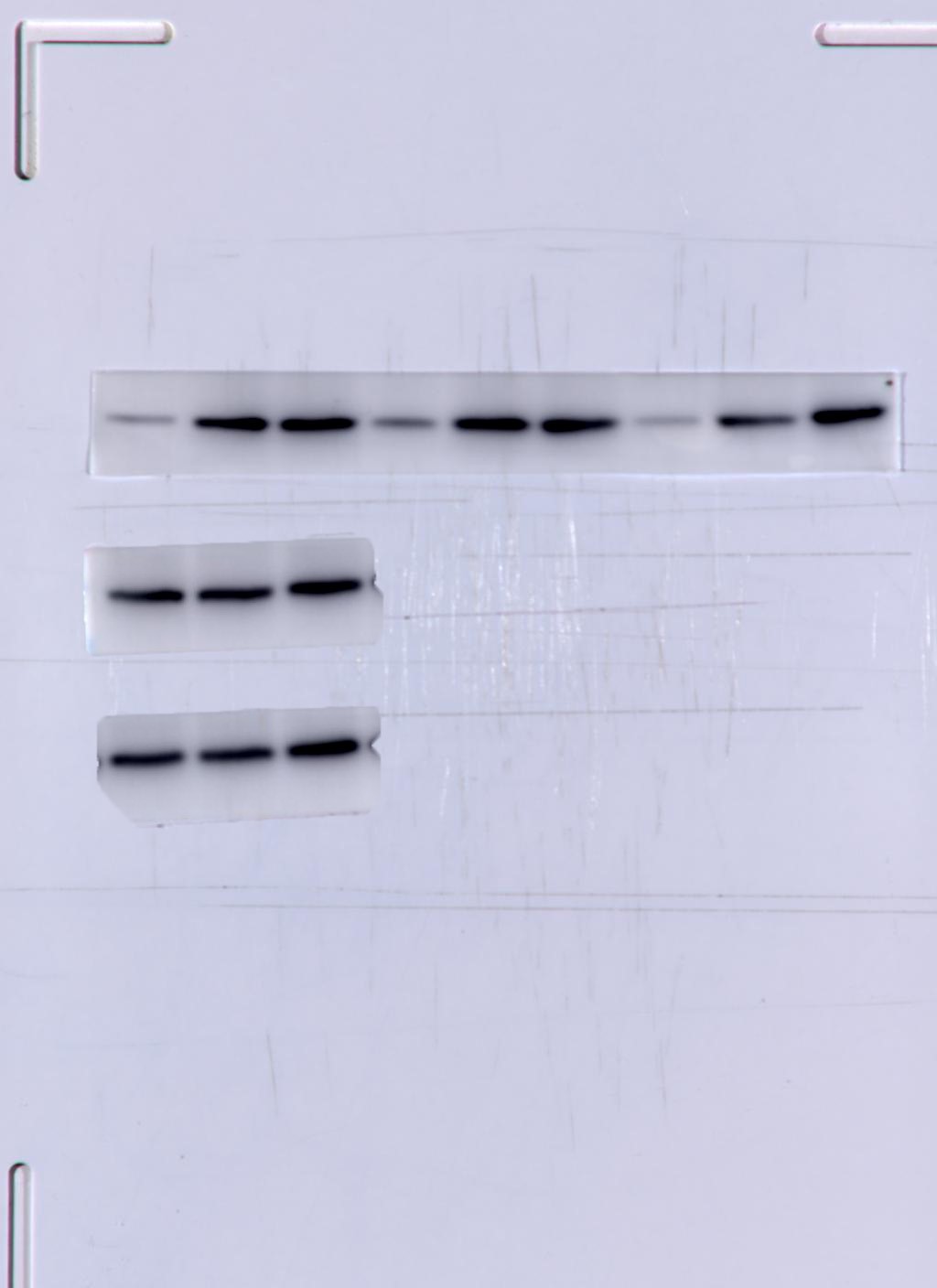

Supplement: Supplementary file 1 [file Data_Sheet_1.ZIP › 729402-supplementary material-original figures and dates-jpg-2021-7-2/729402 Fig9/Fig9-E/Fig 9E-TsGS, Tubulin and TsAPP.jpg]
